# Design, Semisynthesis, Insecticidal and Antibacterial Activities of a Series of Marine-Derived Geodin Derivatives and Their Preliminary Structure–Activity Relationships

**DOI:** 10.3390/md20020082

**Published:** 2022-01-18

**Authors:** Rong Chao, Gulab Said, Qun Zhang, Yue-Xuan Qi, Jie Hu, Cai-Juan Zheng, Ji-Yong Zheng, Chang-Lun Shao, Guang-Ying Chen, Mei-Yan Wei

**Affiliations:** 1 Key Laboratory of Marine Drugs, The Ministry of Education of China, School of Medicine and Pharmacy, Ocean University of China, Qingdao 266003, China; chaorong1122@163.com (R.C.); gulabouc@gmail.com (G.S.); zhangqunnn@163.com (Q.Z.); hujiewrc@163.com (J.H.); shaochanglun@163.com (C.-L.S.); 2 Department of Chemistry, Women University Swabi, Swabi 23430, Pakistan; 3 State Key Laboratory for Marine Corrosion and Protection, Luoyang Ship Material Research Institute (LSMRI), Qingdao 266061, China; qiyuexuan9475@163.com; 4 Key Laboratory of Tropical Medicinal Resource Chemistry of Ministry of Education, Hainan Normal University, Haikou 570100, China; caijuan2002@163.com (C.-J.Z.); chgying123@163.com (G.-Y.C.); 5 College of Food Science and Engineering, Ocean University of China, Qingdao 266003, China

**Keywords:** *Aspergillus* sp., geodin, semisynthesize, insecticidal activity, antibacterial activity

## Abstract

To enhance the biological activity of the natural product geodin (**1**), isolated from the marine-derived fungus *Aspergillus* sp., a series of new ether derivatives (**2**–**37**) was designed and semisynthesized using a high-yielding one-step reaction. In addition, the insecticidal and antibacterial activities of all geodin congeners were evaluated systematically. Most of these derivatives showed better insecticidal activities against *Helicoverpa armigera* Hübner than **1**. In particular, **15** showed potent insecticidal activity with an IC_50_ value of 89 μM, comparable to the positive control azadirachtin (IC_50_ = 70 μM). Additionally, **5**, **12**, **13**, **16**, **30** and **33** showed strong antibacterial activity against *Staphylococcus aureus* and *Aeromonas salmonicida* with MIC values in the range of 1.15–4.93 μM. The preliminary structure–activity relationships indicated that the introduction of halogenated benzyl especially fluorobenzyl, into **1** and substitution of 4-OH could be key factors in increasing the insecticidal and antibacterial activities of geodin.

## 1. Introduction

Cotton bollworm (*Helicoverpa armigera* Hübner) is one of the most destructive agricultural pests known. This organism causes severe damage to a wide range of crops, such as cotton, sunflower, okra, tomato, chickpea, maize, potato and cabbage [1,2]. *H. armigera* is native to Africa, Asia, Europe, and Australasia and has become the most globally widespread species of *Helicoverpa* [3]. Consequently, *H. armigera* Hübner is responsible for about two billion dollars in direct economic losses each year [4]. Moreover, it has developed resistance against newer chemistries and conventional insecticides because of the extensive use of these chemical insecticides [5]. With the emergence of resistance in *H. armigera* Hübner to commercial insecticides, research on new insecticides has become particularly important. Marine natural products (MNPs) continue to play highly significant roles in the pesticide development process and are potential sources of new drugs for treating plant diseases [6,7]. Introducing structural modifications to MNPs is one important strategy for drug development initiatives to improve bioavailability, enhance biological activity and reduce toxicity [8].

**Figure 1:**
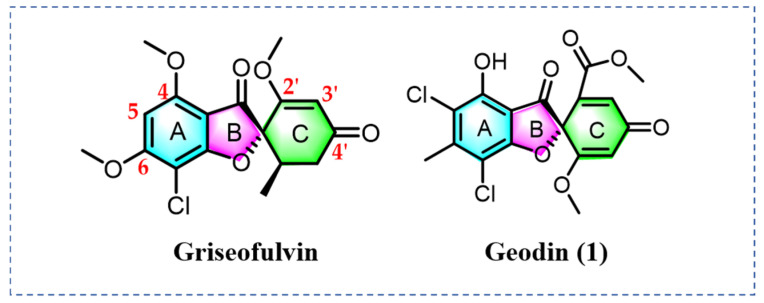
The structures of griseofulvin and geodin (**1**) which share the same grisan backbone (rings A, B and C).

During the course of our ongoing research into bioactive compounds from marine-derived fungi [9,10,11,12,13,14,15], a secondary metabolite belonging to the griseofulvin family, geodin (**1**), was isolated from the soft coral-derived fungus *Aspergillus* sp. (CHNSCLM-0151), collected from the South China Sea in 2015. Griseofulvin is a classic antifungal agent used clinically for the treatment of dermatomycoses and is an inhibitor of centrosomal clustering of cancer cell lines [16]. Griseofulvin and its analogues have been rigorously studied and some structure–activity relationships (SARs) clearly identified [16,17]. A library of 53 griseofulvin analogues with modifications on the 4, 5, 6, 2′, 3′ and 4′positions have been found to be less bioactive than the parent compound. Notably, a 2′-benzyloxy derivative with low activity against *Trichophyton rubrum* and *Trichophyton metagrophytes* was found to be among the most potent compounds identified against MDA-MB-231 cancer cells [16]. Geodin (**1**) shares the same grisan backbone as griseofulvin (Figure 1) and displays various interesting biological activities such as antimicrobial, glucose uptake for modulatory (rat adipocytes), fibrinolytic enhancement, antiviral and cytotoxic activities [18,19,20]. Most noteworthy from the current semisynthetic perspective, we have previously noted that geodin (**1**) exerts weak insecticidal activity [19]. Moreover, the previous study SAR study of **1** demonstrated that the phenolic hydroxyl group is not required for insecticidal activity [19]. In order to enhance the biological activity of **1**, 36 new derivatives of geodin (**1**) were semisynthesized to evaluate insecticidal and antimicrobial activities; using this new panel of analogs, we also have extended SAR knowledge of **1**.

## 2. Results and Discussion

### 2.1. Chemistry

The fungal strain *Aspergillus* sp. (CHNSCLM-0151) was cultivated in 50 L PDB medium at 28 °C with shaking for one week. Then the fermentation broth was extracted three times with an equal volume of EtOAc. The organic extracts were combined and concentrated under vacuum to afford a dry crude extract (35.7 g). The extract was subjected to vacuum liquid chromatography (VLC) on silica gel and eluted with a stepwise gradient of petroleum ether (PE)–EtOAc to afford six fractions (Fr. 1–Fr. 6). Fr. 5 was applied to reverse phase silica gel column and eluted with MeOH-H_2_O to obtain seven sub-fractions (Fr. 5-A and Fr. 5-G). Fr. 5-B was then purified with 65% MeOH-H_2_O to yield compound **1** (2.2 g).

The chemical structure of **1** was elucidated by analysis of NMR data and comparisons with literature [17]. Compound **1** contains an exchangeable proton, two singlet protons, two oxygenated methyl groups and a methyl. The substituents at the 4-OH were semisynthetically modified (Scheme 1) using benzyl bromide at a temperature of 40 °C for 6–12 h in the presence of K_2_CO_3_ in dry acetone to produce compounds **2**–**37** (Figure 1 and Table 1). The structures of these derivatives (**2**–**37**) were fully characterized by extensive spectroscopic analyses (Figures S1–S111).

### 2.2. Insecticidal Activity against Helicoverpa armigera Hübner

**Figure 2:**
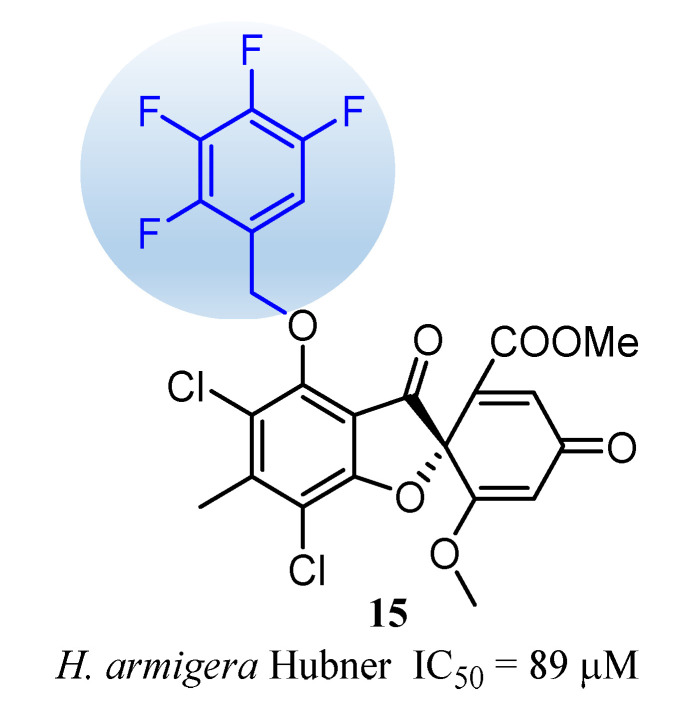
The derivative **15** with significant insecticidal activity.

In this study, the insecticidal activities against *Helicoverpa armigera* Hübner of geodin (**1**) and 36 new derivatives modified at the 4-OH position were assessed. Most derivatives showed better insecticidal activity than marine natural product **1** (Table 2). Most of the derivatives were functionalized with benzyl analogues except for **37** which was modified with only an ethyl group and had no insecticidal activity. Compounds **2**–**4** and **35** were modified with bromobenzyl moieties and exhibited moderate insecticidal activity with IC_50_ values of 176 μM (although **4** was inactive). In addition, the introduction of chlorobenzyl groups at the 4-OH position of **1** afforded the analogues **5**–**9** and **31**–**34** most of which showed higher insecticidal activity. However, the 2-chlorobenzyl derivative **5**, 2-chloro-4-fluorobenzyl derivative **31** and 4-chloro-2-fluorobenzyl derivative **33** displayed lower activity against *H. armigera* Hübner compared to parent **1**. Besides, the iodobenzyl analogues **10** and **11** had no insecticidal activity. Multiple studies have demonstrated that the introduction of a fluorine atom was useful for increasing insecticidal activity because of its unique properties such as electronegativity, size, and electrostatic interactions [21,22,23,24,25]. Herein, **12**–**21** were modified by fluorinated benzyl and most exhibited stronger activity (**16** and **19** were inactive). Especially, 2,3,4,5-tetrafluorobenzyl derivative **15** exhibited potent insecticidal activity comparable with the positive control azadirachtin with an IC_50_ value of 89 μM (Figure 2). Moreover, the result of the antifouling activity of **15** against *Navicula exigua* and the settlement of the *Mytilus edulis* showed inactive and indicated **15** was non-toxic. In addition, non-halogenated benzyl derivatives **22**–**30** which were modified with nitro, methyl, phenyl and nitrile groups had good insecticidal activity except for 3-methylbenzyl compound **24** and 2-cyanobenzyl compound **30**. The above results indicated that the introduction of benzyl especially halogenated benzyl could enhance the insecticidal activity of **1**. Strikingly, further research on **15** was worth developing the low-toxicity and high-efficiency insecticide drug.

### 2.3. Antibacterial Activity

Compounds **1**–**37** were also tested for their pharmacological activities against three bacteria, *Staphylococcus aureus, Aeromonas salmonicida* and *Pseudomonas aeruginosa* (Table 2). For *S. aureus*, 4-bromobenzyl derivative **4** and 3-iodobenzyl derivative **10** showed weak activity with MIC values of 17.60 and 16.25 μM, respectively (positive control ciprofloxacin, MIC = 0.16 μM). The chlorobenzyl analogues **5**, **6** and **8** exhibited strong inhibitory activities with MIC values of 4.77, 9.55 and 8.96 μM, respectively. In addition, the introduction of fluorine atom improved the activity (**12**–**20**, MIC values: 4.76 to 17.94 μM). The cyanobenzyl derivatives **29** and **30** showed strong inhibitory activities (MIC values of 9.72 and 4.86 μM), while other non-halogenated benzyl derivatives **22**–**28** were inactive. Moreover, the simultaneous introduction of fluorine and chlorine atoms significantly enhanced antibacterial activity (**31**–**34**, MIC values: 4.62 to 9.23 μM) while 4-bromo-2-fluorobenzyl derivative **35** was inactive. At the same time, 2-cyano-5-fluorobenzyl analogue **36** exhibited moderate inhibitory activity with an MIC value of 18.97 μM.

**Figure 3:**
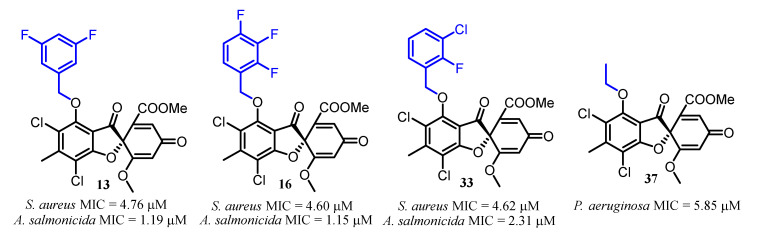
The selective derivatives with strong antibacterial activities.

For *A. salmonicida*, similarly, the introduction of halogenated benzyl strengthened the activity of geodin (**1**). Almost all of the compounds modified by benzyl groups substituted with fluorine, chlorine, bromine and iodine atoms showed better antibacterial activity than the parent **1**. The derivatives **2**, **5**, **6**, **8**–**10**, **13**–**16**, **20**, **21**, **31**, **33** and **35** showed strong inhibitory activities with MIC values in the range of 1.15 to 4.77 μM (Figure 3), while **4**, **11**, **19** and **32** showed moderate inhibitory activities against *A. salmonicida* with MIC values ranging from 8.13 to 9.23 μM (positive control Sea-Nine 211, MIC = 0.27 μM). At the same time, the non-halogenated benzyl analogues **26** and **30** also exhibited strong activities with MIC values of 2.29 and 4.86 μM.

Interestingly, the ethyl derivative **37** showed selective inhibitory activity against *P. aeruginosa* with an MIC value of 5.85 μM and the positive control Sea-Nine 211 had an MIC value of 0.27 μM (Figure 3). The results revealed that the halogenated benzyl modification on 4-OH of geodin (**1**) improved the antibacterial activity against *S. aureus* and *A. salmonicida*. At the same time, the introduction of ethyl on the 4-OH position of geodin showed selective inhibitory activities against *P. aeruginosa*.

## 3. Materials and Methods

### 3.1. General Experimental Procedures

^1^H and ^13^C NMR spectra were measured on an Agilent DD2 NMR spectrometer at 500 MHz and 125 MHz frequencies, respectively. For vacuum column chromatography silica gel (200–300 mesh, Qing Dao Hai Yang Chemical Group Co., Qingdao, China) and silica gel plates for thin layer chromatography (G60, F-254, and Yan Tai Zi Fu Chemical Group Co., Yan Tai, China) were used. HR-ESI-MS spectra were recorded on a Micro-mass Q-TOF spectrometer while UPLCMS spectra were measured on Waters UPLC^®^ system using a C18 column [ACQUITY UPLC^®^ BEH C18, 2.1 × 50 mm, 1.7 μm; 0.5 mL/min] and ACQUITY QDA ESIMS scan from 150 to 1000 Da. For reverse phase Octadecylsilyl silica gel column was used. All the derivatives of compound **1** were semisynthesized by one step reaction. The products formation and reaction completion were checked by TLC at various intervals of time.

### 3.2. Biological Material

*Aspergillus* sp. (CHNSCLM-0151) was isolated from the inner soft fresh tissue of a soft coral *Sinularia* sp., collected in 2015 from the coastal depth of the South China Sea. It was identified as *Aspergillus* sp., on the basis of its morphological and RNA base sequences. The fungus 512 base pair had 98% ITS sequence similarity with *Aspergillus* sp., NRRL58570 (GenBank No. HQ288052.1). The sequence data have been submitted to GenBank with accession number KY235298. The strain has been stored at the Key Laboratory of Marine Drugs, the Ministry of Education of China, School of Medicine and Pharmacy, Ocean University of China, Qingdao, China.

### 3.3. Extraction and Isolation

The pure spores of fungal strain were streaked onto PDA plates and incubated at 28 °C for one week. After the growth of fungus, small plugs of PDA containing the spores were aseptically put into 1000 mL Erlenmeyer flasks having about 60 g sterilized rice medium and 2% sodium chloride salt. The flasks were fermented at room temperature for 3 weeks, extracted three times with EtOAc, and evaporated under vacuum. The crude extract was subjected to normal phase silica gel column chromatography (CC) (200–300 mesh), eluting with a linear gradient of PE-EtOAc (*v*/*v*, gradient) to afford five fractions (Fr. 1–Fr. 5). Fraction 4 eluted with 40% EtOAc was further subjected to reverse phase silica gel CC, using MeOH and H_2_O as mobile phase. Compound **1** was eluted with 80% MeOH/H_2_O and further purified by recrystallization in MeOH/CH_2_Cl_2_.

#### Geodin (1)

White, solid; ^1^H NMR (500 MHz, CDCl_3_) *δ* 7.47 (1H, s, OH-4), 7.14 (1H, d, *J* = 1.0 Hz, H-3′), 5.82 (1H, d, *J* = 1.0 Hz, H-5′), 3.73 (3H, s, H-7′), 3.70 (3H, s, H-9′), 2.57 (3H, s, H-7); ^13^C NMR (125 MHz, CDCl_3_) *δ* 193.4 (C-8), 185.1 (C-4′), 168.0 (C-6′), 165.6 (C-2), 163.5 (C-8′), 149.5 (C-4), 146.7 (C-6), 137.6 (C-3′), 137.0 (C-2′), 114.9 (C-5), 109.6 (C-3), 109.0 (C-1), 104.5 (C-5′), 84.6 (C-1′), 57.1 (C-7′), 53.2 (C-9′), 18.9 (C-7), ESIMS *m*/*z* 398.9 [M + H]^+^.

### 3.4. General Synthetic Methods for Compounds **2**–**37**

A corresponding benzyl bromide reagent (3–5 eq.) and anhydrous K_2_CO_3_ (15 mg) were added to a stirred solution of **1** (40 mg, 0.10 mmol) in dry acetone (20 mL). The reaction mixture was stirred at 40 °C for 6–12 h. After the completion of the reaction, water was added to the reaction mixture, then the solution was extracted two times with EtOAc (40 mL). The organic layer was combined and dried under vacuum to give a crude residue which was purified through normal phase silica gel CC (200–300 mesh) eluting with a linear gradient of PE-EtOAc.

#### 3.4.1. Characterization Data of Compounds **2**–**37**

##### Methyl(R)-4-((2-bromobenzyl)oxy)-5,7-dichloro-6′-methoxy-6-methyl-3,4′-dioxo-3H-spiro[benzofuran-2,1′-cyclohexane]-2′,5′-diene-2′-carboxylate (**2**)

White, solid; yield 88.8%; ^1^H NMR (500 MHz, CDCl_3_) *δ* 7.76 (1H, d, *J* = 7.5 Hz), 7.56 (1H, d, *J* = 8.0 Hz), 7.35 (1H, t, *J* = 7.5 Hz), 7.20 (1H, t, *J* = 8.0 Hz), 7.14 (1H, d, *J* = 1.0 Hz, H-3′), 5.82(1H, s, H-5′), 5.45 (1H, d, *J* = 12.0 Hz), 5.40 (1H, d, *J* = 12.0 Hz), 3.75 (3H, s, H-7′), 3.70 (3H, s, H-9′), 2.59 (3H, s, H-7); ^13^C NMR (125 MHz, CDCl_3_) *δ* 190.5 (C-8), 185.3 (C-4′), 168.4 (C-6′), 167.0 (C-2), 163.6 (C-8′), 151.1 (C-4), 146.1 (C-6), 137.7 (CH), 137.6 (C-3′), 135.9 (C-2′), 132.7 (CH), 130.3 (CH), 129.9 (CH), 127.7 (CH), 123.2 (C), 122.2 (C-5), 113.6 (C-3), 113.3 (C-1), 104.5 (C-5′), 84.3 (C-1′), 76.4 (CH_2_), 57.1 (C-7′), 53.2 (C-9′), 19.1 (C-7); HRESIMS *m*/*z* 566.9614 [M + H]^+^ (calcd for C_24_H_18_BrCl_2_O_7_^+^, 566.9607).

##### Methyl(R)-4-((3-bromobenzyl)oxy)-5,7-dichloro-6′-methoxy-6-methyl-3,4′-dioxo-3Hspiro [benzofuran-2, 1′-cyclohexane]-2′,5′-diene-2′-carboxylate (**3**)

White, solid; yield 50.0%; ^1^H NMR (500 MHz, CDCl_3_) *δ* 7.68 (1H, s), 7.47 (2H, *t*, *J* = 8.4 Hz), 7.23 (1H, d, *J* = 7.8 Hz), 7.15 (1H, d, *J* = 1.5 Hz, H-3′), 5.82 (1H, d, *J* = 1.5 Hz, H-5′), 5.34 (1H, d, *J* = 11.2 Hz), 5.25 (1H, d, *J* = 11.2 Hz), 3.75 (3H, s, H-7′), 3.69 (3H, s, H-9′), 2.59 (3H, s, H-7); ^13^C NMR (125 MHz, CDCl_3_) *δ* 190.7 (C-8), 185.3 (C-4′), 168.4 (C-6′), 167.1 (C-2), 163.7 (C-8′), 150.6 (C-4), 146.2 (C-6), 138.5 (C), 137.8 (C-3′), 137.5 (C-2′), 131.7 (CH), 131.6 (CH), 130.2 (CH), 127.2 (CH), 122.6 (C), 113.8 (C-5), 113.5 (C-3), 109.0 (C-1), 104.5 (C-5′), 84.3 (C-1′), 75.8 (CH_2_), 57.1 (C-7′), 53.3 (C-9′), 19.1 (C-7); HRESIMS *m*/*z* 566.9612 [M + H]^+^ (calcd for C_24_H_18_BrCl_2_O_7_^+^, 566.9607).

##### Methyl(R)-4-((4-bromobenzyl)oxy)-5,7-dichloro-6′-methoxy-6-methyl-3,4′-dioxo-3H-spiro[benzofuran-2,1′-cyclohexane]-2′,5′-diene-2′-carboxylate (**4**)

White, solid; yield 72.7%; ^1^H NMR (500 MHz, CDCl_3_) *δ* 7.48 (2H, d, *J* = 8.5 Hz), 7.39 (2H, d, *J* = 8.0 Hz), 7.14 (1H, d, *J* = 1.0 Hz, H-3′), 5.81 (1H, d, *J* = 1.0 Hz, H-5′), 5.34 (1H, d, *J* = 11.5 Hz), 5.25 (1H, d, *J* = 11.5 Hz), 3.74 (3H, s, H-7′), 3.67 (3H, s, H-9′), 2.58 (3H, s, H-7); ^13^C NMR (125 MHz, CDCl_3_) *δ* 190.7 (C-8), 185.2 (C-4′), 168.4 (C-6′), 167.1 (C-2), 163.6 (C-8′), 150.6 (C-4), 146.1 (C-6), 137.7 (C-3′), 137.5 (C-2′), 135.2 (C), 131.7 (CH×2), 130.5 (CH×2), 122.7 (C), 122.3 (C-5), 113.8 (C-3), 113.4 (C-1), 104.4 (C-5′), 84.3 (C-1′), 75.9 (CH_2_), 57.1 (C-7′), 53.2 (C-9′), 19.1 (C-7); HRESIMS *m*/*z* 566.9570 [M + H]^+^ (calcd for C_24_H_18_BrCl_2_O_7_^+^, 566.9607).

##### Methyl(R)-5, 7-dichloro-4-((2-chlorobenzyl) oxy)-6′-methoxy-6-methyl-3, 4′-dioxo-3H-spiro [benzofuran-2, 1′-cyclohexane]-2′, 5′-diene-2′-carboxylate (**5**)

White, solid; yield 30.0%; ^1^H NMR (500 MHz, CDCl_3_) *δ* 7.75 (1H, dd, *J* = 7.5, 2.0 Hz), 7.38 (1H, dd, *J* = 7.5, 2.0 Hz), 7.29 (2H, ddd, *J* = 7.5, 2.0 Hz), 7.14 (1H, d, *J* = 1.0 Hz, H-3′), 5.82 (1H, d, *J* = 1.0 Hz, H-5′), 5.48 (1H, d, *J* = 12.0 Hz), 5.41 (1H, d, *J* = 12.0 Hz), 3.75 (3H, s, H-7′), 3.70 (3H, s, H-9′), 2.59 (3H, s, H-7); ^13^C NMR (CDCl_3_, 125 MHz) *δ* 190.5 (C-8), 185.3 (C-4′), 168.4 (C-6′), 167.0 (C-2), 163.6 (C-8′), 151.1 (C-4), 146.1 (C-6), 137.8 (C-3′), 137.6 (C-2′), 134.2 (C), 133.5 (CH), 130.4 (CH), 129.7 (CH), 129.5 (CH), 127.1 (C), 122.2 (C-5), 113.6 (C-3), 113.3 (C-1), 104.5 (C-5′), 84.2 (C-1′), 74.1 (CH_2_), 57.1 (C-7′), 53.2 (C-9′), 19.1 (C-7); HRESIMS *m*/*z* 523.0117 [M + H]^+^ (calcd for C_24_H_18_Cl_3_O_7_^+^, 523.0113).

##### Methyl(R)-5, 7-dichloro-4-((3-chlorobenzyl) oxy)-6′-methoxy-6-methyl-3, 4′-dioxo-3H-spiro [benzofuran-2, 1′-cyclohexane]-2′, 5′-diene-2′-carboxylate (**6**)

White, solid; yield 92.3%; ^1^H NMR (500 MHz, CDCl_3_) *δ* 7.52 (1H, s), 7.43 (1H, m), 7.30 (2H, overlapped), 7.14 (1H, d, *J* = 1.3 Hz, H-3′), 5.82 (1H, d, *J* = 1.3 Hz, H-5′), 5.35 (1H, d, *J* = 11.3 Hz), 5.26 (1H, d, *J* = 11.3 Hz), 3.75 (3H, s, H-7′), 3.69 (3H, s, H-9′), 2.58 (3H, s, H-7); ^13^C NMR (125 MHz, CDCl_3_) *δ* 190.7 (C-8), 185.2 (C-4′), 168.4 (C-6′), 167.1 (C-2), 163.6 (C-8′), 150.6 (C-4), 146.1 (C-6), 138.2 (C-3′), 137.7 (C-2′), 137.5 (C), 134.4 (C), 129.9 (CH), 128.7 (CH×2), 126.7 (CH), 122.2 (C-5), 113.8 (C-3), 113.5 (C-1), 104.5 (C-5′), 84.3 (C-1′), 75.8 (CH_2_), 57.1 (C-7′), 53.2(C-9′), 19.1(C-7); HRESIMS *m*/*z* 523.0120 [M + H]^+^ (calcd for C_24_H_18_Cl_3_O_7_^+^, 523.0113).

##### Methyl(R)-5,7-dichloro-4-((4-chlorobenzyl)oxy)-6′-methoxy-6-methyl-3,4′-dioxo-3H-spiro[benzofuran-2,1′-cyclohexane]-2′,5′-diene-2′-carboxylate (**7**)

White, solid; yield 50.0%; ^1^H NMR (500 MHz, CDCl_3_) *δ* 7.46 (2H, d, *J* = 8.15 Hz), 7.33 (2H, d, *J* = 8.1 Hz), 7.14 (1H, s, H-3′), 5.81 (1H, s, H-5′), 5.34 (1H, d, *J* = 11.1 Hz), 5.26 (1H, d, *J* = 11.1 Hz), 3.74 (3H, s, H-7′), 3.68 (3H, s, H-9′), 2.58 (3H, s, H-7); ^13^C NMR (125 MHz, CDCl_3_) *δ* 190.7 (C-8), 185.2 (C-4′), 168.4 (C-6′), 167.1 (C-2), 163.7 (C-8′), 150.7 (C-4), 146.1 (C-6), 137.8 (C-3′), 137.5 (C-2′), 134.7 (C), 134.5 (CH×2), 130.2 (CH×2), 128.8 (C), 122.3 (C-5), 113.8 (C-3), 113.5 (C-1), 104.5 (C-5′), 84.3 (C-1′), 75.9 (CH_2_), 57.1 (C-7′), 53.2 (C-9′), 19.1 (C-7); HRESIMS *m*/*z* 523.0120 [M + H]^+^ (calcd for C_24_H_18_Cl_3_O_7_^+^, 523.0113).

##### Methyl(R)-5, 7-dichloro-4-((2, 6-dichlorobenzyl) oxy)-6′-methoxy-6-methyl-3, 4′-dioxo-3H-spiro [benzofuran-2, 1′-cyclohexane]-2′, 5′-diene-2′-carboxylate (**8**)

White, solid; yield 71.5%; ^1^H NMR (500 MHz, CDCl_3_) *δ* 7.34 (1H, d, *J* = 0.5 Hz), 7.32 (1H, s), 7.23 (1H, ddd, *J* = 7.4, 1.4 Hz), 7.14 (1H, d, *J* = 1.4 Hz, H-3′), 5.81 (1H, d, *J* = 1.4 Hz, H-5′), 5.73 (1H, d, *J* = 11.5 Hz), 5.66 (1H, d, *J* = 11.5 Hz), 3.73 (3H, s, H-7′), 3.69 (3H, s, H-9′), 2.55 (3H, s, H-7); ^13^C NMR (125 MHz, CDCl_3_) *δ* 190.3 (C-8), 185.3 (C-4′), 168.5 (C-6′), 166.9 (C-2), 163.5 (C-8′), 151.6 (C-4), 145.9 (C-6), 137.8 (C-3′), 137.5 (C-2′), 137.3 (C), 132.2 (CH), 130.8 (C×2), 128.5 (CH×2), 121.8 (C-5), 113.2 (C-3), 112.9 (C-1), 104.4 (C-5′), 84.3 (C-1′), 72.0 (CH_2_), 57.0 (C-7′), 53.1 (C-9′), 19.1 (C-7); HRESIMS *m*/*z* 556.9730 [M + H]^+^ (calcd for C_24_H_17_Cl_4_O_7_^+^, 556.9723).

##### Methyl(R)-5, 7-dichloro-4-((3, 4-dichlorobenzyl) oxy)-6′-methoxy-6-methyl-3, 4′-dioxo-3H-spiro [benzofuran-2, 1′-cyclohexane]-2′, 5′-diene-2′-carboxylate (**9**)

White, solid; yield 94.3%; ^1^H NMR (500 MHz, CDCl_3_) *δ* 7.61 (1H, d, *J* = 1.0 Hz), 7.44 (1H, d, *J* = 8.0 Hz), 7.38 (1H, d, *J* = 8.0 Hz), 7.14 (1H, d, *J* = 1.0 Hz, H-3′), 5.82 (1H, d, *J* = 1.0 Hz, H-5′), 5.33 (1H, d, *J* = 11.5 Hz), 5.25 (1H, d, *J* = 11.5 Hz), 3.75 (3H, s, H-7′), 3.69 (3H, s, H-9′), 2.58 (3H, s, H-7); ^13^C NMR (125 MHz, CDCl_3_) *δ* 190.8 (C-8), 185.2 (C-4′), 168.3 (C-6′), 167.1 (C-2), 163.7 (C-8′), 150.3 (C-4), 146.2 (C-6), 137.7 (C-3′), 137.5 (C-2′), 136.4 (C), 132.7 (C), 132.6 (CH), 130.6 (CH×2), 128.0 (C), 122.2 (C-5), 113.8 (C-3), 113.7 (C-1), 104.5 (C-5′), 84.3 (C-1′), 75.1 (CH_2_), 57.1 (C-7′), 53.2 (C-9′), 19.1 (C-7); HRESIMS *m*/*z* 556.9730 [M + H]^+^ (calcd for C_24_H_17_Cl_4_ O_7_^+^, 556.9723).

##### Methyl(R)-5, 7-dichloro-4-((3-iodobenzyl) oxy)-6′-methoxy-6-methyl-3, 4′-dioxo-3H-spiro [benzofuran-2, 1′-cyclohexane]-2′, 5′-diene-2′-carboxylate (**10**)

White, solid; yield 94.1%; ^1^H NMR (500 MHz, CDCl_3_) *δ* 7.88 (1H, s), 7.66 (1H, d, *J* = 7.8 Hz), 7.52 (1H, d, *J* = 7.8 Hz), 7.14 (1H, d, *J* = 7.8 Hz), 7.10 (1H, t, 7.8 Hz, H-3′), 5.82 (1H, d, *J* = 1.5 Hz, H-5′), 5.30 (1H, d, *J* = 11.2 Hz), 5.21 (1H, d, *J* = 11.2 Hz), 3.75 (3H, s, H-7′), 3.69 (3H, s, H-9′), 2.58 (3H, s, H-7); ^13^C NMR (125 MHz, CDCl_3_) *δ* 190.7 (C-8), 185.2 (C-4′), 168.4 (C-6′), 167.0 (C-2), 163.6 (C-8′), 150.6 (C-4), 146.1 (C-6), 138.5 (C-3′), 137.7 (C-2′), 137.6 (C), 137.5 (CH×2), 130.3 (CH), 127.8 (CH), 122.2 (C-5), 113.8 (C-3), 113.5 (C-1), 104.5 (C-5′), 94.3 (C), 84.3 (C-1′), 75.7 (CH_2_), 57.1 (C-7′), 53.2 (C-9′), 19.1 (C-7); HRESIMS *m/z* 614.9468 [M + H]^+^ (calcd for C_24_H_18_Cl_2_IO_7_^+^, 614.9469).

##### Methyl(R)-5, 7-dichloro-4-((4-iodobenzyl) oxy)-6′-methoxy-6-methyl-3, 4′-dioxo-3H-spiro [benzofuran-2, 1′-cyclohexane]-2′, 5′-diene-2′-carboxylate (**11**)

White, solid; yield 93.7%; ^1^H NMR (500 MHz, CDCl_3_) *δ* 7.67 (2H, d, *J* = 8.3 Hz), 7.26 (2H, d, *J* = 8.3 Hz), 7.13 (1H, d, *J* = 1.4 Hz, H-3′), 5.81 (1H, d, *J* = 1.4 Hz, H-5′), 5.33 (1H, d, *J* = 11.3 Hz), 5.24 (1H, d, *J* = 11.3 Hz), 3.74 (3H, s, H-7′), 3.67 (3H, s, H-9′), 2.58 (3H, s, H-7); ^13^C NMR (125 MHz, CDCl_3_) *δ*190.7 (C-8), 185.2 (C-4′), 168.3 (C-6′), 167.0 (C-2), 163.6 (C-8′), 150.6 (C-4), 146.1 (C-6), 137.7 (C-3′), 137.6 (CH×2), 137.5 (C-2′), 135.8 (C), 130.6 (CH×2), 122.2 (C-5), 113.7 (C-3), 113.4 (C-1), 104.4 (C-5′), 94.5 (C), 84.2 (C-1′), 76.0 (CH_2_), 57.1 (C-7′), 53.2 (C-9′), 19.1 (C-7); HRESIMS *m*/*z* 614.9477 [M + H]^+^ (calcd for C_24_H_18_Cl_2_IO_7_^+^, 614.9469).

##### Methyl(R)-5, 7-dichloro-4-((2-fluorobenzyl) oxy)-6′-methoxy-6-methyl-3, 4′-dioxo-3H-spiro [benzofuran-2, 1′-cyclohexane]-2′, 5′-diene-2′-carboxylate (**12**)

White, solid; yield 58.4%; ^1^H NMR (500 MHz, CDCl_3_) *δ* 7.64 (1H, t, *J* = 7.5 Hz), 7.32 (1H, m, *J* = 7.5 Hz), 7.15 (2H, overlapped), 7.06 (1H, t, *J* = 9.5 Hz, H-3′), 5.82 (1H, d, *J* = 0.5 Hz, H-5′), 5.44 (1H, d, *J* = 11 Hz), 5.35 (1H, d, *J* = 11 Hz), 3.74 (3H, s, H-7′), 3.70 (3H, s, H-9′), 2.57 (3H, s, H-7); ^13^C NMR (125 MHz, CDCl_3_) *δ* 190.6 (C-8), 185.3 (C-4′), 168.5 (C-6′), 167.1 (C-2), 163.6 (C-8′), 162.0 (C), 150.9 (C-4), 146.1 (C-6), 137.8 (C-3′), 137.6 (C-2′), 131.3 (C), 130.5 (CH), 124.3 (CH×2), 122.4 (CH), 115.5 (C-5), 113.8 (C-3), 113.4 (C-1), 104.4 (C-5′), 84.3 (C-1′), 70.5 (CH_2_), 57.1 (C-7′), 53.2 (C-9′), 19.1 (C-7); HRESIMS *m*/*z* 507.0413 [M + H]^+^ (calcd for C_24_H_18_Cl_2_FO_7_^+^, 507.0408).

##### Methyl(R)-5, 7 -dichloro-4-((3, 5-difluorobenzyl) oxy)-6′-methoxy-6-methyl-3, 4′-dioxo-3H-spiro [benzofuran-2, 1′-cyclohexane]-2′, 5′-diene-2′-carboxylate (**13**)

White, solid; yield 81.1%; ^1^H NMR (500 MHz, CDCl_3_) *δ* 7.14 (1H, d, *J* = 1.4 Hz, H-3′), 7.05 (2H, overlapped), 6.76 (1H, dt, *J* = 2.2 Hz), 5.82 (1H, d, *J* = 1.4 Hz, H-5′), 5.37 (1H, d, *J* = 11.7 Hz), 5.28 (1H, d, *J* = 11.7 Hz), 3.75 (3H, s, H-7′), 3.69 (3H, s, H-9′), 2.58 (3H, s, H-7); ^13^C NMR (125 MHz, CDCl_3_) *δ* 190.8 (C-8), 185.2 (C-4′), 168.3 (C-6′), 167.1 (C-2), 163.7 (C-8′), 162.1(C), 150.2 (C-4), 146.3 (C-6), 140.0 (C-3′), 137.7 (C), 137.5 (C-2′), 122.1 (C), 111.3 (C-5), 111.1 (C-3), 110.1 (C-1), 104.5 (CH), 104.1(CH), 103.9 (CH), 103.7 (C-5′), 84.2 (C-1′), 75.1 (CH_2_), 57.1 (C-7′), 53.2 (C-9′), 19.1 (C-7); HRESIMS *m*/*z* 525.0316 [M + H]^+^ (calcd for C_24_H_17_Cl_2_F_2_O_7_^+^, 525.0314).

##### Methyl(R)-5, 7-dichloro-4-((3, 4-difluorobenzyl) oxy)-6′-methoxy-6-methyl-3, 4′-dioxo-3H-spiro [benzofuran-2, 1′-cyclohexane]-2′, 5′-diene-2′-carboxylate (**14**)

White, solid; yield 91.2%; ^1^H NMR (500 MHz, CDCl_3_) *δ* 7.37 (1H, dt, *J* = 2.0 Hz), 7.25 (1H, m, H-3′), 7.14 (2H, overlapped), 5.82 (1H, d, *J* = 1.5 Hz, H-5′), 5.32 (1H, d, *J* = 11.0 Hz), 5.23 (1H, d, *J* = 11.0 Hz), 3.75 (3H, s, H-7′), 3.69 (3H, s, H-9′), 2.58 (3H, s, H-7); ^13^C NMR (125 MHz, CDCl_3_) *δ* 190.8 (C-8), 185.2 (C-4′), 168.3 (C-6′), 167.1 (C-2), 163.7 (C-8′), 150.4 (C-4), 146.2 (C-6), 137.7 (C-3′), 137.5 (C-2′), 133.2 (C), 132.7 (C), 125.0 (C), 122.3 (CH), 118.1 (CH), 117.9 (CH), 117.4 (C), 117.3 (C-5), 113.9 (C-3), 113.7 (C-1), 104.5 (C-5′), 84.3 (C-1′), 75.3 (CH_2_), 57.1 (C-7′), 53.2 (C-9′), 19.1 (C-7); HRESIMS *m*/*z* 525.0325 [M + H]^+^ (calcd for C_24_H_17_Cl_2_F_2_O_7_^+^, 525.0314).

##### Methyl(R)-5, 7-dichloro-6′-methoxy-6-methyl-3, 4′-dioxo-4-((2, 3, 4, 5-tetrafluorobenzyl) oxy)-3H-spiro[benzofuran-2, 1′-cyclohexane]-2′, 5′-diene-2′-carboxylate (**15**)

White, solid; yield 25.0%; ^1^H NMR (500 MHz, CDCl_3_) *δ* 7.33 (1H, m), 7.14 (1H, d, *J* = 1.4 Hz, H-3′), 5.83 (1H, d, *J* = 1.4 Hz, H-5′), 5.39 (1H, d, *J* = 11.9 Hz), 5.31 (1H, d, *J* = 11.9 Hz), 3.76 (3H, s, H-7′), 3.71 (3H, s, H-9′), 2.58 (3H, s, H-7); ^13^C NMR (125 MHz, CDCl_3_) *δ* 190.8 (C-8), 185.2(C-4′), 168.2 (C-6′), 167.1 (C-2), 163.7 (C-8′), 149.9 (C-4), 146.3 (C-6), 137.6 (C-3′), 137.6 (C-2′), 137.6 (C), 122.3 (C), 119.9 (C), 116.2 (CH), 114.2 (C), 113.8 (C-5), 112.2 (C-3), 112.0 (C-1), 108.3 (C), 104.5 (C-5′), 84.3 (C-1′), 68.6 (CH_2_), 57.1 (C-7′), 53.2 (C-9′), 19.1 (C-7); HRESIMS *m*/*z* 561.0137 [M + H]^+^ (calcd for C_24_H_15_Cl_2_F_4_O_7_^+^, 561.0125).

##### Methyl(R)-5,7-dichloro-6′-methoxy-6-methyl-3, 4′-dioxo-4-((2, 3, 4-trifluorobenzyl)oxy)-3H-spiro [benzofuran-2, 1′-cyclohexane]-2′, 5′-diene-2′-carboxylate (**16**)

White, solid; yield 31.0%; ^1^H NMR (500 MHz, CDCl_3_) *δ* 7.37 (1H, m), 7.14 (1H, d, *J* = 1.4 Hz, H-3′), 6.97 (1H, m), 5.82 (1H, d, *J* = 1.4 Hz, H-5′), 5.40 (1H, d, *J* = 11.6 Hz), 5.30 (1H, d, *J* = 11.6 Hz), 3.75 (3H, s, H-7′), 3.70 (3H, s, H-9′), 2.57 (3H, s, H-7); ^13^C NMR (125 MHz, CDCl_3_) *δ* 190.8 (C-8), 185.2 (C-4′), 168.3 (C-6′), 167.1 (C-2), 163.7 (C-8′), 150.3 (C-4), 146.2 (C-6), 137.7 (C-3′), 137.5 (C-2′), 125.1 (C), 122.4 (C), 121.0 (C-5), 113.9 (C-3), 113.9 (C-1), 112.4 (CH), 112.3(C), 112.2(C), 112.2 (CH), 104.5 (C-5′), 84.3 (C-1′), 69.3 (CH_2_), 57.1 (C-7′), 53.2 (C-9′), 19.1(C-7); HRESIMS *m*/*z* 543.0213 [M + H]^+^ (calcd for C_24_H_16_Cl_2_F_3_O_7_^+^, 543.0220).

##### Methyl(R)-5,7-dichloro-6′-methoxy-6-methyl-3, 4′-dioxo-4-((perfluorophenyl) methoxy)-3H-spiro[benzofuran-2,1′-cyclohexane]-2′,5′-diene-2′-carboxylate (**17**)

White, solid; yield 20.0%; ^1^H NMR (500 MHz, CDCl_3_) *δ* 7.13 (1H, d, *J* = 1.2 Hz, H-3′), 5.81 (1H, d, *J* = 1.2 Hz, H-5′), 5.55 (1H, d, *J* = 11.8 Hz), 5.38 (1H, d, *J* = 11.8 Hz), 3.75 (3H, s, H-7′), 3.70 (3H, s, H-9′), 2.57 (3H, s, H-7); ^13^C NMR (125 MHz, CDCl_3_) *δ*190.7 (C-8), 185.2 (C-4′), 168.3 (C-6′), 167.1 (C-2), 163.6 (C-8′), 149.8 (C-4), 146.2 (C-6), 137.6 (C-3′), 137.6 (C-2′), 135.0 (C), 127.4 (C), 122.4 (C), 117.0 (C), 114.4 (C-5), 114.1 (C-3), 113.9 (C-1), 109.7 (C), 104.5 (C-5′), 84.3 (C-1′), 63.1 (CH_2_), 57.1 (C-7′), 53.2 (C-9′), 19.1 (C-7); HRESIMS *m*/*z* 579.0034 [M + H]^+^ (calcd for C_24_H_14_Cl_2_F_5_O_7_^+^, 579.0031).

##### Methyl(R)-5, 7-dichloro-6′-methoxy-6-methyl-3, 4′-dioxo-4-((2-(trifluoromethyl) benzyl) oxy)-3H-spiro[benzofuran-2,1′-cyclohexane]-2′, 5′-diene-2′-carboxylate (**18**)

White, solid; yield 50.0%; ^1^H NMR (500 MHz, CDCl_3_) *δ* 8.06 (1H, d, *J* = 7.8 Hz), 7.66 (1H, d, *J* = 7.8 Hz), 7.62 (1H, t, *J* = 7.6 Hz), 7.44 (1H, t, *J* = 7.6 Hz), 7.14 (1H, d, *J* = 1.5 Hz, H-3′), 5.82 (1H, d, *J* = 1.4 Hz, H-5′), 5.48 (2H, q, *J* = 12.5 Hz), 3.74 (3H, s, H-7′), 3.70 (3H, s, H-9′), 2.60 (3H, s, H-7); ^13^C NMR (125 MHz, CDCl_3_) *δ* 190.6 (C-8), 185.3 (C-4′), 168.4 (C-6′), 167.0 (C-2), 163.6 (C-8′), 151.0 (C-4), 146.1 (C-6), 137.7 (C-3′), 137.6 (C-2′), 135.0 (C), 132.3 (CH), 130.1 (CH), 128.2 (CH), 125.8 (CH), 125.7 (C), 125.4 (C), 122.1 (C-5), 113.7 (C-3), 113.6 (C-1), 104.5 (C-5′), 84.2 (C-1′), 72.9 (CH_2_), 57.1 (C-7′), 53.2 (C-9′), 19.1 (C-7); HRESIMS *m*/*z* 557.0386 [M + H]^+^ (calcd for C_25_H_18_Cl_2_F_3_O_7_^+^, 557.0376).

##### Methyl(R)-5,7-dichloro-6′-methoxy-6-methyl-3,4′-dioxo-4-((3-(trifluoromethyl)benzyl) oxy)-3H-spiro[benzofuran-2, 1′-cyclohexane]-2′, 5′-diene-2′-carboxylate (**19**)

White, solid; yield 30.0%; ^1^H NMR (500 MHz, CDCl_3_) *δ* 7.80 (1H, s), 7.76 (1H, d, *J* = 7.7 Hz), 7.60 (1H, d, *J* = 7.7 Hz), 7.50 (1H, t, *J* = 7.7 Hz), 7.14 (1H, d, *J* = 1.3 Hz, H-3′), 5.82 (1H, d, *J* = 1.3 Hz, H-5′), 5.40 (1H, d, *J* = 11.4 Hz), 5.33 (1H, d, *J* = 11.4 Hz), 3.75 (3H, s, H-7′), 3.69 (3H, s, H-9′), 2.59 (3H, s, H-7); ^13^C NMR (125 MHz, CDCl_3_) *δ* 190.8 (C-8), 185.2 (C-4′), 168.4 (C-6′), 167.1 (C-2), 163.7 (C-8′), 150.6 (C-4), 146.2 (C-6), 137.8 (C-3′), 137.5 (C-2′), 137.2 (C), 131.9 (C), 129.1 (CH), 125.4 (CH×3), 122.3 (C-5), 113.8 (C-3), 113.7 (C-1), 104.5 (C-5′), 84.3 (C-1′), 75.9 (CH_2_), 57.1 (C-7′), 53.2 (C-9′), 19.1 (C-7); HRESIMS *m*/*z* 557.0380 [M + H]^+^ (calcd for C_25_H_18_Cl_2_F_3_O_7_^+^, 557.0376).

##### Methyl(R)-5,7-dichloro-6′-methoxy-6-methyl-3, 4′-dioxo-4-((4-(trifluoromethyl) benzyl) oxy)-3H-spiro[benzofuran-2, 1′-cyclohexane]-2′, 5′-diene-2′-carboxylate (**20**)

White, solid; yield 72.7%; ^1^H NMR (500 MHz, CDCl_3_) *δ* 7.66 (2H, d, *J* = 8.3 Hz), 7.62 (2H, d, *J* = 8.3 Hz), 7.14 (1H, d, *J* = 1.3 Hz, H-3′), 5.82 (1H, d, *J* = 1.3 Hz, H-5′), 5.44 (1H, d, *J* = 11.5 Hz), 5.35 (1H, d, *J* = 11.5 Hz), 3.75 (3H, s, H-7′), 3.68 (3H, s, H-9′), 2.59 (3H, s, H-7); ^13^C NMR (125 MHz, CDCl_3_) *δ* 190.8 (C-8), 185.2 (C-4′), 168.3 (C-6′), 167.1 (C-2), 163.7 (C-8′), 150.6 (C-4), 146.2 (C-6), 140.2 (C), 137.7 (C-3′), 137.5 (C-2′), 130.7 (CH), 130.5 (CH), 128.6 (CH×2), 125.5 (C), 122.1 (C-5), 113.7 (C-3), 113.6 (C-1), 104.5 (C-5′), 84.3 (C-1′), 75.7 (CH_2_), 57.1 (C-7′), 53.2 (C-9′), 19.1 (C-7); HRESIMS *m*/*z* 557.0380 [M + H]^+^ (calcd for C_25_H_18_Cl_2_F_3_O_7_^+^, 557.0376).

##### Methyl(R)-4-((3, 5-bis(trifluoromethyl)benzyl)oxy)-5, 7-dichloro-6′-methoxy-6-methyl-3, 4′-dioxo-3H-spiro[benzofuran-2, 1′-cyclohexane]-2′, 5′-diene-2′-carboxylate (**21**)

White, solid; yield 66.3%; ^1^H NMR (500 MHz, CDCl_3_) *δ* 8.03 (2H, s), 7.85 (1H, s), 7.14 (1H, d, *J* = 1.3 Hz, H-3′), 5.83 (1H, d, *J* = 1.3 Hz, H-5′), 5.45 (1H, d, *J* = 12.0 Hz), 5.40 (1H, d, *J* = 12.0 Hz), 3.76 (3H, s, H-7′), 3.70 (3H, s, H-9′), 2.59 (3H, s, H-7); ^13^C NMR (125 MHz, CDCl_3_) *δ* 190.9 (C-8), 185.2 (C-4′), 168.2 (C-6′), 167.1 (C-2), 163.8 (C-8′), 150.1 (C-4), 146.3 (C-6), 138.9 (C), 137.7 (C-3′), 137.5 (C-2′), 132.0 (C), 131.7 (CH), 128.5 (CH), 124.4 (CH), 122.45 (C), 122.43 (C), 122.3 (C), 122.1 (C-5), 114.1 (C-3), 113.8 (C-1), 104.6 (C-5′), 84.3 (C-1′), 74.9 (CH_2_), 57.1 (C-7′), 53.3 (C-9′), 19.1 (C-7); HRESIMS *m*/*z* 625.0253 [M + H]^+^ (calcd for C_26_H_17_Cl_2_F_6_O_7_^+^, 625.0250).

##### Methyl(R)-5,7-dichloro-6′-methoxy-6-methyl-4-((3-nitrobenzyl)oxy)-3,4′-dioxo-3H-spiro[benzofuran-2,1′-cyclohexane]-2′,5′-diene-2′-carboxylate (**22**)

White, solid; yield 50.0%; ^1^H NMR (500 MHz, CDCl_3_) *δ* 8.38 (1H, s), 8.19 (1H, d, *J* = 8.0 Hz), 7.92 (1H, d, *J* = 7.5 Hz), 7.56 (1H, t, *J* = 7.9 Hz), 7.13 (1H, d, *J* = 1.5 Hz, H-3′), 5.82 (1H, d, *J* = 1.5 Hz, H-5′), 5.40 (2H, q, *J* = 11.6 Hz), 3.76 (s, 3H, H-7′), 3.69 (s, 3H, H-9′), 2.58 (s, 3H, H-7); ^13^C NMR (125 MHz, CDCl_3_) *δ* 190.9 (C-8), 185.2 (C-4′), 168.3 (C-6′), 167.1 (C-2), 163.7 (C-8′), 150.2 (C-4), 148.4 (C-6), 146.3 (C), 138.3 (C), 137.7 (C-3′), 137.5 (C-2′), 134.6 (CH), 129.6 (CH), 123.5 (CH), 123.4 (CH), 122.2 (C-5), 113.9 (C-3), 113.8 (C-1), 110.1 (C), 104.5 (C-5′), 84.3 (C-1′), 75.3 (CH_2_), 57.1 (C-7′), 53.3 (C-9′), 19.1 (C-7); HRESIMS *m*/*z* 534.0367 [M + H]^+^ (calcd for C_24_H_18_Cl_2_NO_9_^+^, 534.0353).

##### Methyl(R)-5,7-dichloro-6′-methoxy-6-methyl-4-((2-methylbenzyl)oxy)-3,4′-dioxo-3H-spiro[benzofuran-2,1′-cyclohexane]-2′,5′-diene-2′-carboxylate (**23**)

White, solid; yield 50.0%; ^1^H NMR (500 MHz, CDCl_3_) *δ* 7.51 (1H, d, *J* = 7.50 Hz), 7.26 (1H, t, *J* = 6.2 Hz), 7.20 (2H, d, *J* = 8.5 Hz), 7.15 (1H, d, *J* = 1.5 Hz, H-3′), 5.82 (1H, d, *J* = 1.5 Hz, H-5′), 5.38 (1H, d, *J* = 10.8 Hz), 5.30 (1H, d, *J* = 10.8 Hz), 3.75 (3H, s, H-7′), 3.69 (3H, s, H-9′), 2.58 (3H, s, H-7), 2.49 (3H, s); ^13^C NMR (125 MHz, CDCl_3_) *δ* 190.6 (C-8), 185.3 (C-4′), 168.5 (C-6′), 167.0 (C-2), 163.6 (C-8′), 151.3 (C-4), 146.0 (C-6), 137.8 (C-2′), 137.7 (C-3′), 137.5 (C×2), 134.3 (C), 130.4 (C), 130.1 (C), 128.9 (C), 126.1 (C), 122.3 (C-5), 113.8 (C-3), 113.1 (C-1), 104.4 (C-5′), 84.2 (C-1′), 75.3 (CH_2_), 57.1 (C-7′), 53.2 (C-9′), 29.8 (CH_3_), 19.1 (C-7); HRESIMS *m*/*z* 503.0660 [M + H]^+^ (calcd for C_25_H_21_Cl_2_O_7_^+^, 503.0659).

##### Methyl(R)-5,7-dichloro-6′-methoxy-6-methyl-4-((3-methylbenzyl)oxy)-3,4′-dioxo-3H-spiro[benzofuran-2,1′-cyclohexane]-2′,5′-diene-2′-carboxylate (**24**)

White, solid; yield 50.0%; ^1^H NMR (500 MHz, CDCl_3_) *δ* 7.37 (1H, s), 7.33 (1H, d, *J* = 7.6 Hz), 7.26 (1H, q, *J* = 7.6 Hz), 7.15 (2H, Overlapped, H-3′), 5.82 (1H, d, *J* = 1.1 Hz, H-5′), 5.32 (1H, d, *J* = 10.7 Hz), 5.24 (1H, d, *J* = 10.7 Hz), 3.74 (3H, s, H-7′), 3.68 (3H, s, H-9′), 2.59 (3H, s, H-7), 2.36 (3H, s); ^13^C NMR (125 MHz, CDCl_3_) *δ* 190.6 (C-8), 185.3 (C-4′), 168.5 (C-6′), 167.0 (C-2), 163.6 (C-8′), 151.1 (C-4), 146.0 (C-6), 138.2 (C-2′), 137.8 (C-3′), 137.5 (C), 136.1 (C), 129.5 (CH), 129.4 (CH), 128.5 (CH), 125.8 (CH), 122.4 (C-5), 113.8 (C-3), 113.2 (C-1), 104.4 (C-5′), 84.2 (C-1′), 77.0 (CH_2_), 57.1 (C-7′), 53.2 (C-9′), 21.5 (CH_3_), 19.1 (C-7); HRESIMS *m*/*z* 503.0664 [M + H]^+^ (calcd for C_25_H_21_Cl_2_O_7_^+^, 503.0659).

##### Methyl(R)-5,7-dichloro-6′-methoxy-6-methyl-4-((4-methylbenzyl)oxy)-3,4′-dioxo-3H-spiro [benzofuran-2,1′-cyclohexane]-2′,5′-diene-2′-carboxylate (**25**)

White, solid; yield 50.0%; ^1^H NMR (500 MHz, CDCl_3_) *δ* 7.40 (2H, d, *J* = 7.9 Hz), 7.16 (2H, d, *J* = 7.8 Hz), 7.14 (1H, d, *J* = 1.2 Hz, H-3′), 5.81 (1H, d, *J* = 1.2 Hz, H-5′), 5.29 (2H, q, *J* = 10.7 Hz), 3.73 (3H, s, H-7′), 3.67 (3H, s, H-9′), 2.57 (3H, s, H-7), 2.34 (3H, s); ^13^C NMR (125 MHz, CDCl_3_) *δ* 190.6 (C-8), 185.3 (C-4′), 168.5 (C-6′), 167.0 (C-2), 163.6 (C-8′), 151.1 (C-4), 146.0 (C-6), 138.4 (C-2′), 137.8 (C-3′), 137.5 (C), 133.2 (C), 131.7 (CH), 130.5 (CH), 129.2 (CH), 128.9 (CH), 122.4 (C-5), 113.8 (C-3), 113.1 (C-1), 104.4 (C-5′), 84.3 (C-1′), 75.9 (CH_2_), 57.1(C-7′), 53.2 (C-9′), 21.4 (CH_3_), 19.1 (C-7); HRESIMS *m*/*z* 503.0663 [M + H]^+^ (calcd for C_25_H_21_Cl_2_O_7_^+^, 503.0659).

##### Methyl(R)-4-((4-(tert-butyl)benzyl) oxy)-5, 7-dichloro-6′-methoxy-6-methyl-3, 4′-dioxo-3H-spiro [benzofuran-2, 1′-cyclohexane]-2′, 5′-diene-2′-carboxylate (**26**)

White, solid; yield 42.0%; ^1^H NMR (500 MHz, CDCl_3_) *δ* 7.48 (2H, d, *J* = 8.3 Hz), 7.39 (2H, d, *J* = 8.3 Hz), 7.15 (1H, d, *J* = 1.5 Hz, H-3′), 5.82 (1H, d, *J* = 1.5 Hz, H-5′), 5.33 (1H, d, *J* = 10.7 Hz), 5.25 (1H, d, *J* = 10.7 Hz), 3.73 (3H, s, H-7′), 3.67 (3H, s, H-9′), 2.59 (3H, s, H-7), 1.32 (9H, overlapped); ^13^C NMR (125 MHz, CDCl_3_) *δ* 190.6 (C-8), 185.3 (C-4′), 168.5 (C-6′), 167.0 (C-2), 163.6 (C-8′), 151.6 (C-4), 151.2 (C), 146.0 (C-6), 137.8 (C-3′), 137.5 (C-2′), 133.3 (C), 128.5 (CH×2), 125.5 (CH×2), 122.4 (C-5), 113.8 (C-3), 113.1 (C-1), 104.4 (C-5′), 84.3 (C-1′), 76.8 (CH_2_), 57.1 (C-7′), 53.2 (C-9′), 34.7(C), 31.4 (CH_3_×3), 19.1 (C-7); HRESIMS *m*/*z* 545.1131[M + H]^+^ (calcd for C_28_H_27_ Cl_2_O_7_^+^, 545.1128).

##### Methyl(R)-4-([1,1′-biphenyl]-4-ylmethoxy)-5,7-dichloro-6′-methoxy-6-methyl-3,4′-dioxo-3H-spiro[benzofuran-2,1′-cyclohexane]-2′,5′-diene-2′-carboxylate (**27**)

White, solid; yield 81.0%; ^1^H NMR (500 MHz, CDCl_3_) *δ* 7.61 (6H, overlapped), 7.44 (2H, t, *J* = 7.8 Hz), 7.35 (1H, dt, *J* = 7.3, 1.2 Hz), 7.16 (1H, d, *J* = 1.4 Hz, H-3′), 5.82 (1H, d, *J* = 1.4 Hz, H-5′), 5.43 (1H, d, *J* = 10.9 Hz), 5.34 (1H, d, *J* = 10.9 Hz), 3.74 (3H, s, H-7′), 3.67 (3H, s, H-9′), 2.60 (3H, s, H-7); ^13^C NMR (125 MHz, CDCl_3_) *δ* 190.6 (C-8), 185.2 (C-4′), 168.4 (C-6′), 167.1 (C-2), 163.6 (C-8′), 151.0 (C-4), 146.0 (C-6), 141.5 (C), 140.8 (C), 137.8 (C-3′), 137.5 (C-2′), 135.2 (C), 129.2 (CH×2), 128.9 (CH×2), 127.5 (CH), 127.3 (CH×2), 127.2 (CH×2), 122.4 (C-5), 113.8 (C-3), 113.3 (C-1), 104.4 (C-5′), 84.3 (C-1′), 76.6 (CH_2_), 57.1 (C-7′), 53.2 (C-9′), 19.1 (C-7); HRESIMS *m*/*z* 565.0816 [M + H]^+^ (calcd for C_30_H_23_Cl_2_O_7_^+^, 565.0815).

##### Methyl(R)-5,7-dichloro-4-((2′-cyano-[1,1′-biphenyl]-4-yl)methoxy)-6′-methoxy-6-methyl-3, 4′-dioxo-3H-spiro [benzofuran-2, 1′-cyclohexane]-2′, 5′-diene-2′-carboxylate (**28**)

White, solid; yield 32.0%; ^1^H NMR (500 MHz, CDCl_3_) *δ* 7.76 (1H, dd, *J* = 7.7, 1.0 Hz), 7.69 (2H, d, *J* = 8.1 Hz), 7.65 (1H, ddd, *J* = 7.7, 1.0 Hz), 7.58 (2H, d, *J* = 8.2 Hz), 7.51 (1H, d, *J* = 7.3 Hz), 7.45 (1H, ddd, *J* = 7.6, 1.0 Hz), 7.15 (1H, d, *J* = 1.2 Hz, H-3′), 5.83 (1H, d, *J* = 1.2 Hz, H-5′), 5.43 (2H, q, *J* = 11.1 Hz), 5.34 (2H, q, *J* = 11.1 Hz), 3.75 (s, 3H, H-7′), 3.70 (s, 3H, H-9′), 2.60 (s, 3H, H-7); ^13^C NMR (125 MHz, CDCl_3_) *δ* 190.7 (C-8), 185.3 (C-4′), 168.5 (C-6′), 167.1 (C-2), 163.7 (C-8′), 151.0 (C-4), 146.2 (C-6), 145.2 (C), 138.4 (C), 137.8 (C-3′), 137.6 (C-2′), 136.9 (C), 133.9 (C), 133.0 (CH), 130.2 (CH), 129.0 (CH×2), 129.0 (CH×2), 127.8 (CH), 122.4 (CH), 118.8 (C-5), 113.8 (C-3), 113.4 (C-1), 111.4 (C), 104.5 (C-5′), 84.3 (C-1′), 76.4 (CH_2_), 57.1 (C-7′), 53.2 (C-9′), 19.1 (C-7); HRESIMS *m*/*z* 590.0770 [M + H]^+^ (calcd for C_31_H_22_Cl_2_NO_7_^+^, 590.0768).

##### Methyl(R)-5, 7-dichloro-4-((2-cyanobenzyl) oxy)-6′-methoxy-6-methyl-3, 4′-dioxo-3H-spiro [benzofuran-2, 1′-cyclohexane]-2′, 5′-diene-2′-carboxylate (**29**)

White, solid; yield 87.7%; ^1^H NMR (500 MHz, CDCl_3_) *δ* 7.90 (1H, d, *J* = 7.5 Hz), 7.68 (1H, dd, *J* = 7.5, 1.0 Hz), 7.64 (1H, ddd, *J* = 7.5, 1.0 Hz), 7.44 (1H, ddd, *J* = 7.5, 1.0 Hz), 7.14 (1H, d, *J* = 1.0 Hz, H-3′), 5.82 (1H, d, *J* = 1.5 Hz, H-5′), 5.47 (2H, q, *J* = 12.0 Hz), 3.75 (3H, s, H-7′), 3.70 (3H, s, H-9′), 2.59 (3H, s, H-7); ^13^C NMR (125 MHz, CDCl_3_) *δ* 190.7 (C-8), 185.2 (C-4′), 168.3 (C-6′), 167.1 (C-2), 163.6 (C-8′), 150.5 (C-4), 146.2 (C-6), 139.6 (C), 137.6 (C-3′), 137.5 (C-2′), 133.1 (C), 132.8 (CH), 129.9 (CH), 129.0 (CH), 122.4 (CH), 117.1 (C-5), 113.9 (C-3), 113.7 (C-9), 111.9 (C), 104.5 (C-5′), 84.3 (C-1′), 74.0 (CH_2_), 57.1 (C-7′), 53.2 (C-9′), 19.1 (C-7); HRESIMS *m*/*z* 514.0456 [M + H]^+^ (calcd for C_25_H_18_Cl_2_NO_7_^+^, 514.0455).

##### Methyl(R)-5, 7-dichloro-4-((4-cyanobenzyl) oxy)-6′-methoxy-6-methyl-3, 4′-dioxo-3H-spiro [benzofuran-2, 1′-cyclohexane]-2′, 5′-diene-2′-carboxylate (**30**)

White, solid; yield 85.3%; ^1^H NMR (500 MHz, CDCl_3_) *δ* 7.66 (4H, overlapped), 7.13 (1H, d, *J* = 1.3 Hz, H-3′), 5.82 (1H, d, *J* = 1.3 Hz, H-5′), 5.41 (1H, d, *J* = 11.9Hz), 5.33 (1H, d, *J* = 11.9Hz), 3.75 (3H, s, H-7′), 3.69 (3H, s, H-9′), 2.59 (3H, s, H-7); ^13^C NMR (125 MHz, CDCl_3_) *δ* 190.8 (C-8), 185.1 (C-4′), 168.2 (C-6′), 167.1 (C-2), 163.7 (C-8′), 150.3 (C-4), 146.3 (C-6), 141.5 (C), 137.7 (C-3′), 137.5 (C-2′), 132.4 (CH×2), 128.8 (CH×2), 122.1 (C), 118.8 (C-5), 113.8 (C-3), 113.7(C-1), 112.3 (C), 104.5 (C-5′), 84.3 (C-1′), 75.5 (CH_2_), 57.1 (C-7′), 53.2 (C-9′), 19.1 (C-7); HRESIMS *m*/*z* 514.0460 [M + H]^+^ (calcd for C_25_H_18_Cl_2_NO_7_^+^, 514.0455).

##### Methyl(R)-5,7-dichloro-4-((2-chloro-4-fluorobenzyl)oxy)-6′-methoxy-6-methyl-3, 4′-dioxo-3H-spiro [benzofuran-2, 1′-cyclohexane]-2′, 5′-diene-2′-carboxylate (**31**)

White, solid; yield 77.6%; ^1^H NMR (500 MHz, CDCl_3_) *δ* 7.72 (1H, dd, *J* = 6.2, 2.3 Hz), 7.14 (2H, overlapped), 7.01 (1H, ddd, *J* = 8.5, 2.4 Hz, H-3′), 5.82 (1H, d, *J* = 1.0 Hz, H-5′), 5.42 (1H, d, *J* = 12.0 Hz), 5.35(1H, d, *J* = 12.0 Hz), 3.75 (3H, s, H-7′), 3.70 (3H, s, H-9′), 2.58 (3H, s, H-7); ^13^C NMR (125 MHz, CDCl_3_) *δ* 190.7 (C-8) 185.2 (C-4′), 168.4 (C-6′), 167.0 (C-2), 163.7 (C-8′), 161.6(C), 150.8 (C-4), 146.1(C-6), 137.7(C-3′), 137.6 (C-2′), 132.0 (CH), 122.3 (C), 117.0 (CH), 116.8(C), 114.4 (C), 114.2 (C-5), 113.7 (C-3), 113.6 (C-1), 104.5 (C-5′), 73.4 (CH_2_), 57.1 (C-7′), 53.2 (C-9′), 19.1 (C-7); HRESIMS *m*/*z* 541.0029 [M + H]^+^ (calcd for C_24_H_17_Cl_3_FO_7_^+^, 541.0018).

##### Methyl(R)-5, 7-dichloro-4-((3-chloro-4-fluorobenzyl) oxy)-6′-methoxy-6-methyl-3, 4′-dioxo-3H-spiro [benzofuran-2, 1′-cyclohexane]-2′, 5′-diene-2′-carboxylate (**32**)

White, solid; yield 78.5%; ^1^H NMR (500 MHz, CDCl_3_) *δ* 7.58 (1H, dd, *J* = 7.0, 1.5 Hz), 7.42 (1H, m), 7.13 (2H, overlapped, H-3′), 5.82 (1H, d, *J* = 0.5 Hz, H-5′), 5.29 (1H, d, *J* = 11.0 Hz), 5.23 (1H, d, *J* = 11.0 Hz), 3.75 (3H, s, H-7′), 3.69 (3H, s, H-9′), 2.58 (3H, s, H-7); ^13^C NMR (125 MHz, CDCl_3_) *δ* 190.7 (C-8), 185.1 (C-4′), 168.2 (C-6′), 167.0 (C-2), 163.6 (C-8′), 157.1 (C), 150.2 (C-4), 146.1 (C-6), 137.6 (C-3′), 137.4 (C-2′), 133.1 (C), 128.6 (CH×2), 122.2 (CH), 116.7 (C), 116.5 (C-5), 113.7 (C-3), 113.6 (C-1), 104.4 (C-5′), 84.2 (C-1′), 75.1 (CH_2_), 57.0 (C-7′), 53.1 (C-9′), 19.0 (C-7); HRESIMS *m*/*z* 541.0031[M + H]^+^ (calcd for C_24_H_17_Cl_3_FO_7_^+^, 541.0018).

##### Methyl(R)-5, 7-dichloro-4-((3-chloro-2-fluorobenzyl) oxy)-6′-methoxy-6-methyl-3, 4′-dioxo-3H-spiro [benzofuran-2, 1′-cyclohexane]-2′, 5′-diene-2′-carboxylate (**33**)

White, solid; yield 94.5%; ^1^H NMR (500 MHz, CDCl_3_) *δ* 7.57 (1H, t, *J* = 6.5 Hz), 7.38 (1H, t, *J* = 7.5 Hz), 7.14 (1H, s, H-3′), 7.10 (1H, t, *J* = 8.0 Hz), 5.82 (1H, s, H-5′), 5.45 (1H, d, *J* = 11.5 Hz), 5.34 (1H, d, *J* = 11.5 Hz), 3.75 (3H, s, H-7′), 3.70 (3H, s, H-9′), 2.58 (3H, s, H-7); ^13^C NMR (CDCl_3_, 125 MHz) *δ* 190.7 (C-8), 185.2 (C-4′), 168.4 (C-6′), 167.1 (C-2), 163.6 (C-8′), 157.4 (C), 155.4 (C), 150.6 (C-4), 146.2 (C-6), 137.7 (C-3′), 137.5 (C-2′), 131.0 (C), 129.4 (CH×2), 124.8 (CH), 124.7 (CH), 122.4 (C-5), 113.8(C-3), 113.7 (C-1), 104.5 (C-5′), 84.3 (C-1′), 70.1 (CH_2_), 57.1 (C-7′), 53.2 (C-9′), 19.1 (C-7); HRESIMS *m*/*z* 541.0021[M + H]^+^ (calcd for C_24_H_17_Cl_3_FO_7_^+^, 541.0018).

##### Methyl(R)-5, 7-dichloro-4-((4-chloro-2-fluorobenzyl) oxy)-6′-methoxy-6-methyl-3, 4′-dioxo-3H-spiro [benzofuran-2, 1′-cyclohexane]-2′, 5′-diene-2′-carboxylate (**34**)

White, solid; yield 72.4%; ^1^H NMR (500 MHz, CDCl_3_) *δ* 7.58 (1H, t, *J* = 8.0 Hz), 7.15 (2H, dd, *J* = 9.5, 1.5 Hz), 7.10 (1H, dd, *J* = 9.5, 1.5 Hz, H-3′), 5.82 (1H, d, *J* = 1.2 Hz, H-5′), 5.39 (1H, d, *J* = 11.6 Hz), 5.30 (1H, d, *J* = 11.6 Hz), 3.75 (3H, s, H-7′), 3.69 (3H, s, H-9′), 2.57 (3H, s, H-7); ^13^C NMR (CDCl_3_, 125 MHz) *δ* 190.7 (C-8), 185.2 (C-4′), 168.4 (C-6′), 167.1 (C-2), 163.7 (C-8′), 150.5 (C-4), 146.1 (C-6), 137.7 (C-3′), 137.5 (C-2′), 132.1 (CH), 132.1 (C), 124.8 (CH), 124.7 (C),122.4 (C), 116.4 (CH), 116.2 (C-5), 113.8 (C-3), 113.7 (C-1), 104.5 (C-5′), 84.3 (C-1′), 69.7 (CH_2_), 57.1 (C-7′), 53.2 (C-9′), 19.1 (C-7); HRESIMS *m*/*z* 541.0023 [M + H]^+^ (calcd for C_24_H_17_Cl_3_FO_7_^+^, 541.0018).

##### Methyl(R)-4-((4-bromo-2-fluorobenzyl) oxy)-5,7-dichloro-6′-methoxy-6-methyl-3, 4′-dioxo-3H-spiro [benzofuran-2, 1′-cyclohexane]-2′, 5′-diene-2′-carboxylate (**35**)

White, solid; yield 52.0%; ^1^H NMR (500 MHz, CDCl_3_) *δ* 7.52 (1H, t, *J* = 8.0 Hz), 7.30 (1H, dd, *J* = 8.0, 1.5 Hz), 7.25 (1H, dd, *J* = 8.0, 2.0 Hz), 7.14 (1H, d, *J* = 1.5 Hz, H-3′), 5.82 (1H, d, *J* = 1.5 Hz, H-5′), 5.38 (1H, d, *J* = 12.0 Hz), 5.29 (1H, d, *J* = 12.0 Hz), 3.75 (3H, s, H-7′), 3.69 (3H, s, H-9′), 2.57 (3H, s, H-7); ^13^C NMR (125 MHz, CDCl_3_) *δ* 190.7 (C-8), 185.2 (C-4′), 168.3 (C-6′), 167.1 (C-2), 163.6 (C-8′), 161.7 (C), 159.7 (C), 150.5 (C-4), 146.1 (C-6), 137.7 (C-3′), 137.5 (C-2′), 132.3 (C), 127.7 (CH), 122.4 (CH), 119.3 (CH), 119.1 (C-5), 113.8 (C-3), 113.7 (C-1), 104.5 (C-5′) 84.3 (C-1′), 69.7 (CH_2_), 57.1 (C-7′), 53.2 (C-9′), 19.1 (C-7); HRESIMS *m*/*z* 584.9522 [M + H]^+^ (calcd for C_24_H_17_BrCl_2_FO_7_^+^, 584.9513).

##### Methyl(R)-5, 7-dichloro-4-((2-cyano-5-fluorobenzyl) oxy)-6′-methoxy-6-methyl-3, 4′-dioxo-3H-spiro [benzofuran-2, 1′-cyclohexane]-2′, 5′-diene-2′-carboxylate (**36**)

White solid; yield 60.0%; ^1^H NMR (500 MHz, CDCl_3_) *δ* 7.67 (2H, m), 7.14 (2H, ddd, *J* = 9.2, 1.3 Hz, H-3′), 5.83 (1H, d, *J* = 1.3 Hz, H-5′), 5.47 (2H, q, *J* = 12.8 Hz), 3.76 (3H, s, H-7′), 3.71 (3H, s, H-9′), 2.60 (3H, s, H-7); ^13^C NMR (125 MHz, CDCl_3_) *δ* 190.8 (C-8), 185.2 (C-4′), 168.2 (C-6′), 167.1 (C-2), 166.3 (C-8′), 163.7 (C), 150.1 (C-4), 146.3 (C-6), 137.6 (C-3′), 137.6 (C-2′), 135.1 (C), 135.1 (CH), 122.3 (CH), 117.2 (CH), 117.0 (C), 116.6 (C), 116.3 (C-5), 114.3 (C-3), 113.8 (C-1), 104.6 (C-5′), 84.3 (C-1′), 73.2 (CH_2_), 57.2 (C-7′), 53.3 (C-9′), 19.1 (C-7); HRESIMS *m*/*z* 532.0361 [M + H]^+^ (calcd for C_25_H_17_Cl_2_FNO_7_^+^, 532.0361).

##### Methyl(R)-5,7-dichloro-4-ethoxy-6′-methoxy-6-methyl-3,4′-dioxo3Hspiro[benzofuran-2,1′-cyclohexane]-2′,5′-diene-2′-carboxylate (**37**)

White, solid; yield 85.0%; ^1^H NMR (500 MHz, CDCl_3_) *δ* 7.12 (1H, d, *J* = 1.5 Hz, H-3′), 5.80 (1H, s, H-5′), 4.34 (2H, dq, *J* = 7.0 Hz), 3.73 (3H, s, H-7′), 3.68 (3H, s, H-9′), 2.58 (3H, s, H-7), 1.43 (3H, t, *J* = 7.0 Hz), ^13^C NMR (125 MHz, CDCl_3_) *δ* 190.4 (C-8), 185.3 (C-4′), 168.6 (C-6′), 167.0 (C-2), 163.6 (C-8′), 151.6 (C-4), 145.8 (C-6), 137.9 (C-3′), 137.5 (C-2′), 122.3 (C-5), 113.8 (C-3), 112.9 (C-1), 104.4 (C-5′), 84.3 (C-1′), 71.9 (CH_2_), 57.1 (C-7′), 53.2 (C-9′), 19.1 (C-7), 15.6 (CH_3_); HRESIMS *m*/*z* 427.0349 [M + H]^+^ (calcd for C_19_H_17_Cl_2_O_7_^+^, 427.0346).

### 3.5. Insecticidal Activity

The neonate larvae of *Helicoverpa armigera* Hübner were used to assess the insecticidal activity. The positive control was azadirachtin and DMSO was the negative control. Serial dilutions of the tested compounds were added alongside the positive control at 200, 100, 50, 25 and 12.5 μL/well with three replicates per treatment to the artificial diet for the newly hatched larvae while bioassay diet was placed into the six-well plates. New larvae were hatched at 25 °C and relative humidity of 80%. The number of dead larvae was recorded after treatments for 2, 4, 6 and 8 days [26].

### 3.6. Antibacterial Activity

The methods described by Fromtling et al. were used for the evaluation of antibacterial activities [27]. Ciprofloxacin and sea-nine 211 were used as positive controls. Bacterial species were cultured at 37 °C for 8 h in LB medium and diluted to 10^6^ cfu/mL, using 96-well microplates, having 2 μL test sample and 198 μL of bacterial solutions. The plates were incubated at 37 °C for 24 h while DMSO was used as the negative control.

## 4. Conclusions

In summary, 36 new derivatives of marine-derived geodin (**1**) were semisynthesized successfully via one mild step reaction with high yields. Among them, **15** showed significant insecticidal activity equivalent to the positive drug, azadirachtin. Meanwhile, **37** showed selective antibacterial activity against *P. aeruginosa*. The results revealed that modification of the 4-OH and introduction of halogen atoms, especially fluorine and chlorine atoms, could enhance the insecticidal and antibacterial activities of **1**. These findings bring further evidence that geodin derivatives are active and provide new information supporting the importance of continued studies into the structure–activity relationships of griseofulvin analogs.

## Figures and Tables

**Scheme 1:**
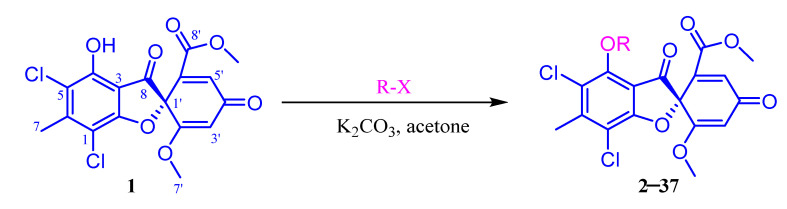
General semisynthetic strategy employed to make ether derivatives of **1**.

**Table 1:** Structures of compounds **2**–**37**.

No.	R	No.	R	No.	R
**2**	** 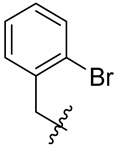 **	**14**	** 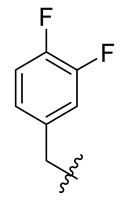 **	**26**	** 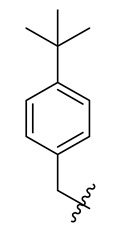 **
**3**	** 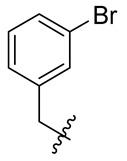 **	**15**	** 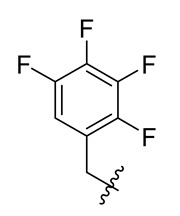 **	**27**	** 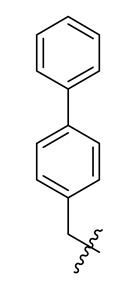 **
**4**	** 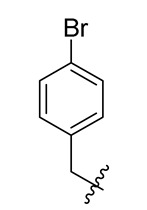 **	**16**	** 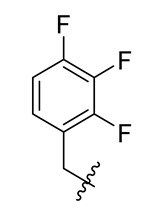 **	**28**	** 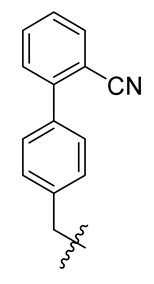 **
**5**	** 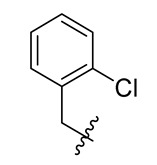 **	**17**	** 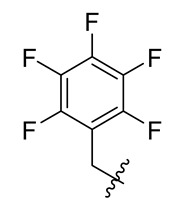 **	**29**	** 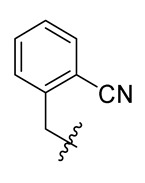 **
**6**	** 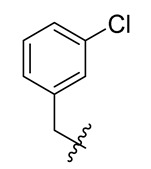 **	**18**	** 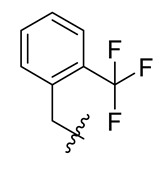 **	**30**	** 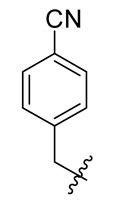 **
**7**	** 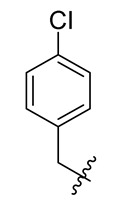 **	**19**	** 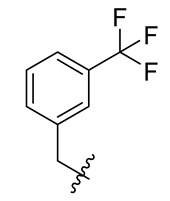 **	**31**	** 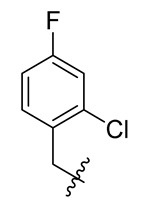 **
**8**	** 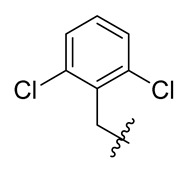 **	**20**	** 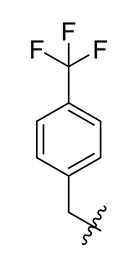 **	**32**	** 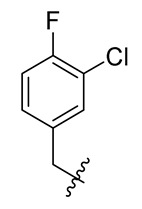 **
**9**	** 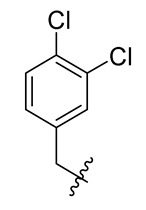 **	**21**	** 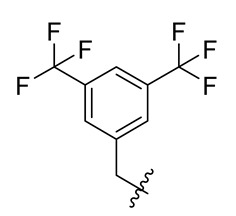 **	**33**	** 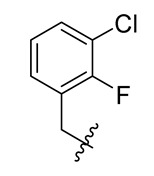 **
**10**	** 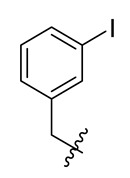 **	**22**	** 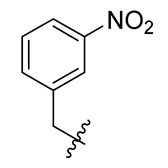 **	**34**	** 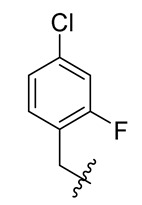 **
**11**	** 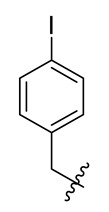 **	**23**	** 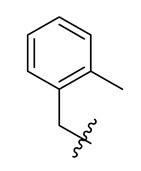 **	**35**	** 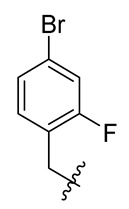 **
**12**	** 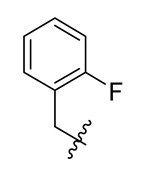 **	**24**	** 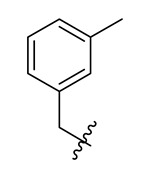 **	**36**	** 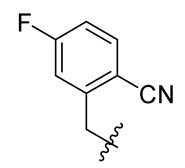 **
**13**	** 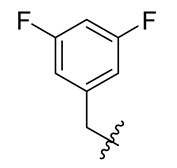 **	**25**	** 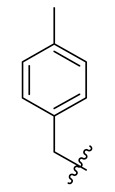 **	**37**	** 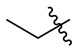 **

**Table 2:** Insecticidal and antibacterial activities of compounds **1**–**37** ^a^.

No.	IC_50_ (μM) (μg/mL)	MIC (μM)		
	Insecticidal Activity	Antimicrobial Activity		
	*H. armigera* Hübner	*S. aureus*	*A. salmonicida*	*P. aeruginosa*
**1**	500 (200)	>50	>50	>50
**2**	176 (100)	>50	2.20	>50
**3**	176 (100)	>50	>50	>50
**4**	>350 (>200)	17.60	8.80	>50
**5**	>381 (>200)	4.77	4.77	>50
**6**	190 (100)	9.55	4.77	>50
**7**	190 (100)	>50	>50	>50
**8**	179 (100)	8.96	4.48	>50
**9**	179 (100)	>50	4.48	>50
**10**	>325 (>200)	16.25	4.06	>50
**11**	>325 (>200)	>50	8.13	>50
**12**	197 (100)	4.93	2.46	>50
**13**	190 (100)	4.76	1.19	>50
**14**	190 (100)	9.52	2.38	>50
**15**	89 (50)	8.91	2.23	>50
**16**	>368 (>200)	4.60	1.15	>50
**17**	172 (100)	17.27	>50	>50
**18**	179 (100)	8.97	>50	>50
**19**	>358 (>200)	17.94	8.97	>50
**20**	179 (100)	17.94	4.49	>50
**21**	>319 (>200)	>50	4.00	>50
**22**	187 (100)	>50	>50	>50
**23**	198 (100)	>50	>50	>50
**24**	>397 (>200)	>50	>50	>50
**25**	nt	>50	>50	>50
**26**	366 (200)	>50	2.29	>50
**27**	176 (100)	>50	>50	>50
**28**	169 (100)	>50	>50	>50
**29**	>388 (>200)	9.72	>50	>50
**30**	194 (100)	4.86	4.86	>50
**31**	>369 (>200)	9.23	4.62	>50
**32**	184 (100)	9.23	9.23	>50
**33**	>369 (>200)	4.62	2.31	>50
**34**	369 (200)	9.23	>50	>50
**35**	170 (100)	>50	4.27	>50
**36**	>375 (>200)	18.97	>50	>50
**37**	>468 (>200)	>50	>50	5.85
Azadirachtin	70 (50)	nt	nt	nt
Sea-Nine 211	nt	nt	0.27	0.27
Ciprofloxacin	nt	0.16	nt	nt

nt = not tested. ^a^ Results are the average of three independent experiments, each performed in duplicate. Standard deviations were less than ±10%.

## Data Availability

Data are contained within the article or Supplementary Materials.

## Supplementary Materials

The following supporting information can be downloaded at: https://www.mdpi.com/article/10.3390/md20020082/s1, Figures S1–S112: ^1^H, ^13^C-NMR and HRESIMS data and charts of all compounds.
