# Supplementary material for: Design, Semisynthesis, Insecticidal and Antibacterial Activities of a Series of Marine-Derived Geodin Derivatives and Their Preliminary Structure–Activity Relationships
Source: Mar Drugs. 2022 Jan 18;20(2):82. doi: 10.3390/md20020082 (PMC8880215; doi:10.3390/md20020082)
Supplement: Supplementary file 1 [file marinedrugs-20-00082-s001.zip › marinedrugs-1552849-supplementary.pdf]

SUPPLEMENTARY MATERIAL

Design, Semisynthesis, Insecticidal and Antibacterial Activities of a Series of Marine-Derived Geodin Derivatives and Their Preliminary Structure-Activity Relationships

Rong Chao [1,†], Gulab Said [1,5,†], Qun Zhang [1], Yue-Xuan Qi [2], Jie Hu [1,2], Cai-Juan Zheng [3], Ji-Yong Zheng [2,*], Chang-Lun Shao [1,2], Guang-Ying Chen [3], and Mei-Yan Wei [1,4,*]

[1] Key Laboratory of Marine Drugs, The Ministry of Education of China, School of Medicine and Pharmacy, Ocean University of China, Qingdao 266003, China; chaorong1122@163.com (R.C.); gulabouc@gmail.com (G.S.); zhangqunnn@163.com (Q.Z.); hujiewrc@163.com (J.H.); shaochanglun@163.com (C.-L.S.); mywei95@126.com (M.-Y.W.)

[2] State Key Laboratory for Marine Corrosion and Protection, Luoyang Ship Material Research Institute (LSMRI), Qingdao 266061, China; qiyuexuan9475@163.com (Y.-X.Q.); zhengjy@sunrui.net (J.-Y.Z)

[3] Key Laboratory of Tropical Medicinal Resource Chemistry of Ministry of Education, Hainan Normal University, Haikou 570100, China; caijuan2002@163.com (C.-J.Z); chgying123@163.com (G.-Y.C)

[4] College of Food Science and Engineering, Ocean University of China, Qingdao 266003, People's Republic of China

[5] Department of Chemistry, Women University Swabi, Swabi 23430, Khyber Pakhtunkhwa, Pakistan.

* Correspondence: mywei95@126.com (M.-Y.W.); zhengjy@sunrui.net (J.-Y.Z); Tel: +86-532-8203-1381 (M.-Y.W.); +86-532-68725022 (J.-Y.Z)

† These authors contributed equally to this work.

Figure S1. ^{1}H NMR (500 MHz, $CDCl_3$) spectrum of compound **1**
Figure S2. ^{13}C NMR (125 MHz, $CDCl_3$) spectrum of compound **1**
Figure S3. ESIMS spectrum of compound **1**
Figure S4. ^{1}H NMR (500 MHz, $CDCl_3$) spectrum of compound **2**
Figure S5. ^{13}C NMR (125 MHz, $CDCl_3$) spectrum of compound **2**
Figure S6. HRESIMS spectrum of compound **2**
Figure S7. ^{1}H NMR (500 MHz, $CDCl_3$) spectrum of compound **3**
Figure S8. ^{13}C NMR (125 MHz, $CDCl_3$) spectrum of compound **3**
Figure S9. HRESIMS spectrum of compound **3**
Figure S10. ^{1}H NMR (500 MHz, $CDCl_3$) spectrum of compound **4**
Figure S11. ^{13}C NMR (125 MHz, $CDCl_3$) spectrum of compound **4**
Figure S12. HRESIMS spectrum of compound **4**
Figure S13. ^{1}H NMR (500 MHz, $CDCl_3$) spectrum of compound **5**
Figure S14. ^{13}C NMR (125 MHz, $CDCl_3$) spectrum of compound **5**
Figure S15. HRESIMS spectrum of compound **5**
Figure S16. ^{1}H NMR (500 MHz, $CDCl_3$) spectrum of compound **6**
Figure S17. ^{13}C NMR (125 MHz, $CDCl_3$) spectrum of compound **6**
Figure S18. HRESIMS spectrum of compound **6**
Figure S19. ^{1}H NMR (500 MHz, $CDCl_3$) spectrum of compound **7**
Figure S20. ^{13}C NMR (125 MHz, $CDCl_3$) spectrum of compound **7**
Figure S21. HRESIMS spectrum of compound **7**
Figure S22. ^{1}H NMR (500 MHz, $CDCl_3$) spectrum of compound **8**
Figure S23. ^{13}C NMR (125 MHz, $CDCl_3$) spectrum of compound **8**
Figure S24. HRESIMS spectrum of compound **8**
Figure S25. ^{1}H NMR (500 MHz, $CDCl_3$) spectrum of compound **9**
Figure S26. ^{13}C NMR (125 MHz, $CDCl_3$) spectrum of compound **9**
Figure S27. HRESIMS spectrum of compound **9**
Figure S28. ^{1}H NMR (500 MHz, $CDCl_3$) spectrum of compound **10**
Figure S29. ^{13}C NMR (125 MHz, $CDCl_3$) spectrum of compound **10**
Figure S30. HRESIMS spectrum of compound **10**
Figure S31. ^{1}H NMR (500 MHz, $CDCl_3$) spectrum of compound **11**
Figure S32. ^{13}C NMR (125 MHz, $CDCl_3$) spectrum of compound **11**
Figure S33. HRESIMS spectrum of compound **11**
Figure S34. ^{1}H NMR (500 MHz, $CDCl_3$) spectrum of compound **12**
Figure S35. ^{13}C NMR (125 MHz, $CDCl_3$) spectrum of compound **12**
Figure S36. HRESIMS spectrum of compound **12**
Figure S37. ^{1}H NMR (500 MHz, $CDCl_3$) spectrum of compound **13**
Figure S38. ^{13}C NMR (125 MHz, $CDCl_3$) spectrum of compound **13**
Figure S39. HRESIMS spectrum of compound **13**
Figure S40. ^{1}H NMR (500 MHz, $CDCl_3$) spectrum of compound **14**
Figure S41. ^{13}C NMR (125 MHz, $CDCl_3$) spectrum of compound **14**
Figure S42. HRESIMS spectrum of compound **14**
Figure S43. ^{1}H NMR (500 MHz, $CDCl_3$) spectrum of compound **15**
Figure S44. ^{13}C NMR (125 MHz, $CDCl_3$) spectrum of compound **15**
Figure S45. HRESIMS spectrum of compound **15**

Figure S46. ^{1}H NMR (500 MHz, $CDCl_3$) spectrum of compound **16**

Figure S47. ^{13}C NMR (125 MHz, $CDCl_3$) spectrum of compound **16**

Figure S48. HRESIMS spectrum of compound **16**

Figure S49. ^{1}H NMR (500 MHz, $CDCl_3$) spectrum of compound **17**

Figure S50. ^{13}C NMR (125 MHz, $CDCl_3$) spectrum of compound **17**

Figure S51. HRESIMS spectrum of compound **17**

Figure S52. ^{1}H NMR (500 MHz, $CDCl_3$) spectrum of compound **18**

Figure S53. ^{13}C NMR (125 MHz, $CDCl_3$) spectrum of compound **18**

Figure S54. HRESIMS spectrum of compound **18**

Figure S55. ^{1}H NMR (500 MHz, $CDCl_3$) spectrum of compound **19**

Figure S56. ^{13}C NMR (125 MHz, $CDCl_3$) spectrum of compound **19**

Figure S57. HRESIMS spectrum of compound **19**

Figure S58. ^{1}H NMR (500 MHz, $CDCl_3$) spectrum of compound **20**

Figure S59. ^{13}C NMR (125 MHz, $CDCl_3$) spectrum of compound **20**

Figure S60. HRESIMS spectrum of compound **20**

Figure S61. ^{1}H NMR (500 MHz, $CDCl_3$) spectrum of compound **21**

Figure S62. ^{13}C NMR (125 MHz, $CDCl_3$) spectrum of compound **21**

Figure S63. HRESIMS spectrum of compound **21**

Figure S64. ^{1}H NMR (500 MHz, $CDCl_3$) spectrum of compound **22**

Figure S65. ^{13}C NMR (125 MHz, $CDCl_3$) spectrum of compound **22**

Figure S66. HRESIMS spectrum of compound **22**

Figure S67. ^{1}H NMR (500 MHz, $CDCl_3$) spectrum of compound **23**

Figure S68. ^{13}C NMR (125 MHz, $CDCl_3$) spectrum of compound **23**

Figure S69. HRESIMS spectrum of compound **23**

Figure S70. ^{1}H NMR (500 MHz, $CDCl_3$) spectrum of compound **24**

Figure S71. ^{13}C NMR (125 MHz, $CDCl_3$) spectrum of compound **24**

Figure S72. HRESIMS spectrum of compound **24**

Figure S73. ^{1}H NMR (500 MHz, $CDCl_3$) spectrum of compound **25**

Figure S74. ^{13}C NMR (125 MHz, $CDCl_3$) spectrum of compound **25**

Figure S75. HRESIMS spectrum of compound **25**

Figure S76. ^{1}H NMR (500 MHz, $CDCl_3$) spectrum of compound **26**

Figure S77. ^{13}C NMR (125 MHz, $CDCl_3$) spectrum of compound **26**

Figure S78. HRESIMS spectrum of compound **26**

Figure S79. ^{1}H NMR (500 MHz, $CDCl_3$) spectrum of compound **27**

Figure S80. ^{13}C NMR (125 MHz, $CDCl_3$) spectrum of compound **27**

Figure S81. HRESIMS spectrum of compound **27**

Figure S82. ^{1}H NMR (500 MHz, $CDCl_3$) spectrum of compound **28**

Figure S83. ^{13}C NMR (125 MHz, $CDCl_3$) spectrum of compound **28**

Figure S84. HRESIMS spectrum of compound **28**

Figure S85. ^{1}H NMR (500 MHz, $CDCl_3$) spectrum of compound **29**

Figure S86. ^{13}C NMR (125 MHz, $CDCl_3$) spectrum of compound **29**

Figure S87. HRESIMS spectrum of compound **29**

Figure S88. ^{1}H NMR (500 MHz, $CDCl_3$) spectrum of compound **30**

Figure S89. ^{13}C NMR (125 MHz, $CDCl_3$) spectrum of compound **30**

Figure S90. HRESIMS spectrum of compound **30**
Figure S91. ^{1}H NMR (500 MHz, $CDCl_3$) spectrum of compound **31**
Figure S92. ^{13}C NMR (125 MHz, $CDCl_3$) spectrum of compound **31**
Figure S93. HRESIMS spectrum of compound **31**
Figure S94. ^{1}H NMR (500 MHz, $CDCl_3$) spectrum of compound **32**
Figure S95. ^{13}C NMR (125 MHz, $CDCl_3$) spectrum of compound **32**
Figure S96. HRESIMS spectrum of compound **32**
Figure S97. ^{1}H NMR (500 MHz, $CDCl_3$) spectrum of compound **33**
Figure S98. ^{13}C NMR (125 MHz, $CDCl_3$) spectrum of compound **33**
Figure S99. HRESIMS spectrum of compound **33**
Figure S100. ^{1}H NMR (500 MHz, $CDCl_3$) spectrum of compound **34**
Figure S101. ^{13}C NMR (125 MHz, $CDCl_3$) spectrum of compound **34**
Figure S102. HRESIMS spectrum of compound **34**
Figure S103. ^{1}H NMR (500 MHz, $CDCl_3$) spectrum of compound **35**
Figure S104. ^{13}C NMR (125 MHz, $CDCl_3$) spectrum of compound **35**
Figure S105. HRESIMS spectrum of compound **35**
Figure S106. ^{1}H NMR (500 MHz, $CDCl_3$) spectrum of compound **36**
Figure S107. ^{13}C NMR (125 MHz, $CDCl_3$) spectrum of compound **36**
Figure S108. HRESIMS spectrum of compound **36**
Figure S109. ^{1}H NMR (500 MHz, $CDCl_3$) spectrum of compound **37**
Figure S110. ^{13}C NMR (125 MHz, $CDCl_3$) spectrum of compound **37**
Figure S111. HRESIMS spectrum of compound **37**
Figure S112. Neighbor-joining phylogenetic tree

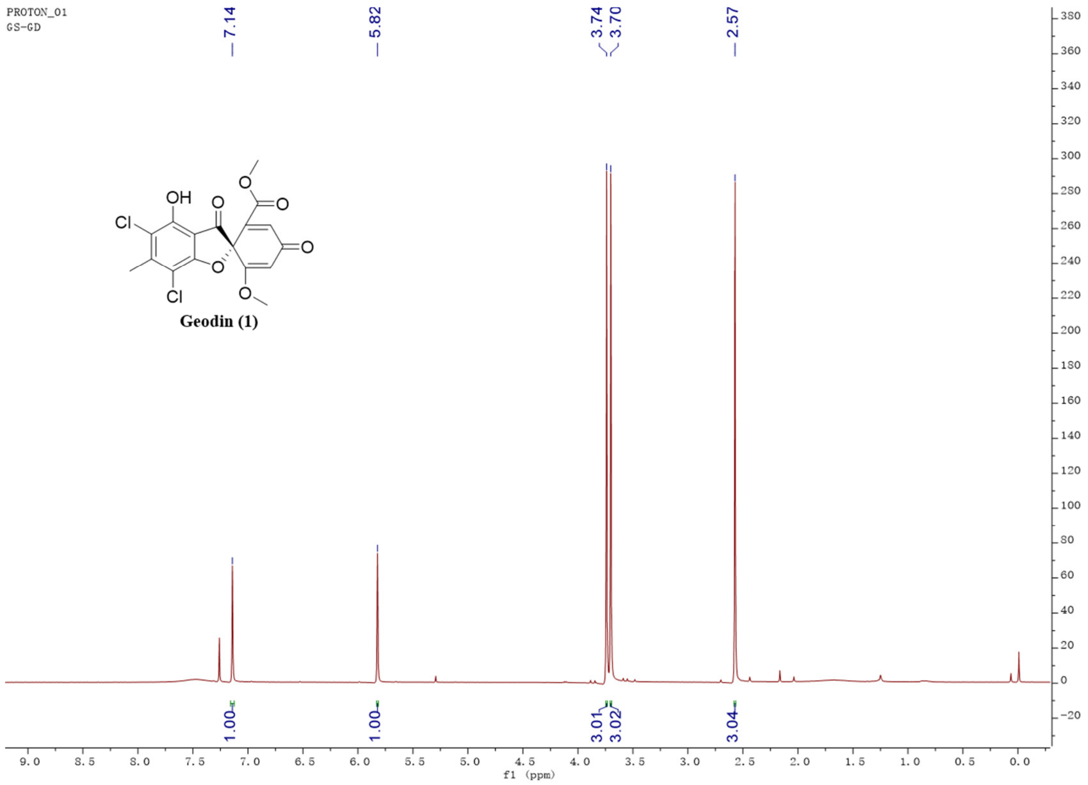

Figure S1. ^{1}H NMR (500 MHz, $CDCl_3$) spectrum of compound **1**

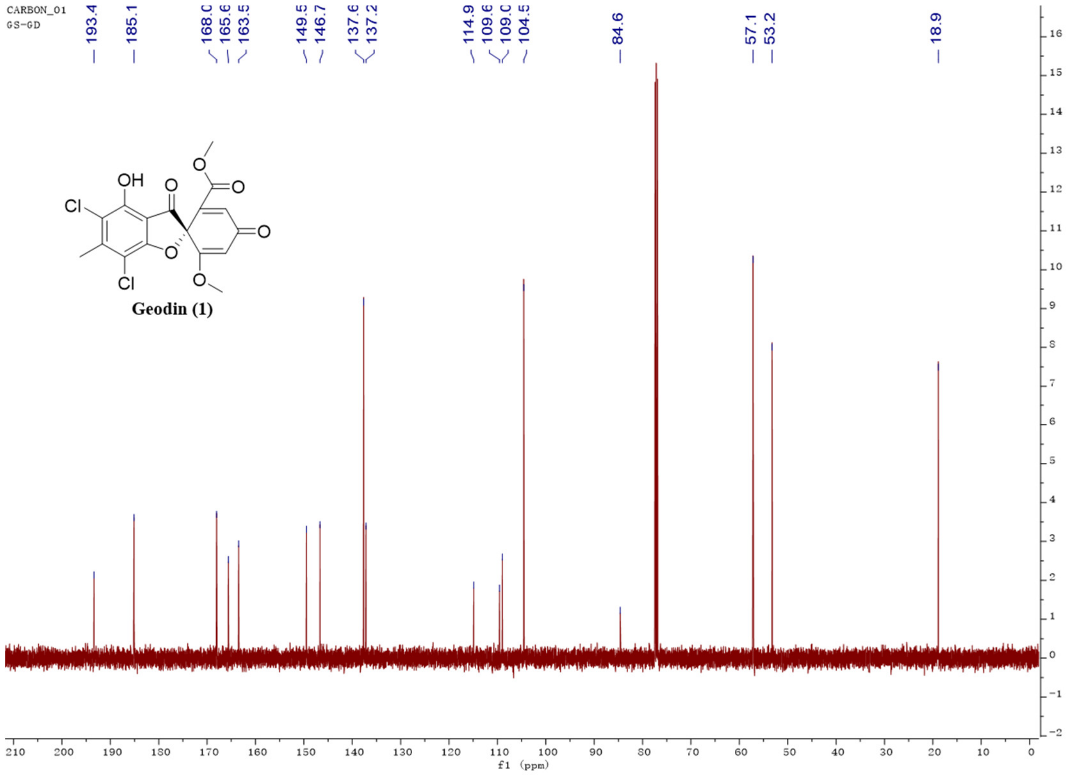

Figure S2. ^{13}C NMR (125 MHz, $CDCl_3$) spectrum of compound **1**

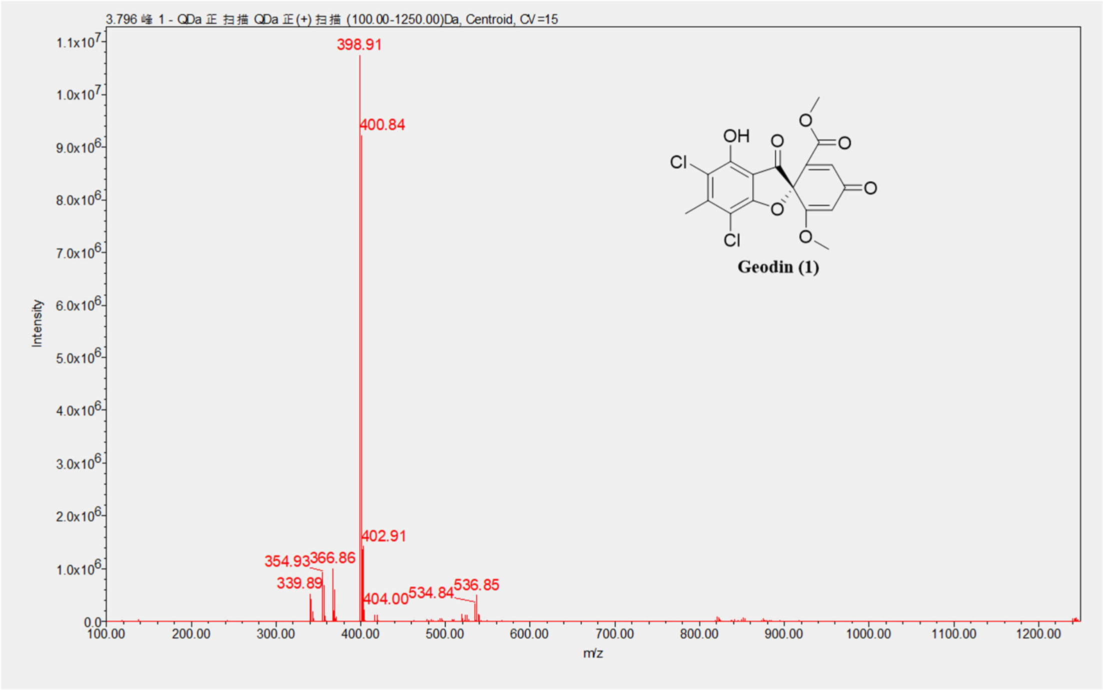

Figure S3. ESIMS spectrum of compound **1**.

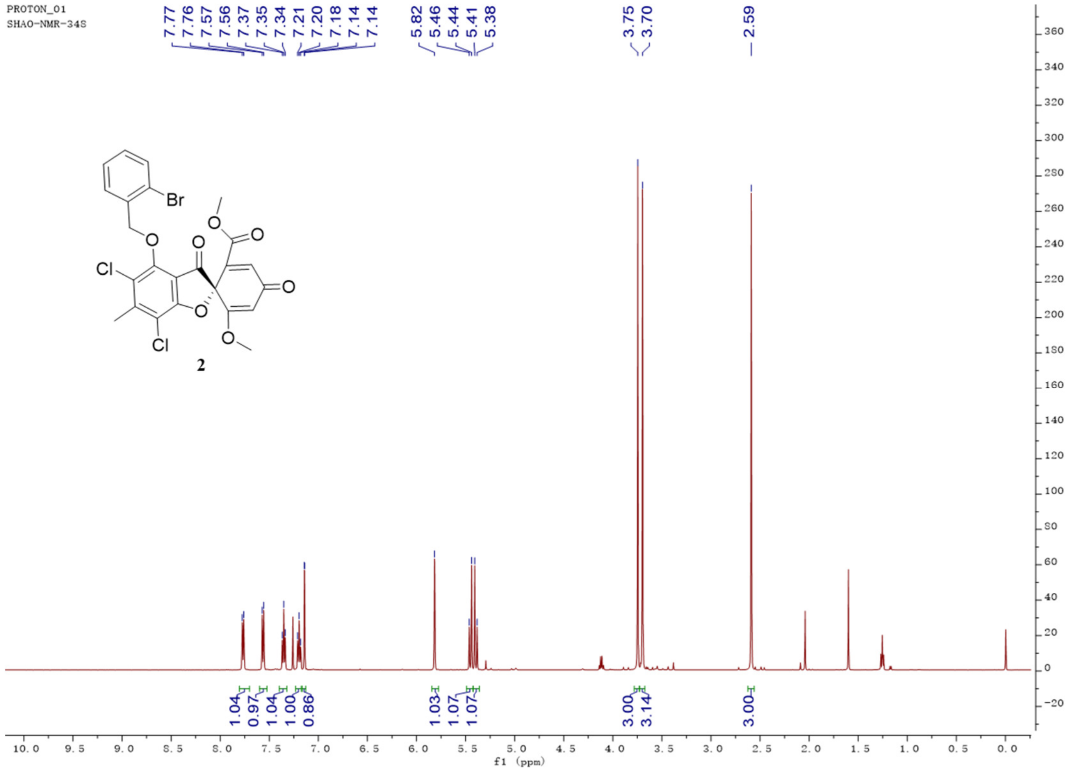

Figure S4. ^{1}H NMR (500 MHz, $CDCl_3$) spectrum of compound **2**

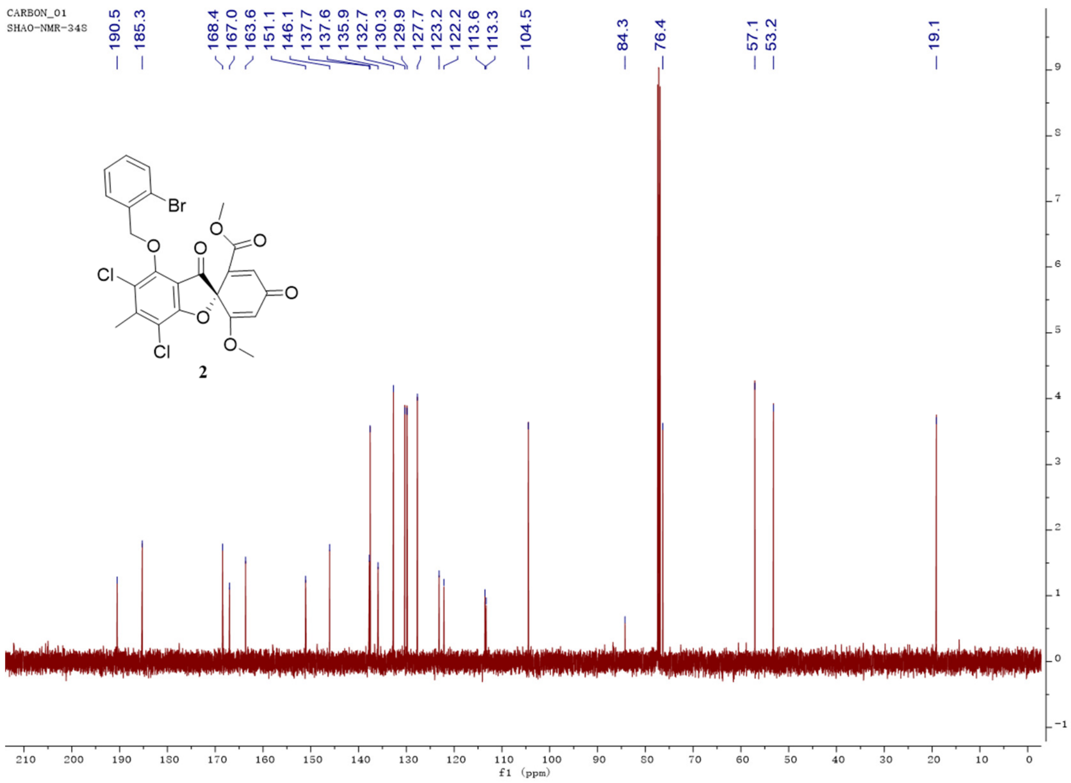

Figure S5. ^{13}C NMR (125 MHz, $CDCl_3$) spectrum of compound **2**

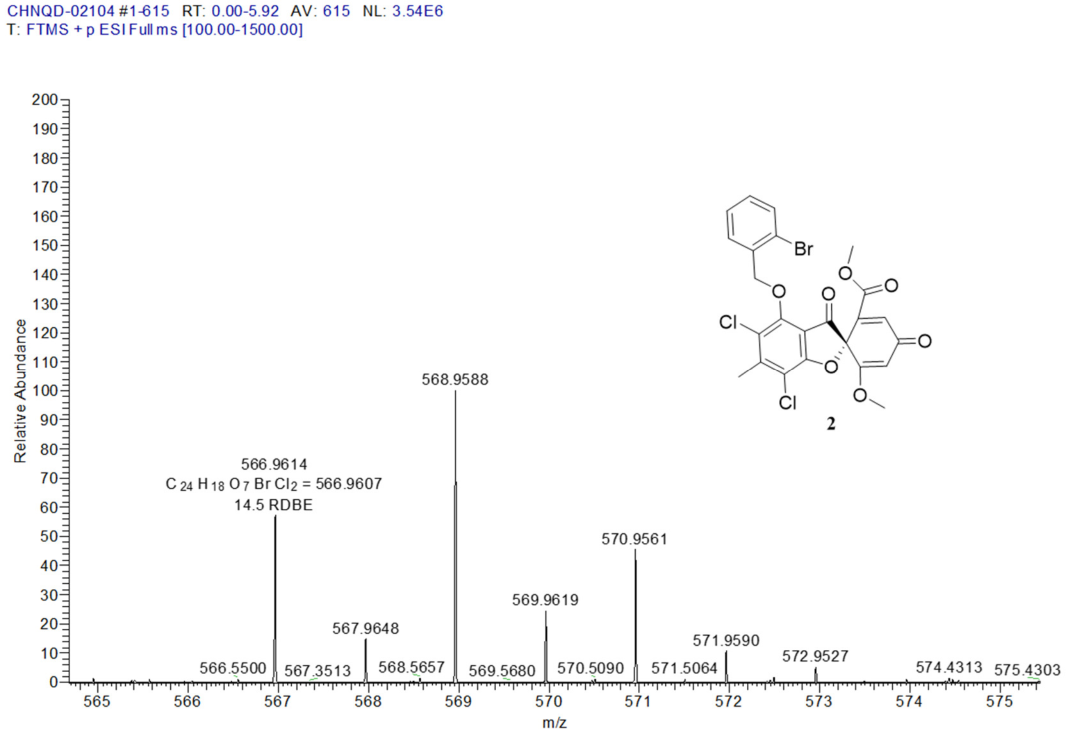

Figure S6. HRESIMS spectrum of compound **2**

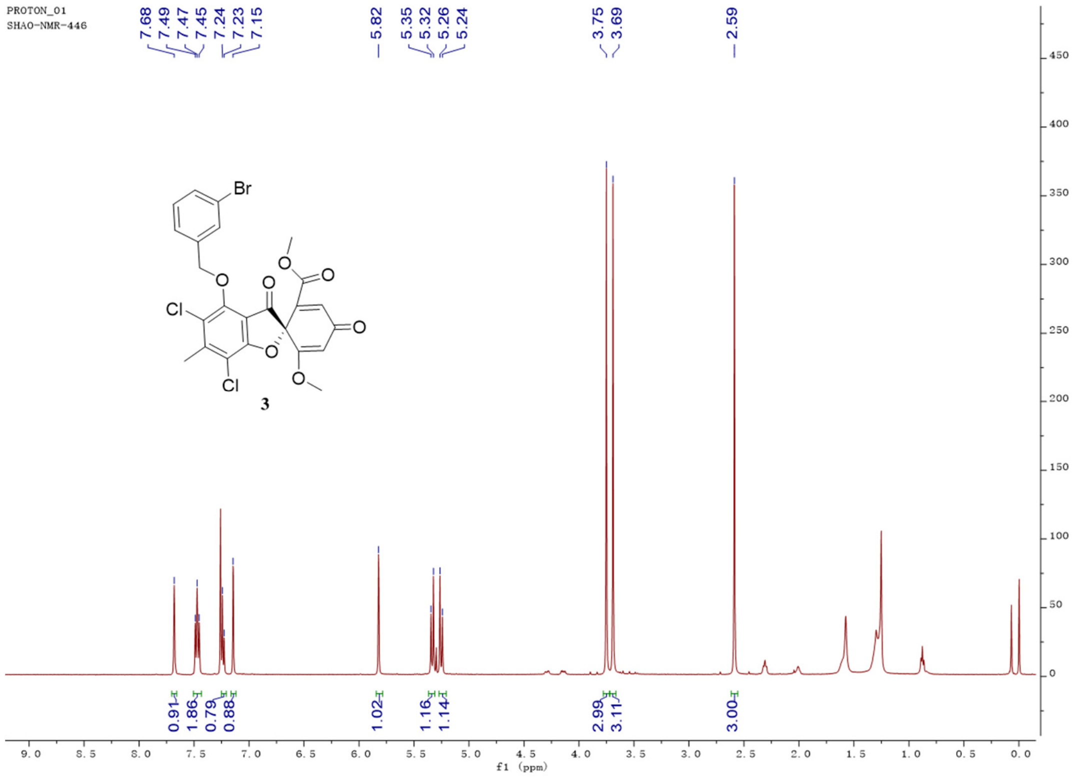

Figure S7. ^{1}H NMR (500 MHz, $CDCl_3$) spectrum of compound **3**

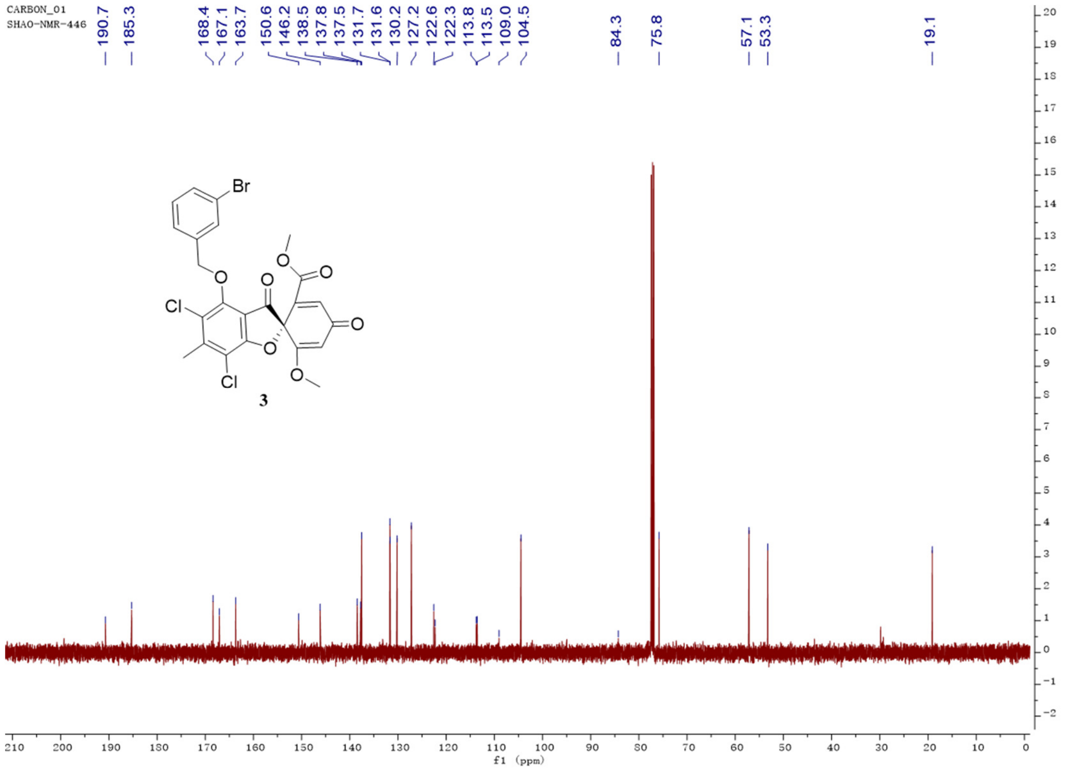

Figure S8. ^{13}C NMR (125 MHz, $CDCl_3$) spectrum of compound **3**

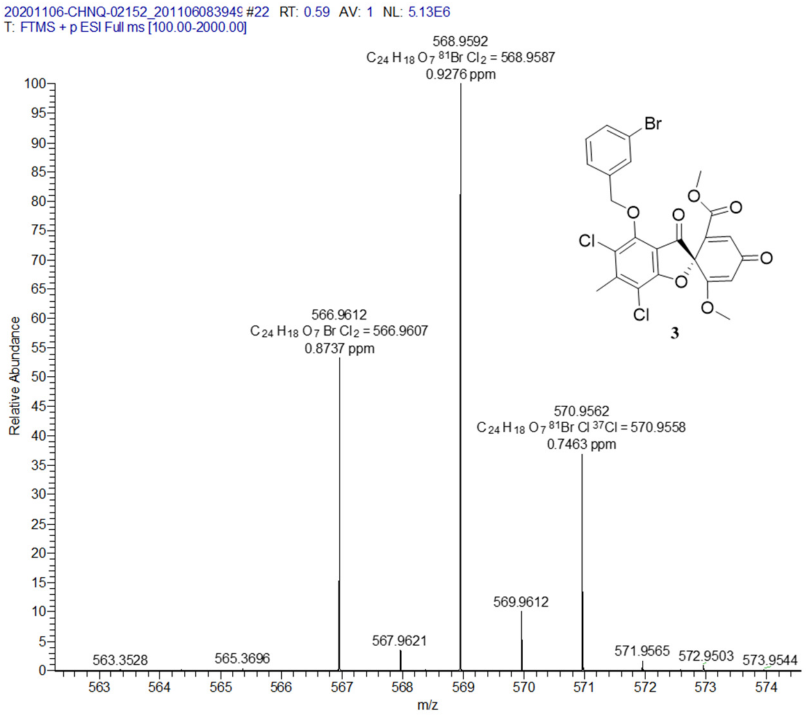

Figure S9. HRESIMS spectrum of compound **3**

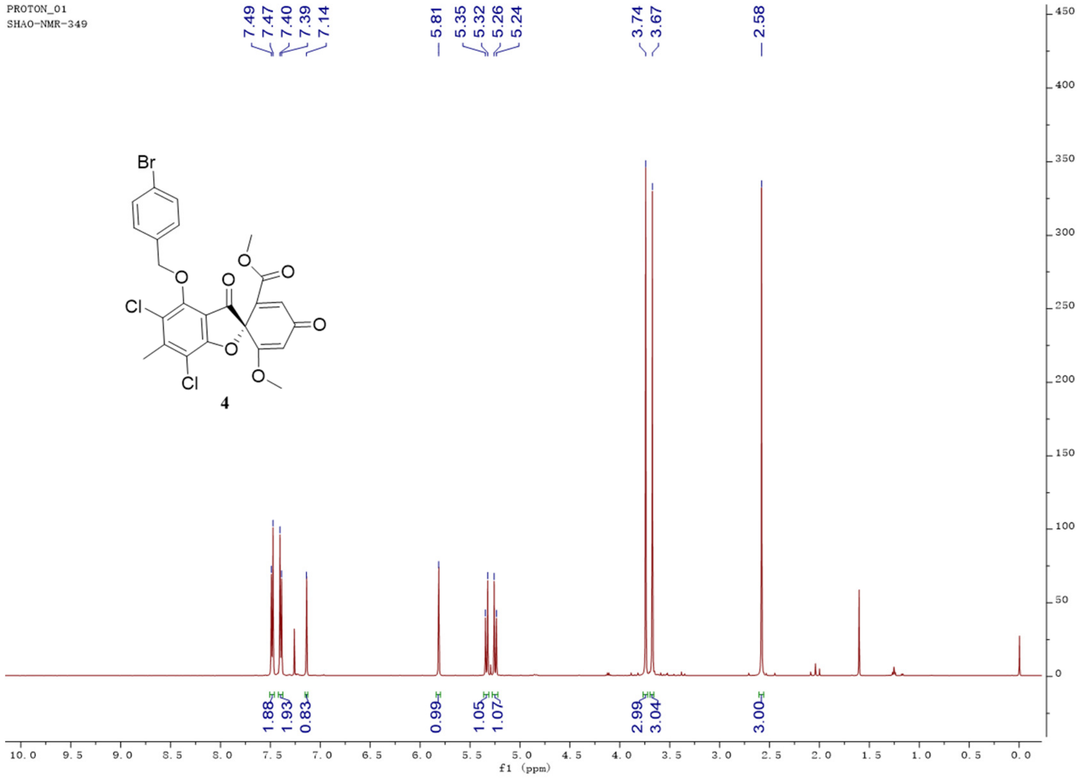

Figure S10. ^{1}H NMR (500 MHz, $CDCl_3$) spectrum of compound **4**

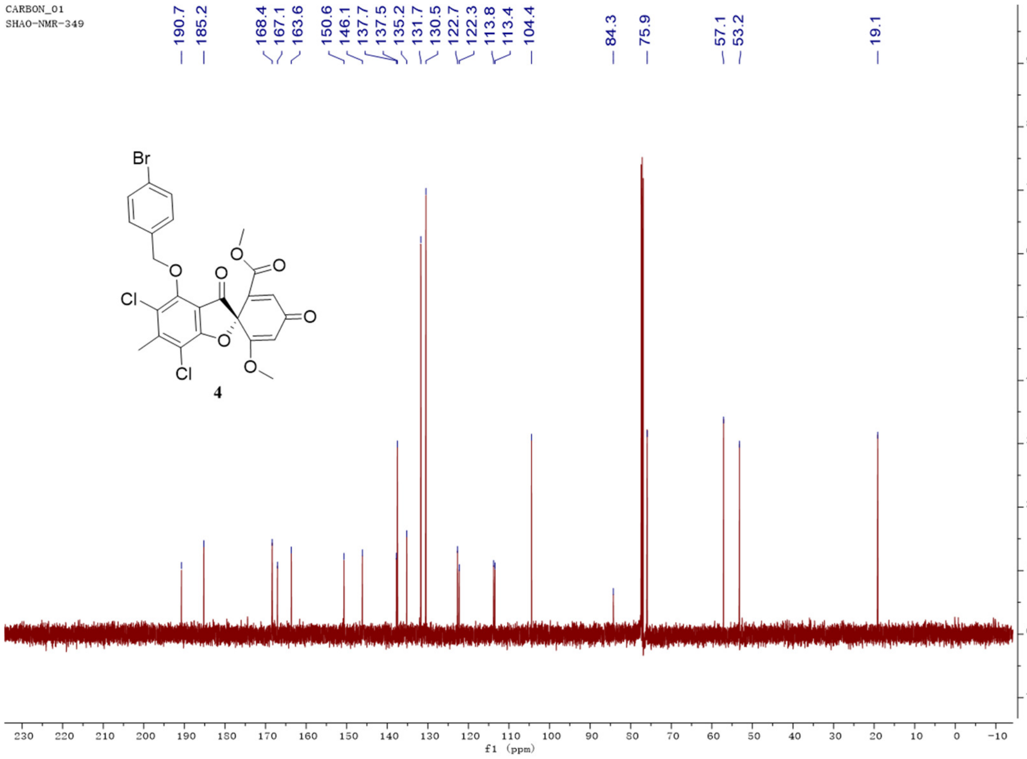

Figure S11. ^{13}C NMR (125 MHz, $CDCl_3$) spectrum of compound **4**

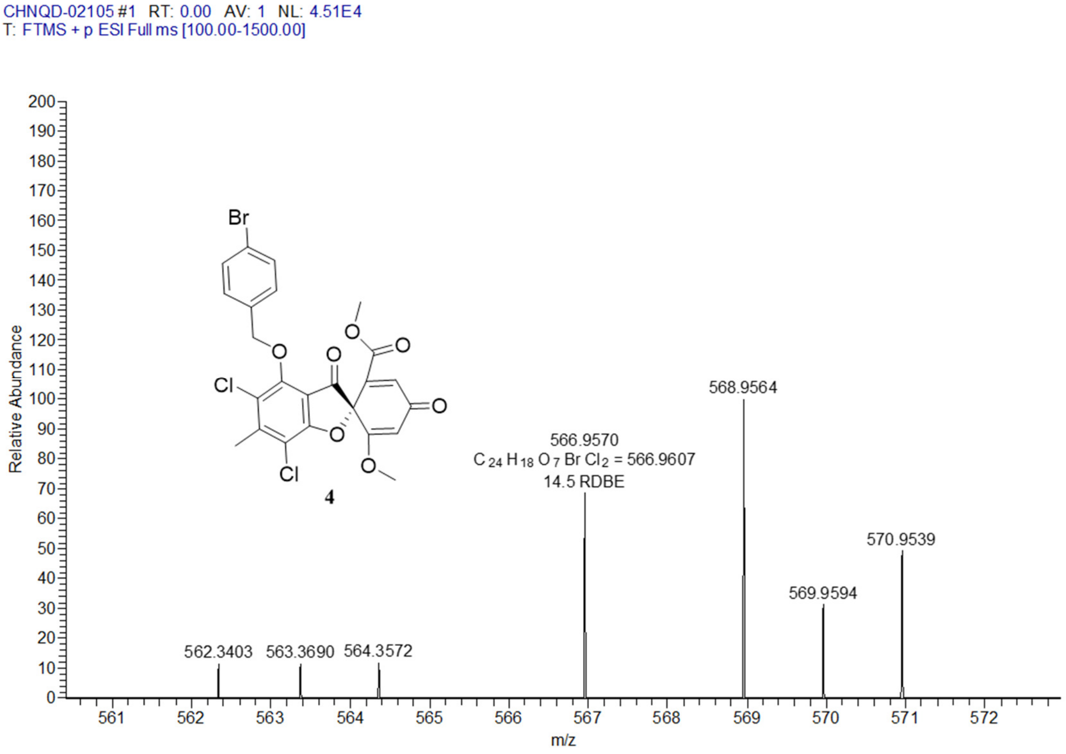

Figure S12. HRESIMS spectrum of compound **4**

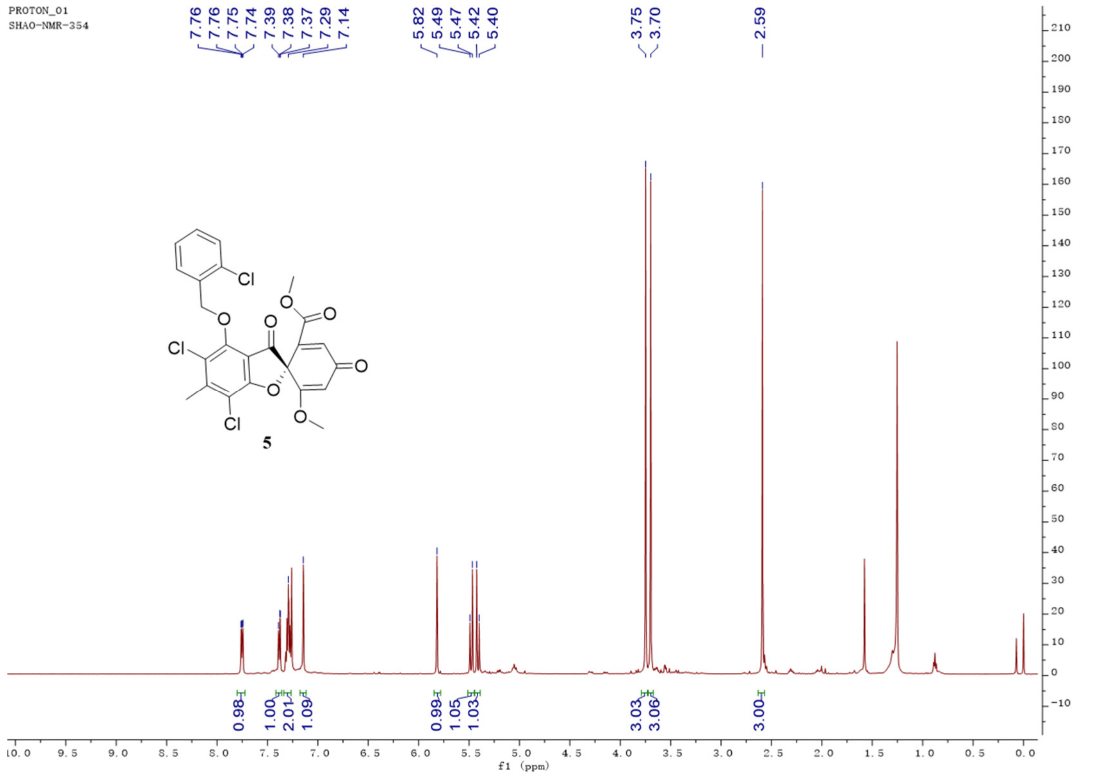

Figure S13. ^{1}H NMR (500 MHz, $CDCl_3$) spectrum of compound **5**

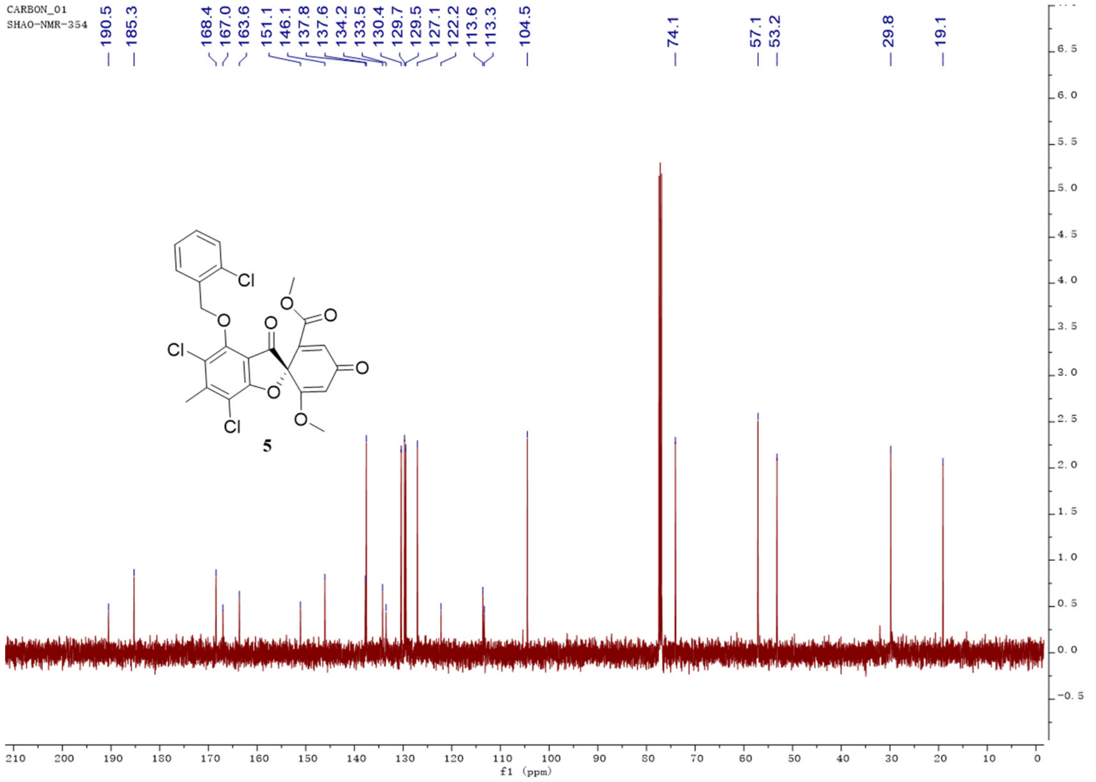

Figure S14. ^{13}C NMR (125 MHz, $CDCl_3$) spectrum of compound **5**

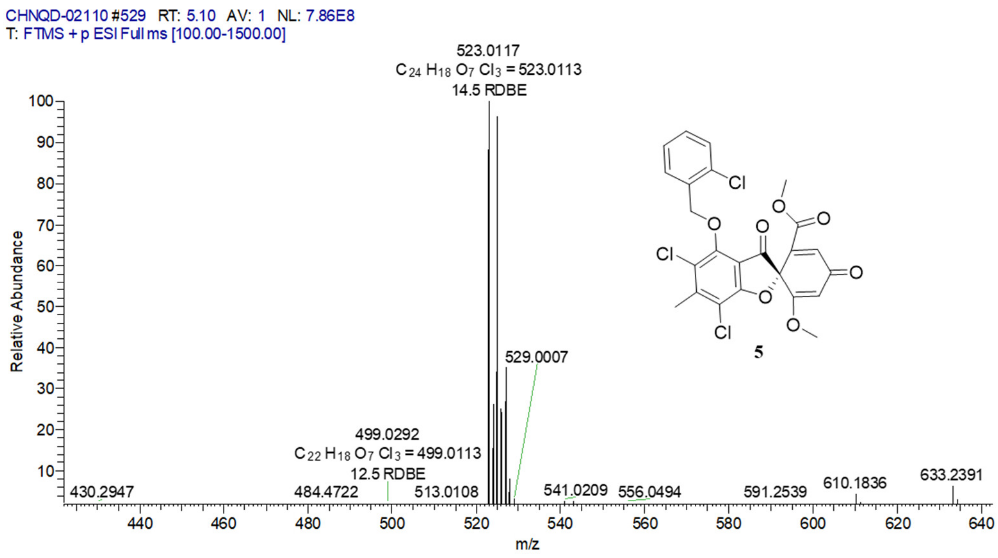

Figure S15. HRESIMS spectrum of compound **5**

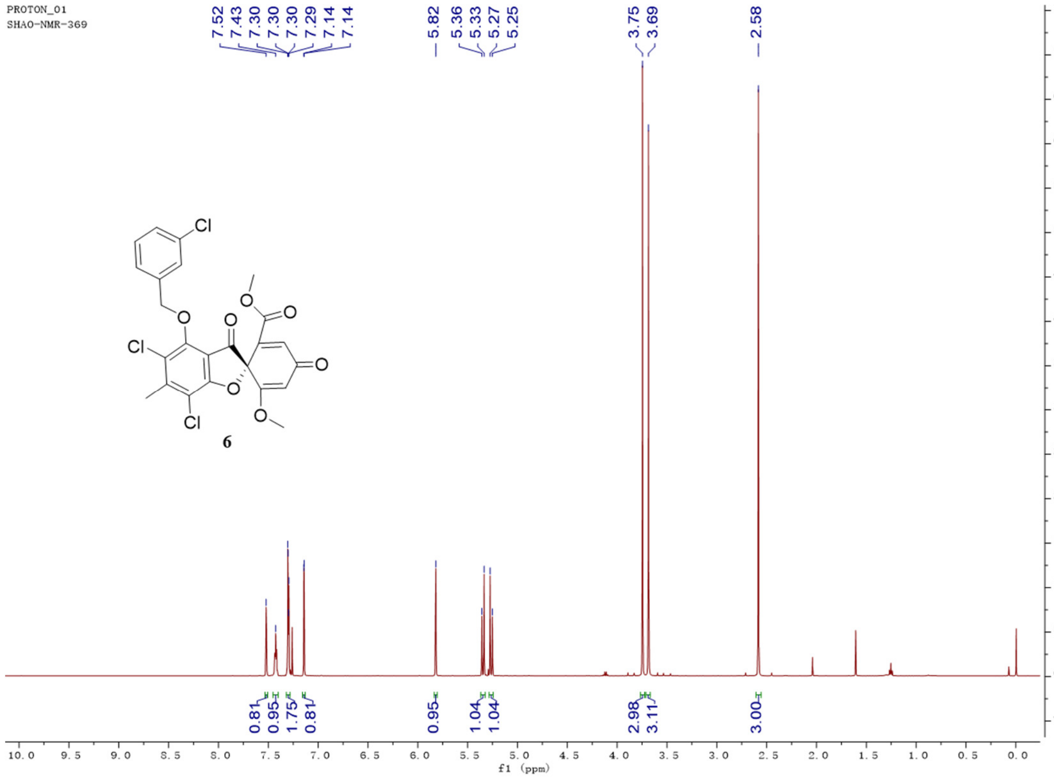

Figure S16. ¹H NMR (500 MHz, $CDCl_3$) spectrum of compound **6**

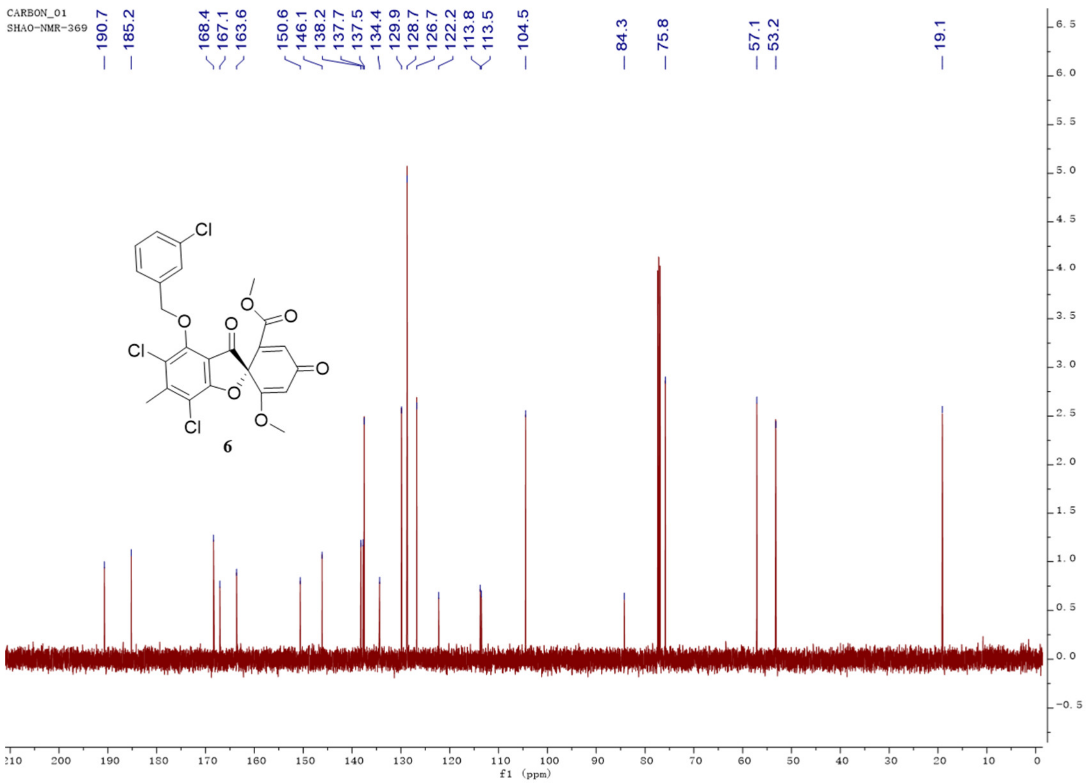

Figure S17. ^{13}C NMR (125 MHz, $CDCl_3$) spectrum of compound **6**

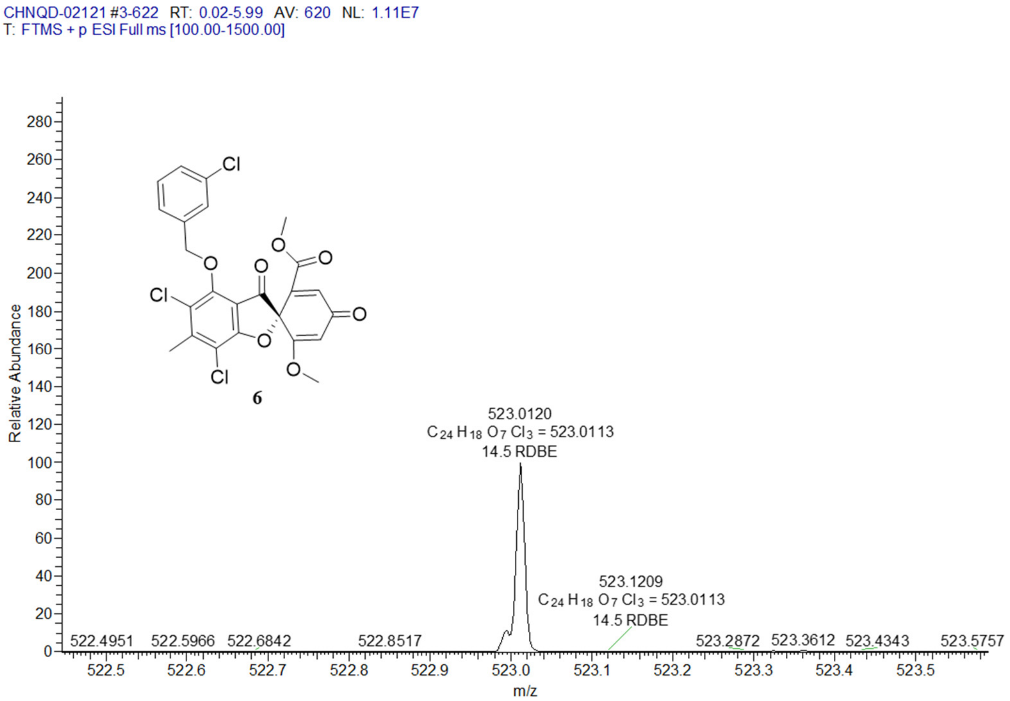

Figure S18. HRESIMS spectrum of compound **6**

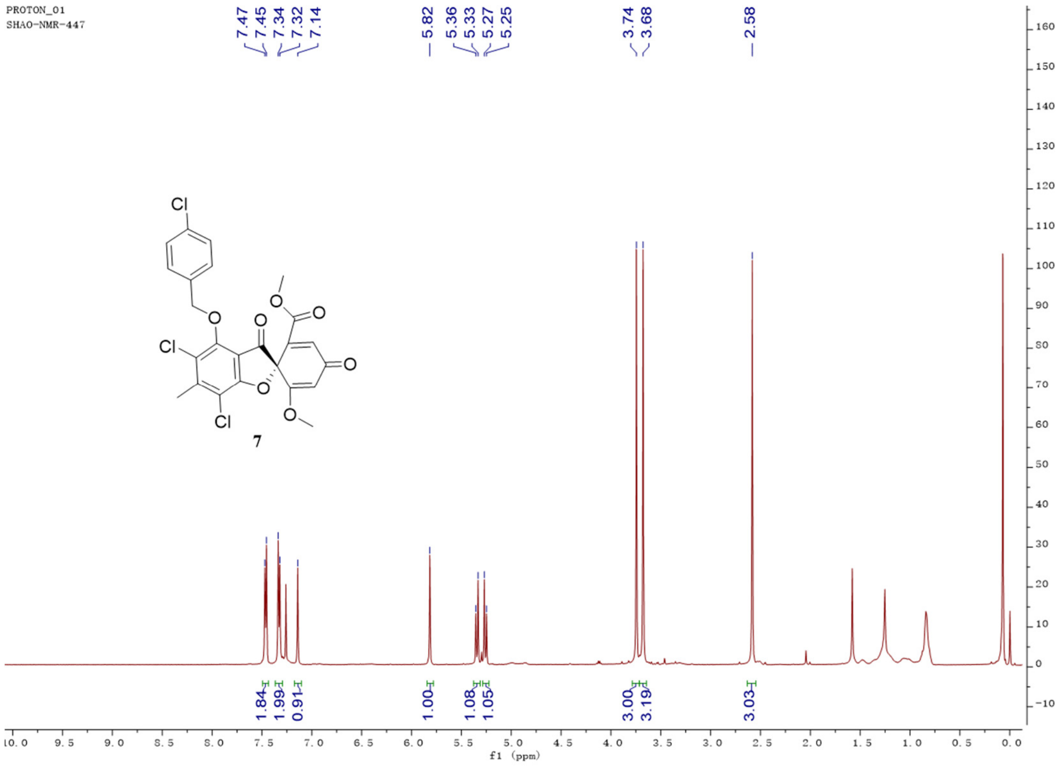

Figure S19. ^{1}H NMR (500 MHz, $CDCl_3$) spectrum of compound **7**

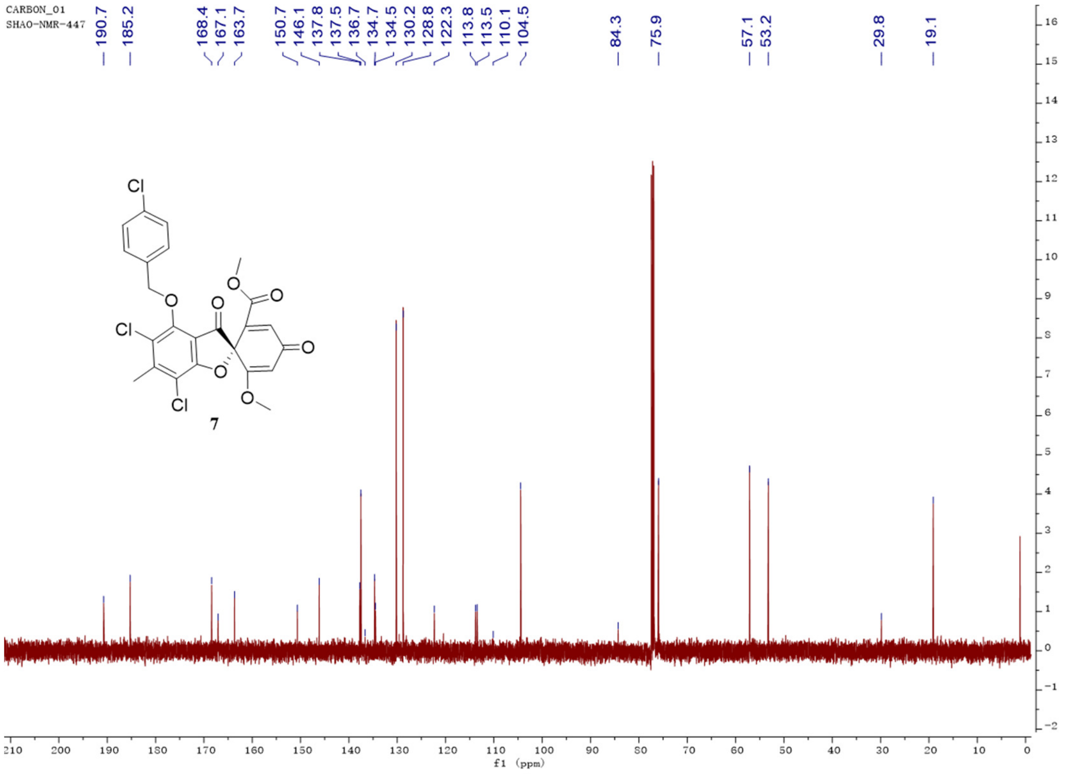

Figure S20. ^{13}C NMR (125 MHz, $CDCl_3$) spectrum of compound **7**

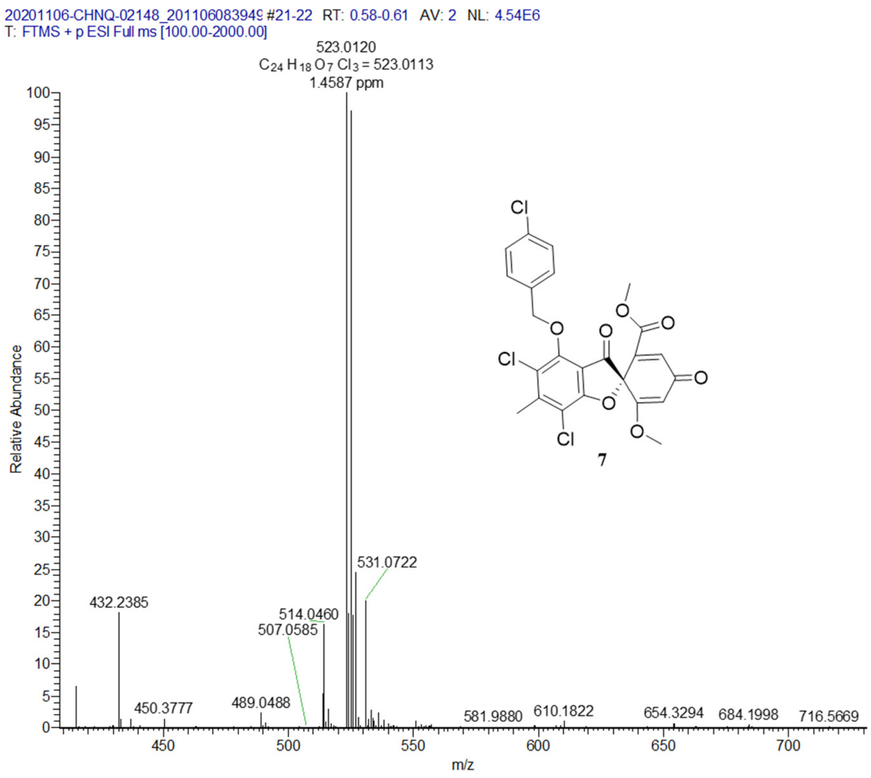

Figure S21. HRESIMS spectrum of compound **7**

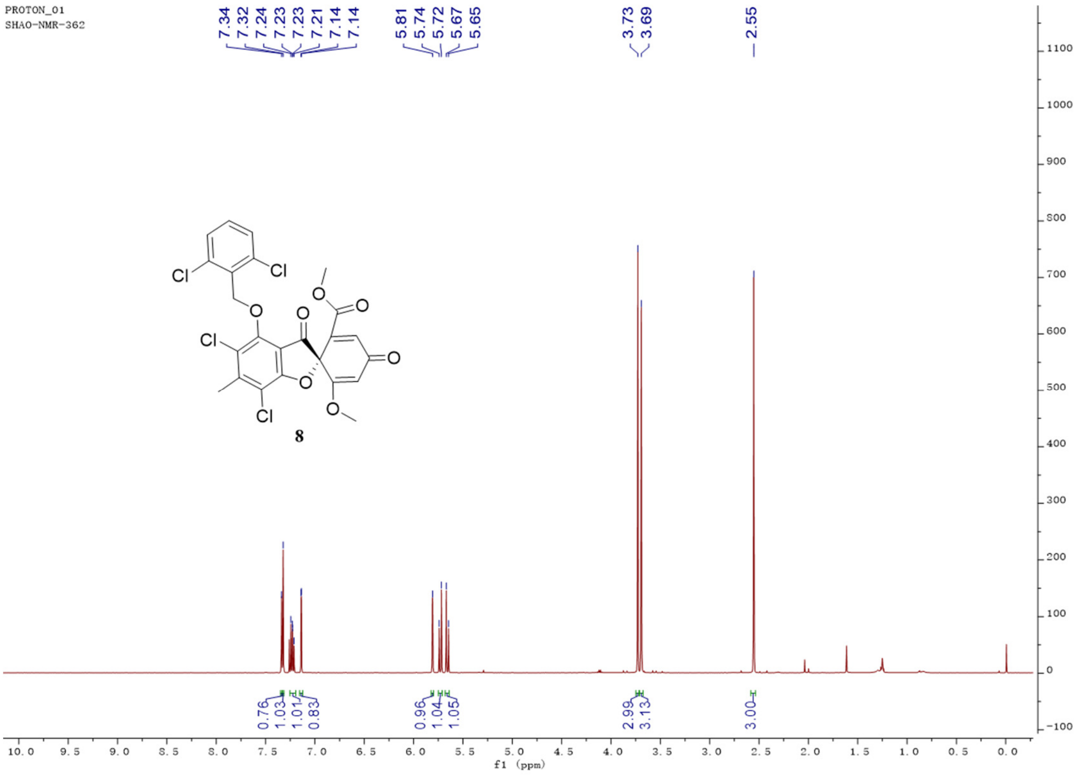

Figure S22. ^{1}H NMR (500 MHz, $CDCl_3$) spectrum of compound **8**

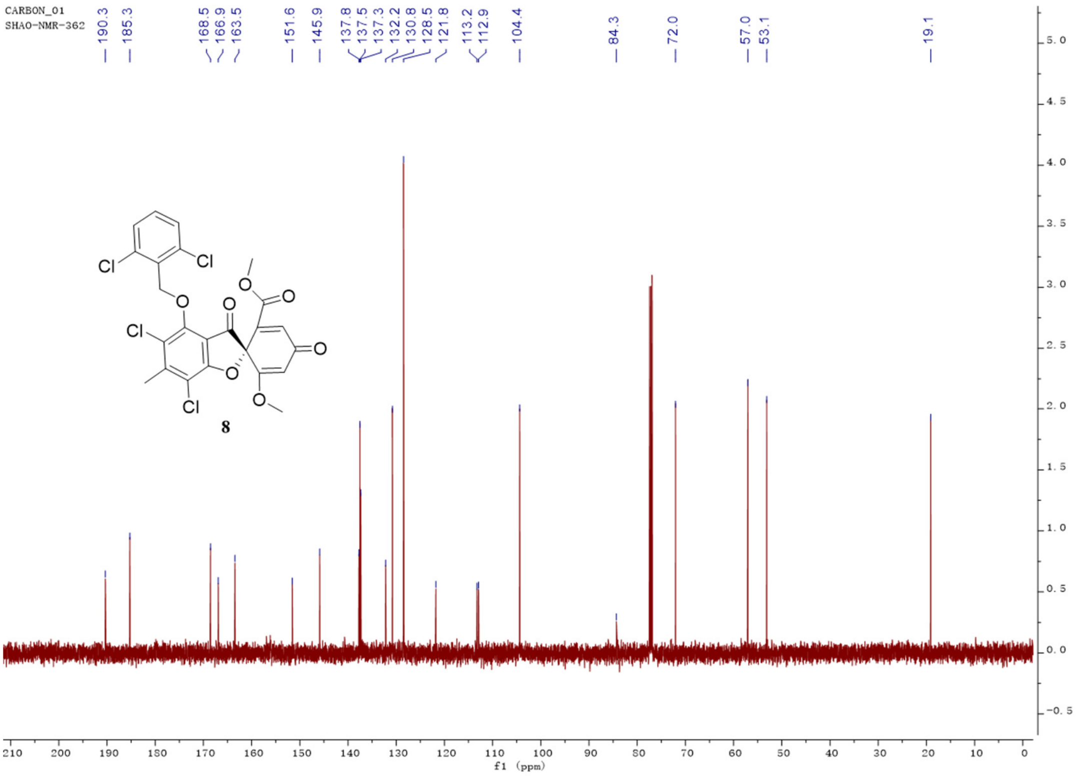

Figure S23. ^{13}C NMR (125 MHz, $CDCl_3$) spectrum of compound **8**

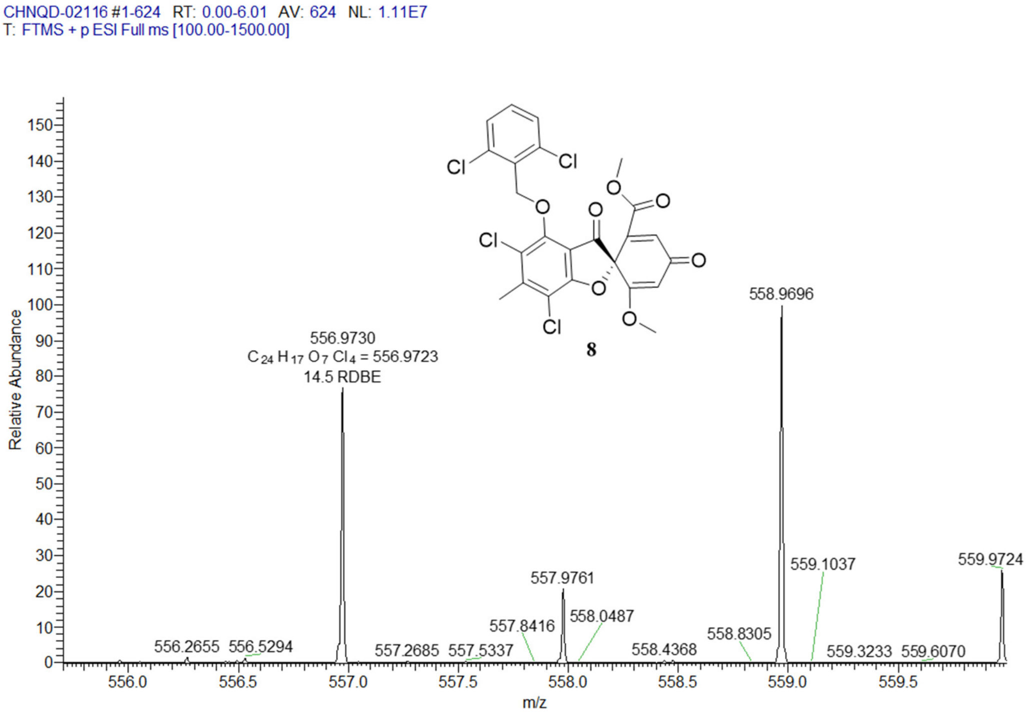

Figure S24. HRESIMS spectrum of compound **8**

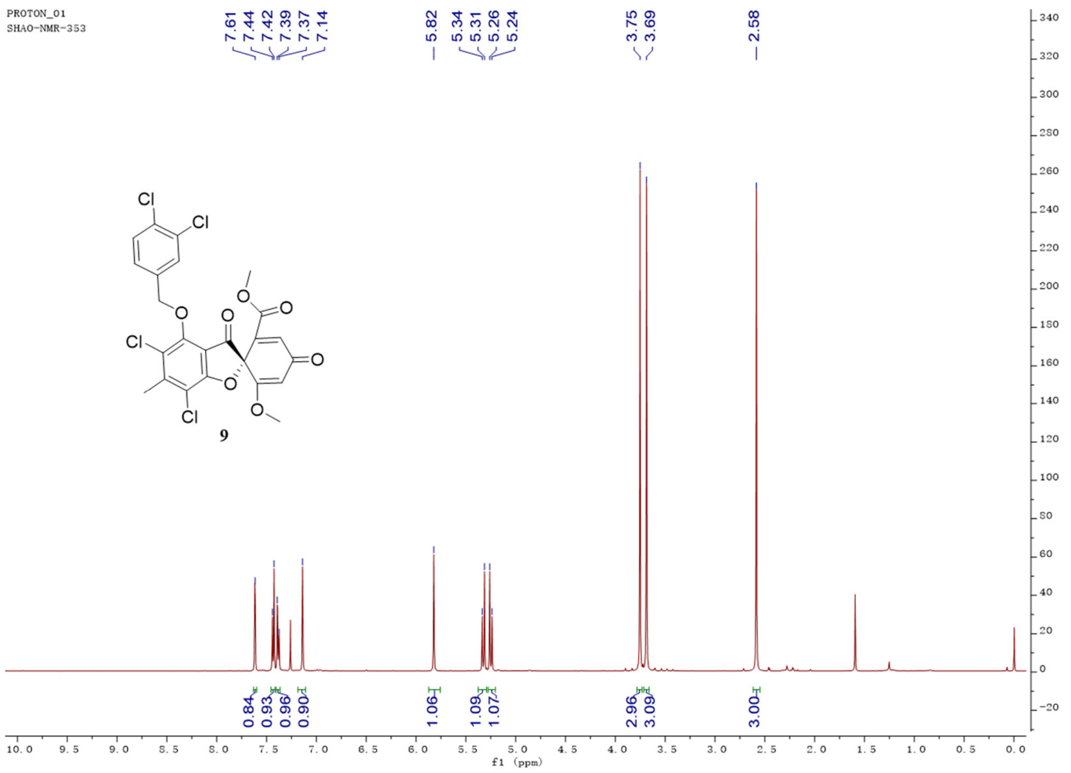

Figure S25. ^{1}H NMR (500 MHz, $CDCl_3$) spectrum of compound **9**

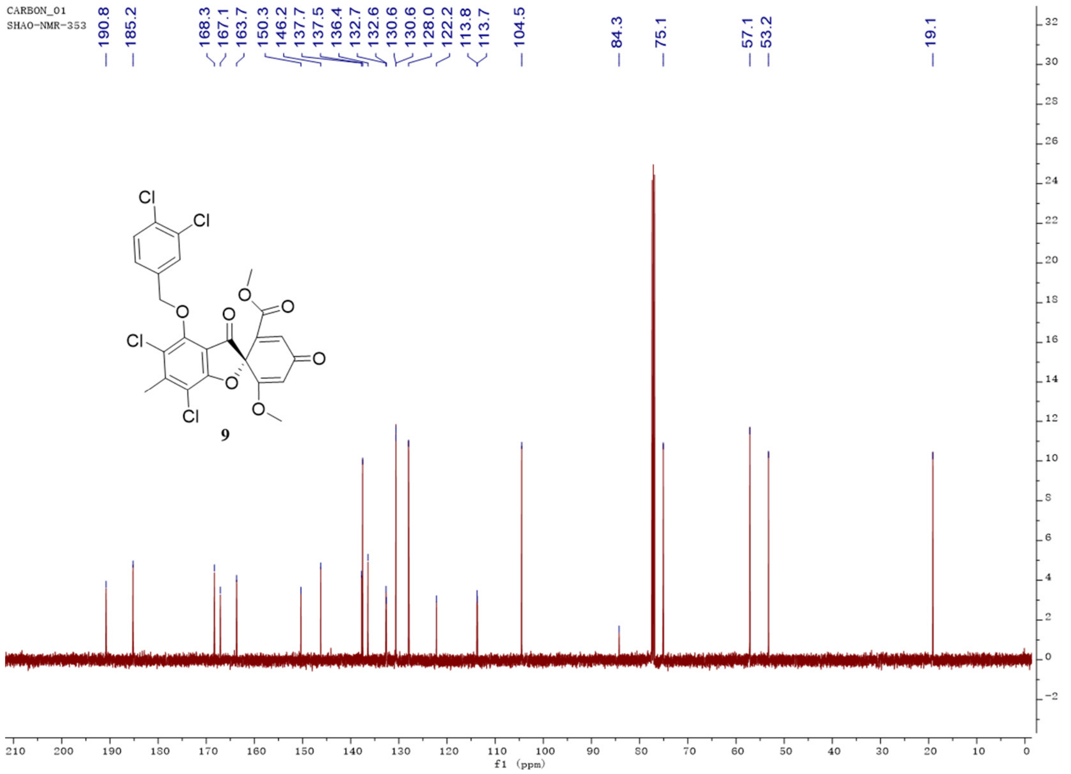

Figure S26. ^{13}C NMR (125 MHz, $CDCl_3$) spectrum of compound **9**

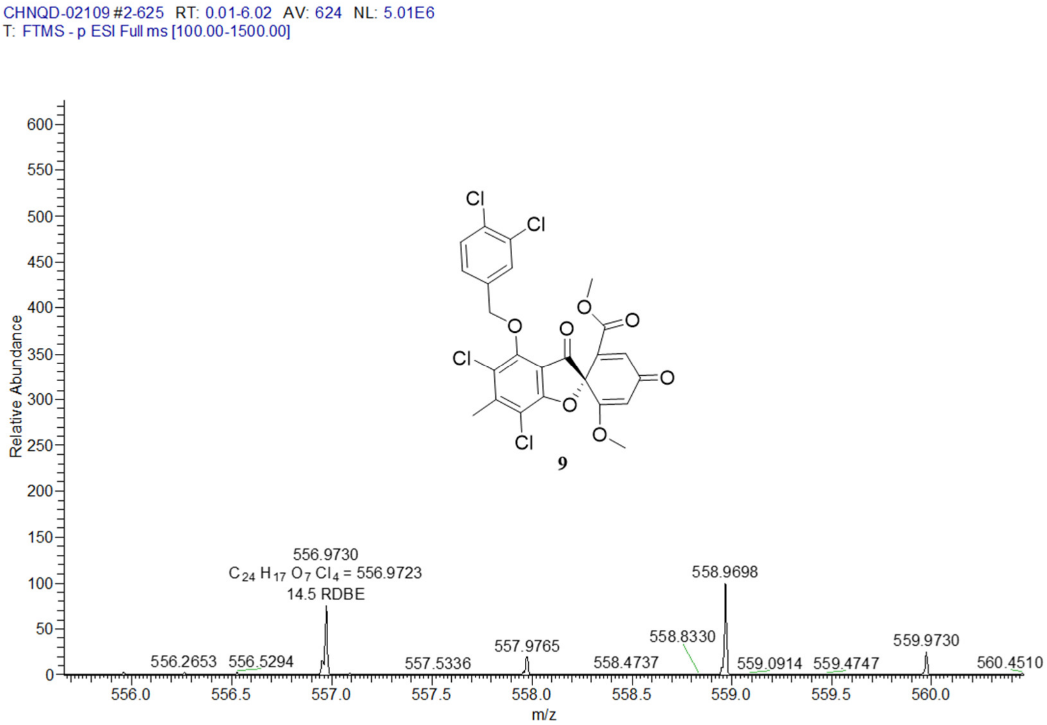

Figure S27. HRESIMS spectrum of compound **9**

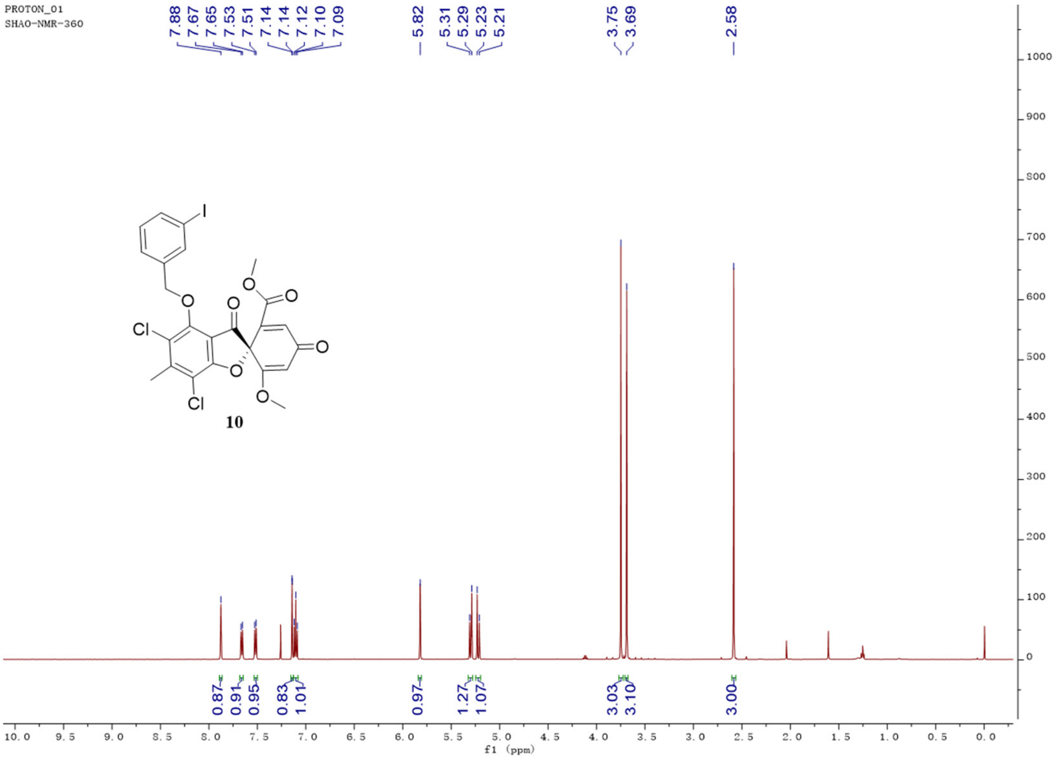

Figure S28. ^{1}H NMR (500 MHz, $CDCl_3$) spectrum of compound **10**

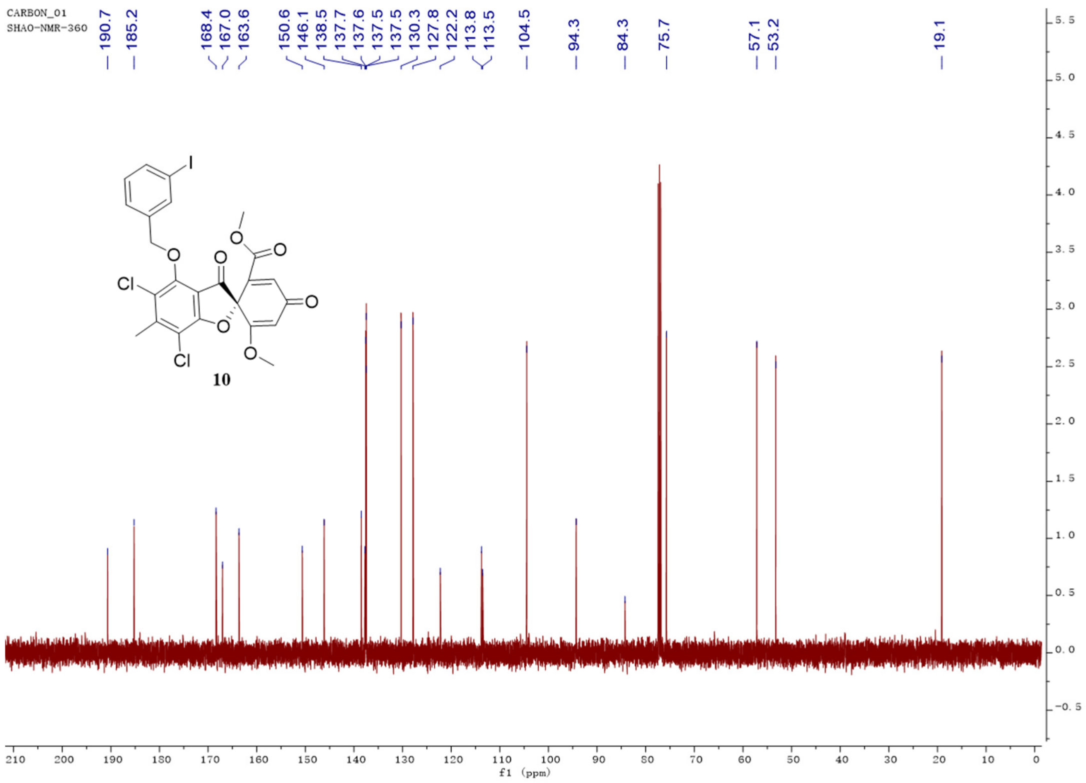

Figure S29. ^{13}C NMR (125 MHz, $CDCl_3$) spectrum of compound **10**

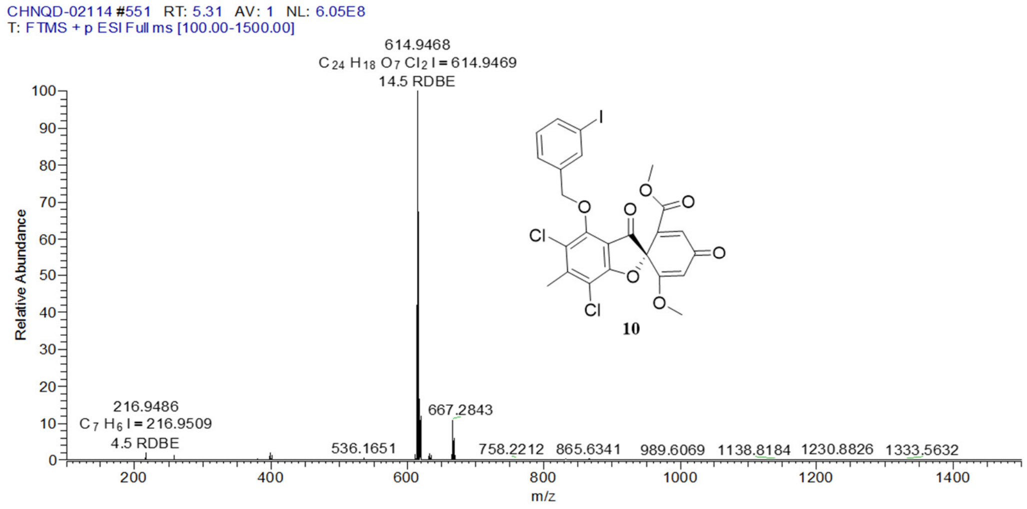

Figure S30. HRESIMS spectrum of compound **10**

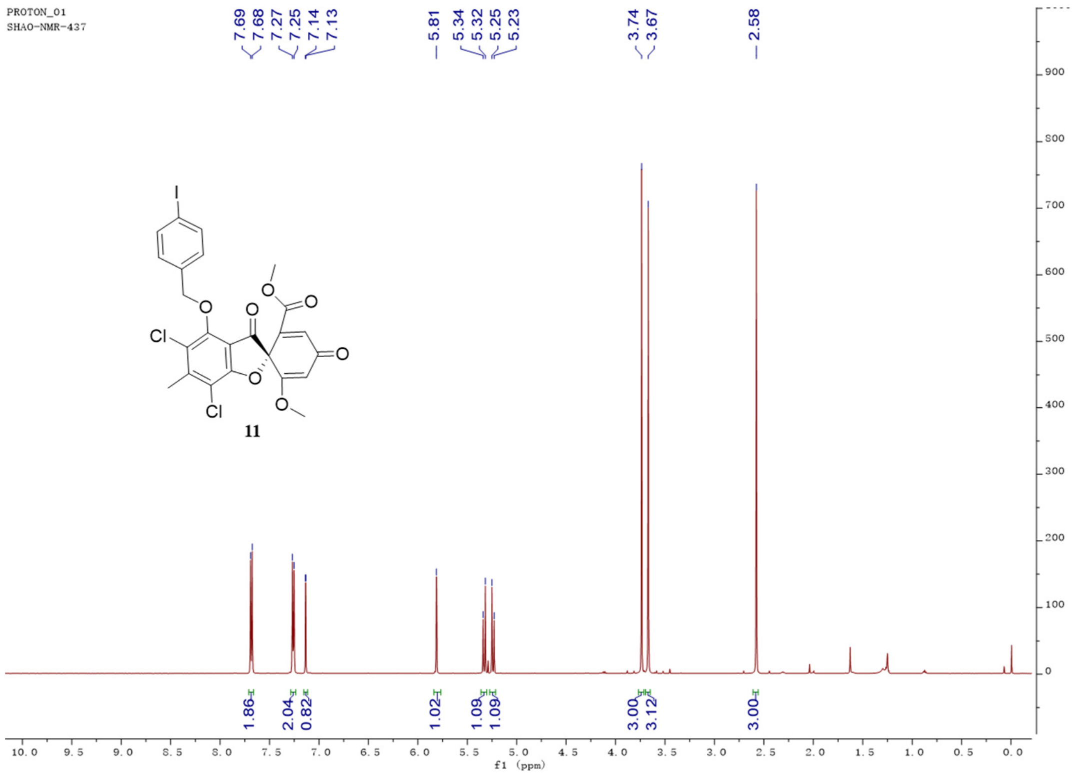

Figure S31. ^{1}H NMR (500 MHz, $CDCl_3$) spectrum of compound **11**

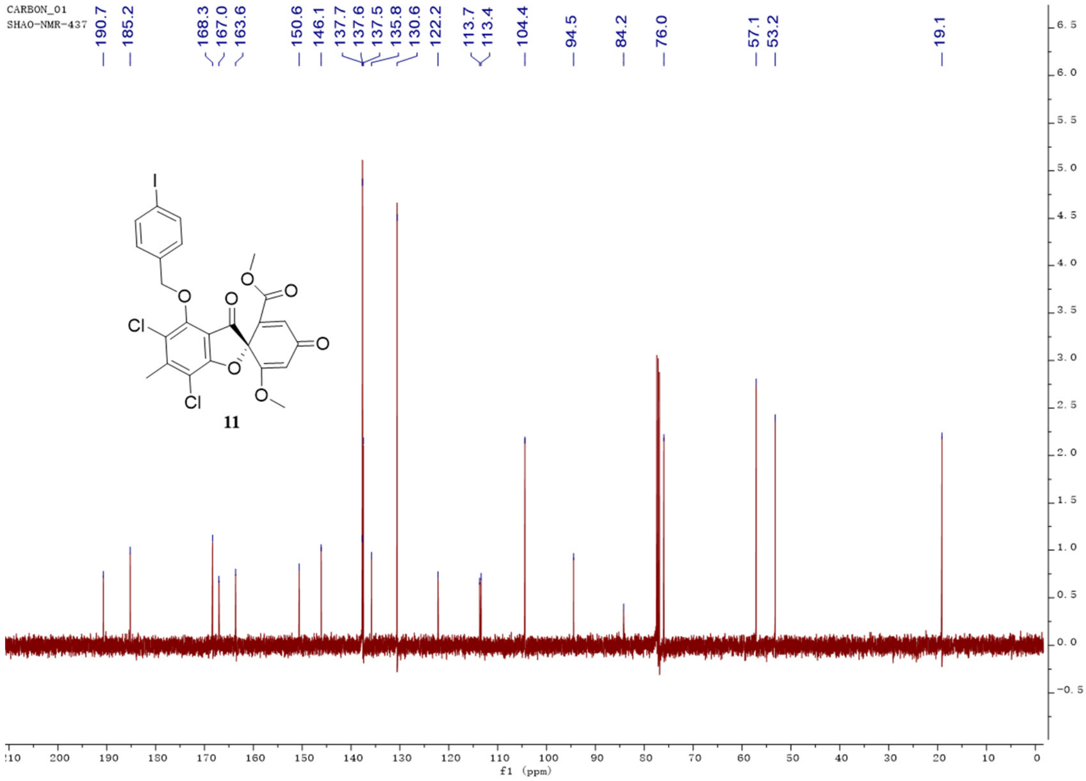

Figure S32. ^{13}C NMR (125 MHz, $CDCl_3$) spectrum of compound **11**

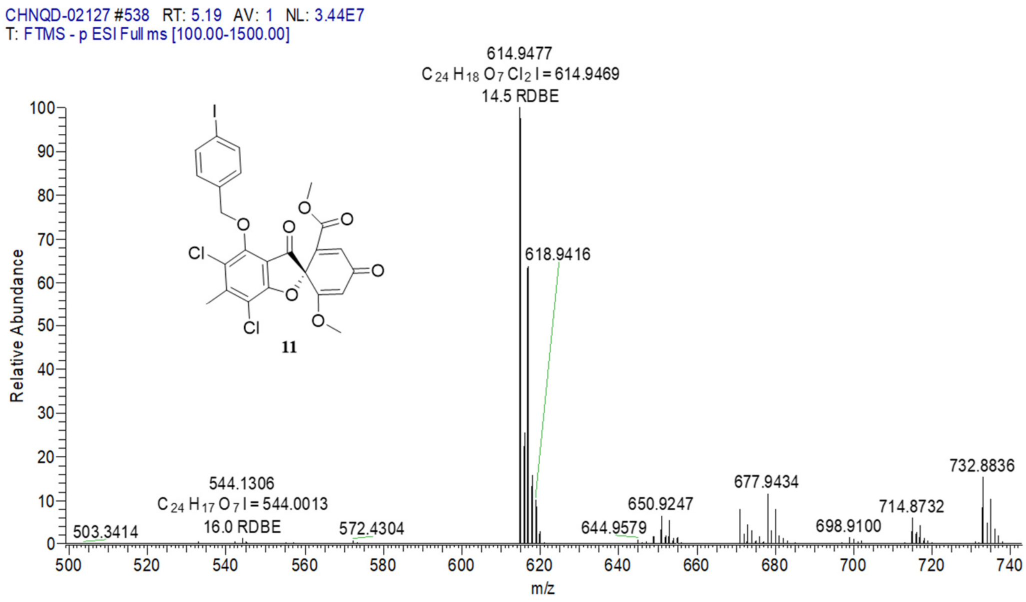

Figure S33. HRESIMS spectrum of compound **11**

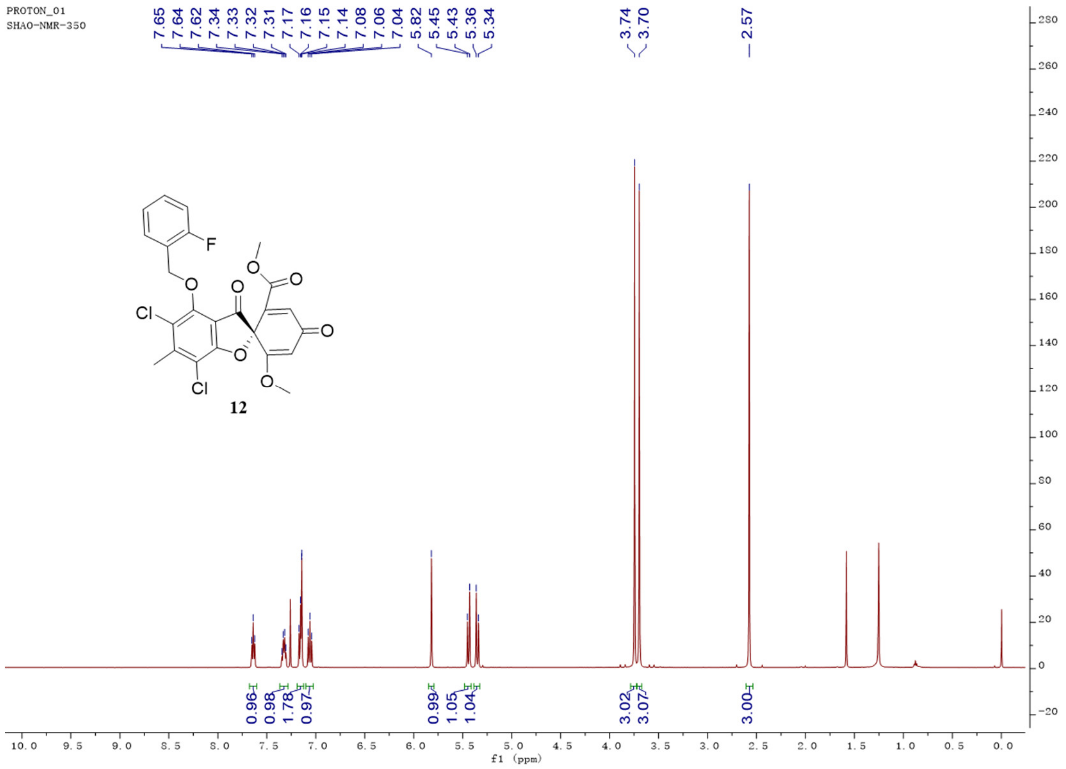

Figure S34. ^{1}H NMR (500 MHz, $CDCl_3$) spectrum of compound **12**

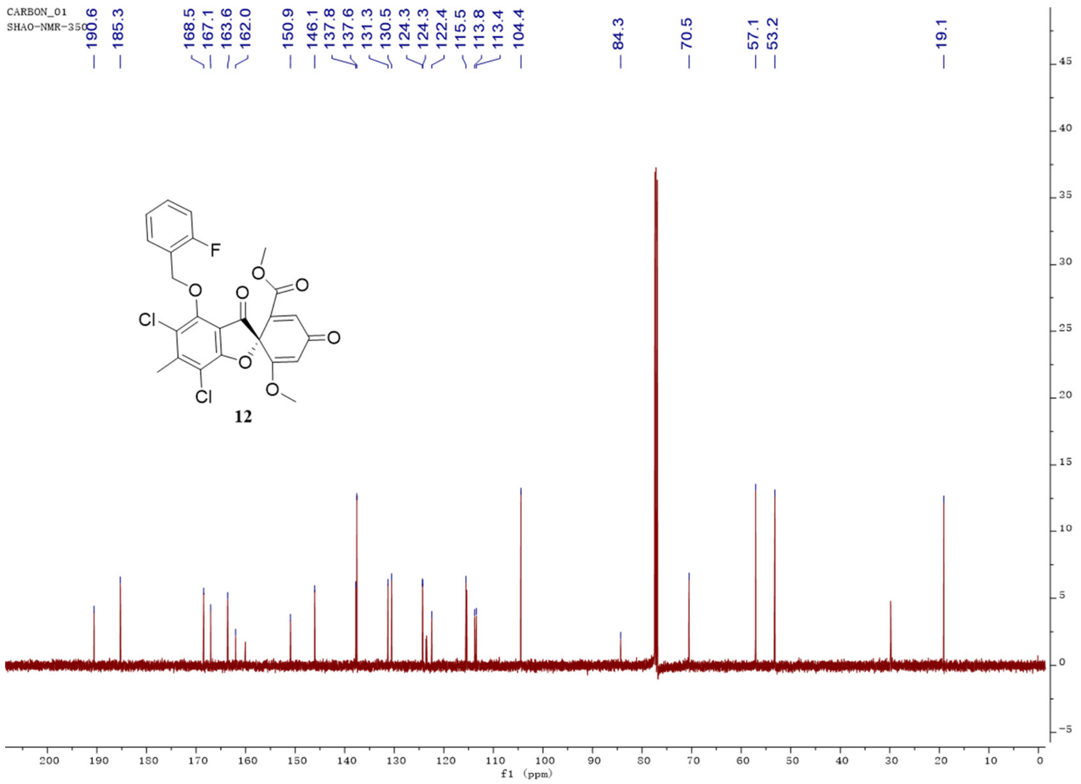

Figure S35. ^{13}C NMR (125 MHz, $CDCl_3$) spectrum of compound **12**

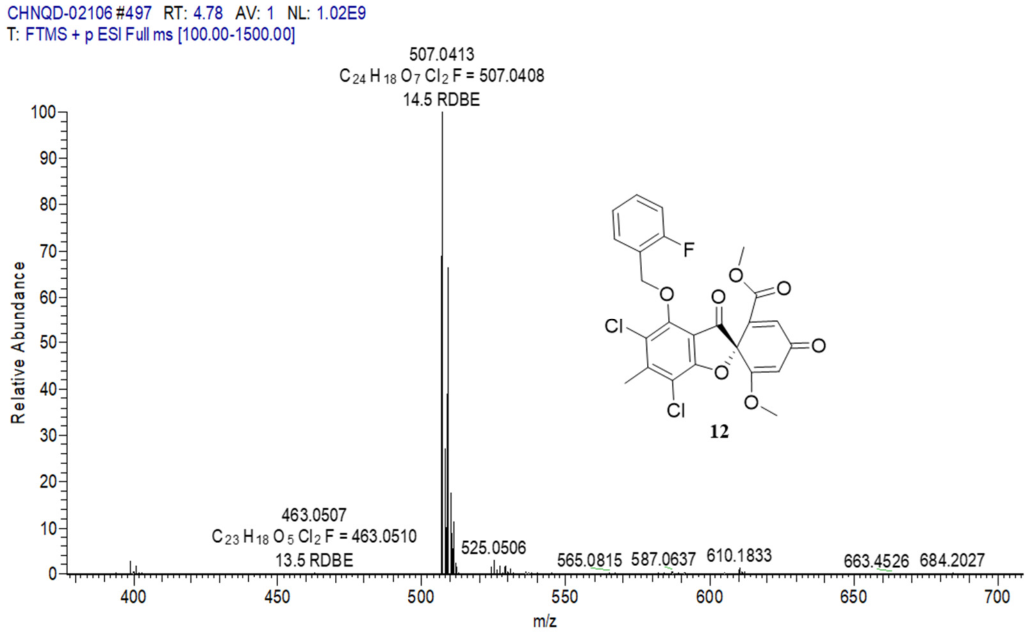

Figure S36. HRESIMS spectrum of compound **12**

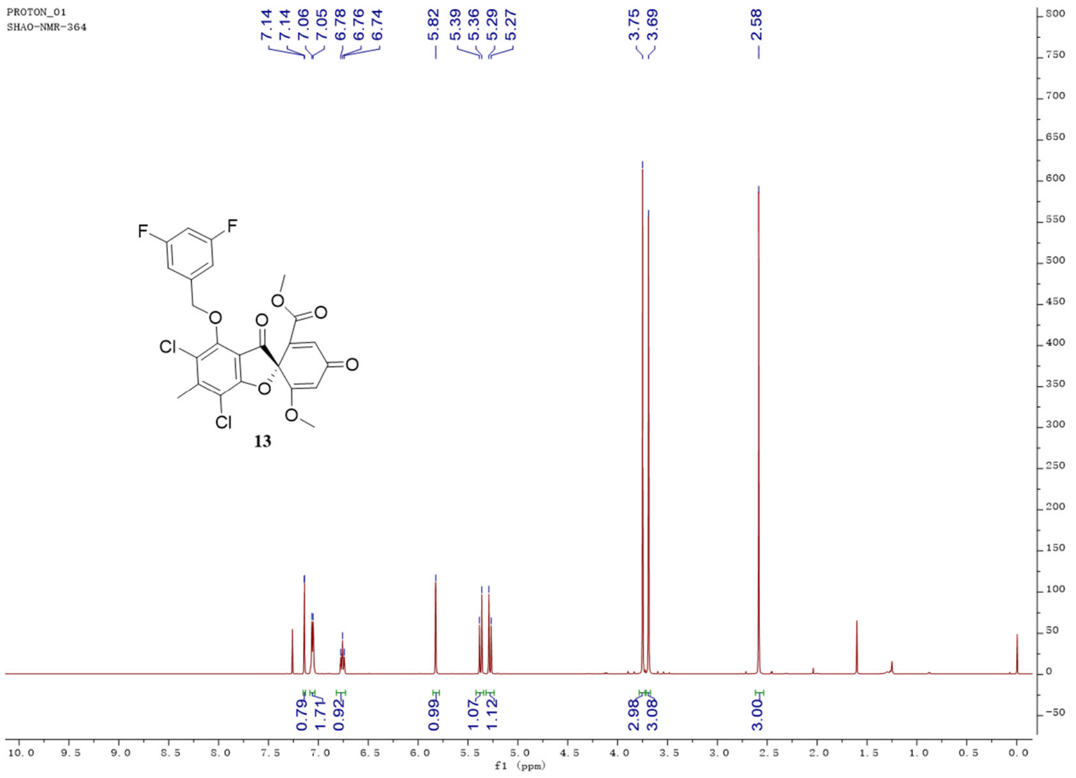

Figure S37. ^{1}H NMR (500 MHz, $CDCl_3$) spectrum of compound **13**

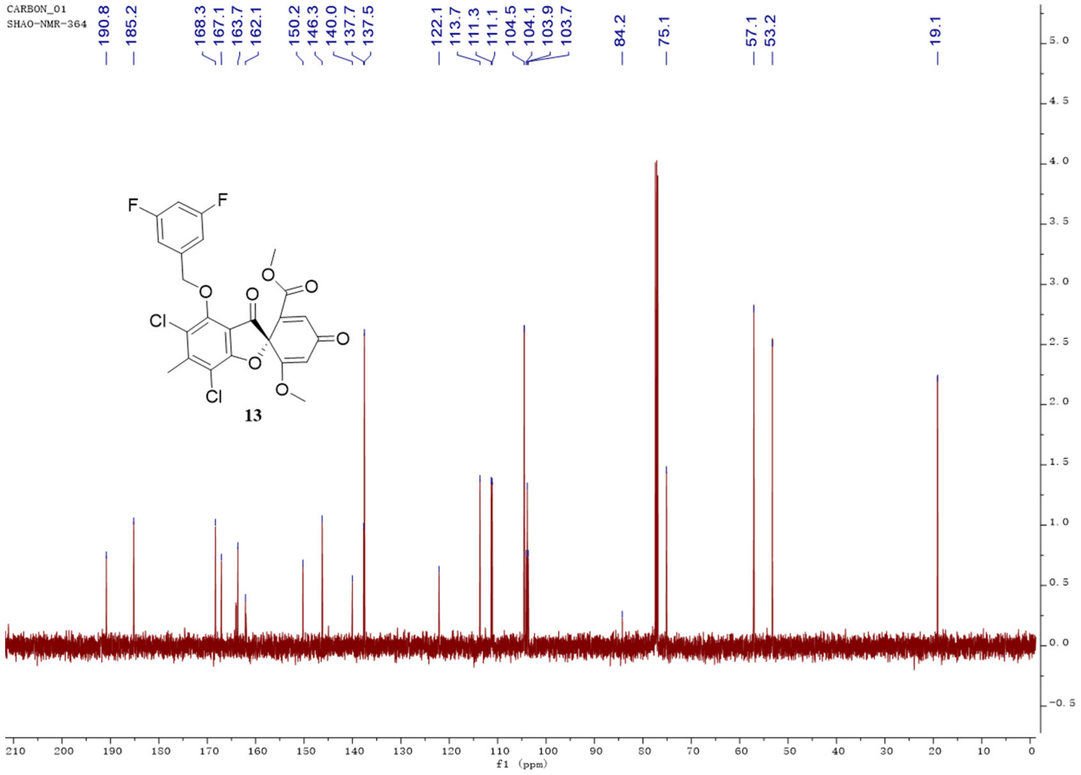

Figure S38. ^{13}C NMR (125 MHz, $CDCl_3$) spectrum of compound **13**

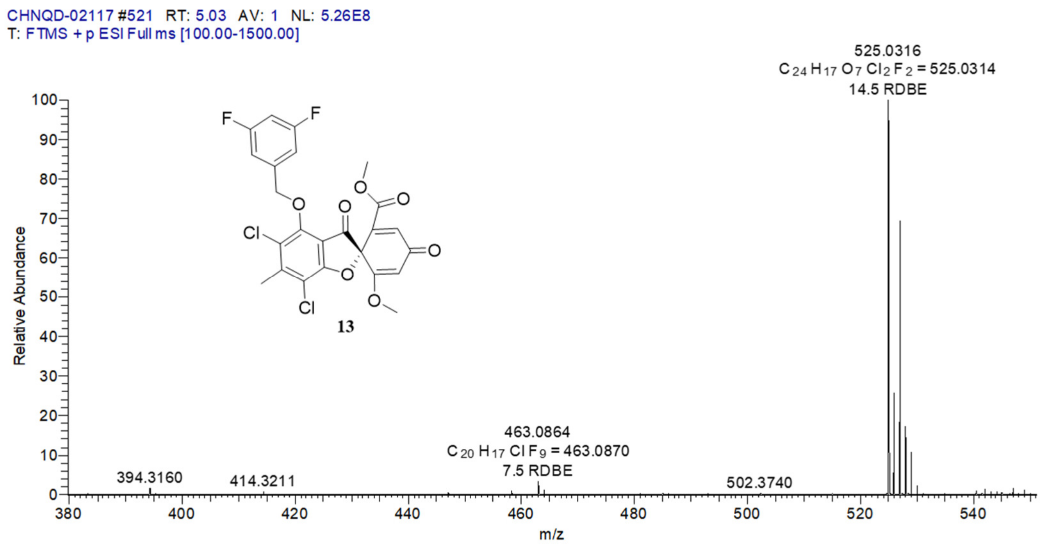

Figure S39. HRESIMS spectrum of compound **13**

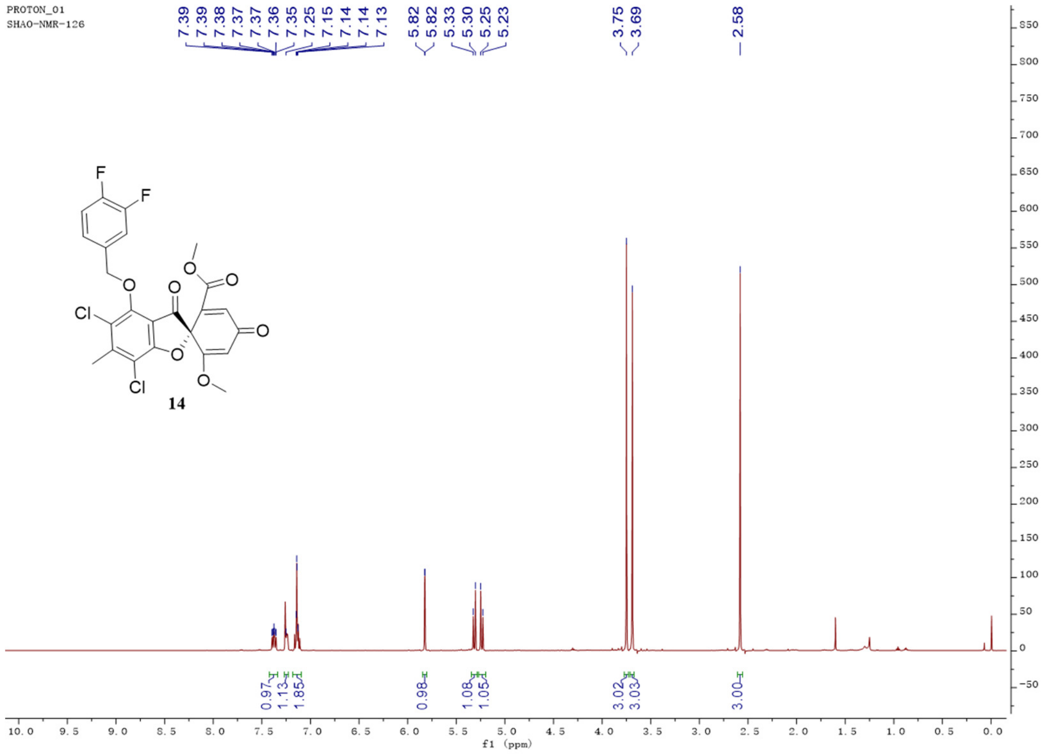

Figure S40. ^{1}H NMR (500 MHz, $CDCl_3$) spectrum of compound **14**

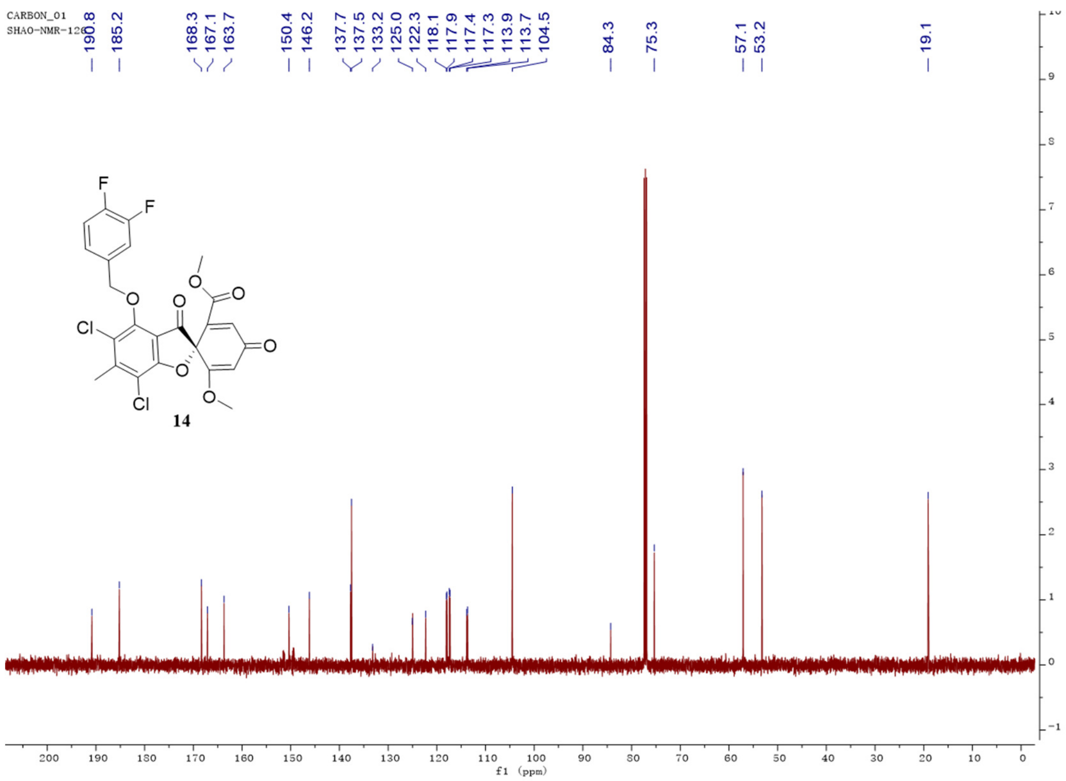

Figure S41. ^{13}C NMR (125 MHz, $CDCl_3$) spectrum of compound **14**

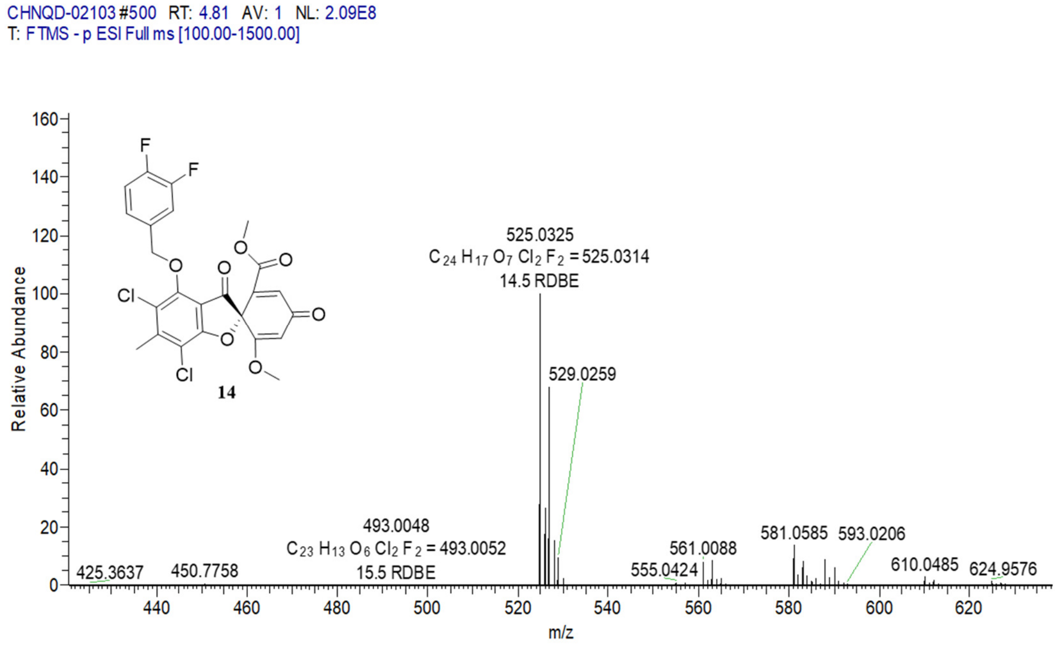

Figure S42. HRESIMS spectrum of compound **14**

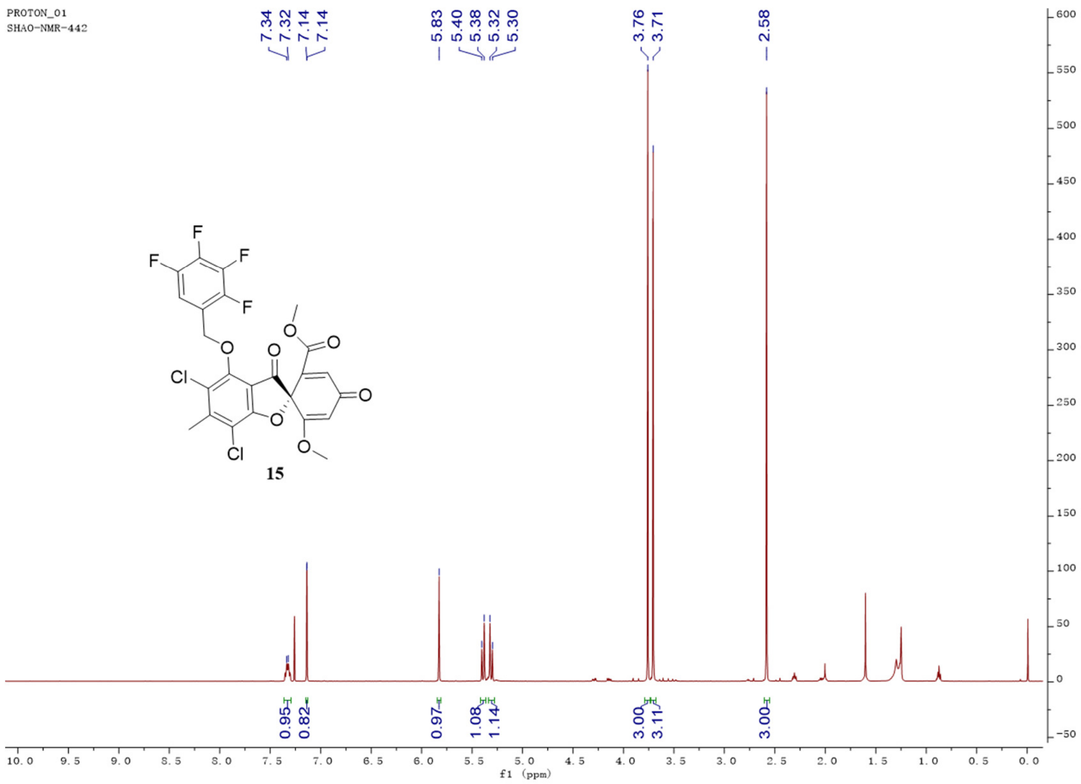

Figure S43. ^{1}H NMR (500 MHz, $CDCl_3$) spectrum of compound **15**

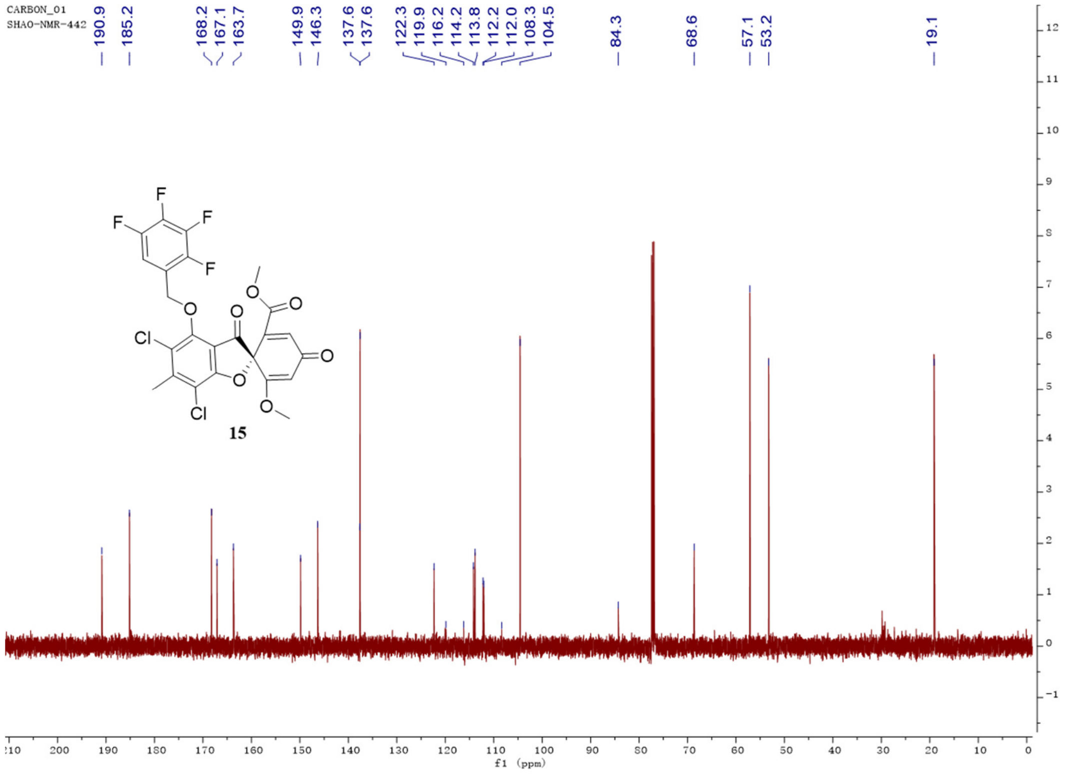

Figure S44. ^{13}C NMR (125 MHz, $CDCl_3$) spectrum of compound **15**

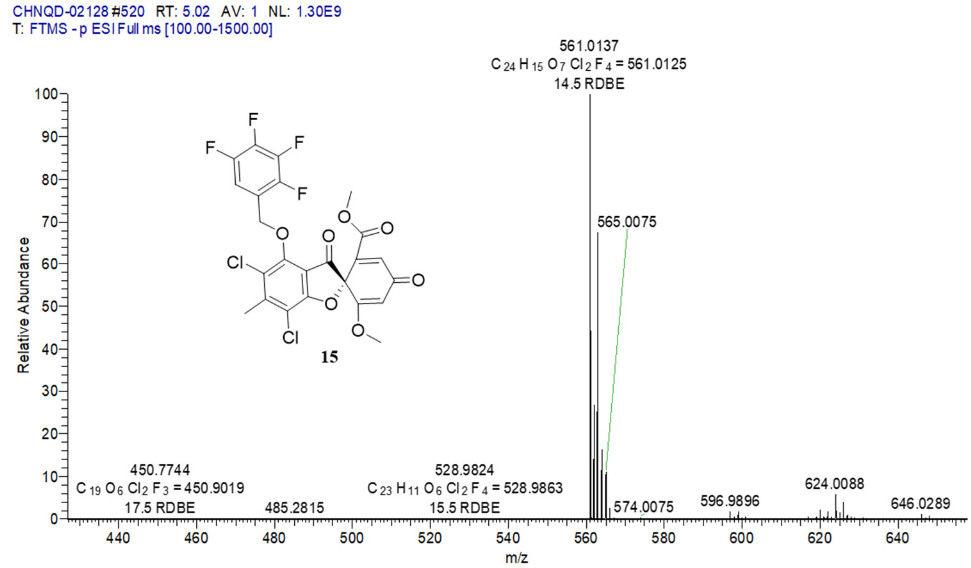

Figure S45. HRESIMS spectrum of compound **15**

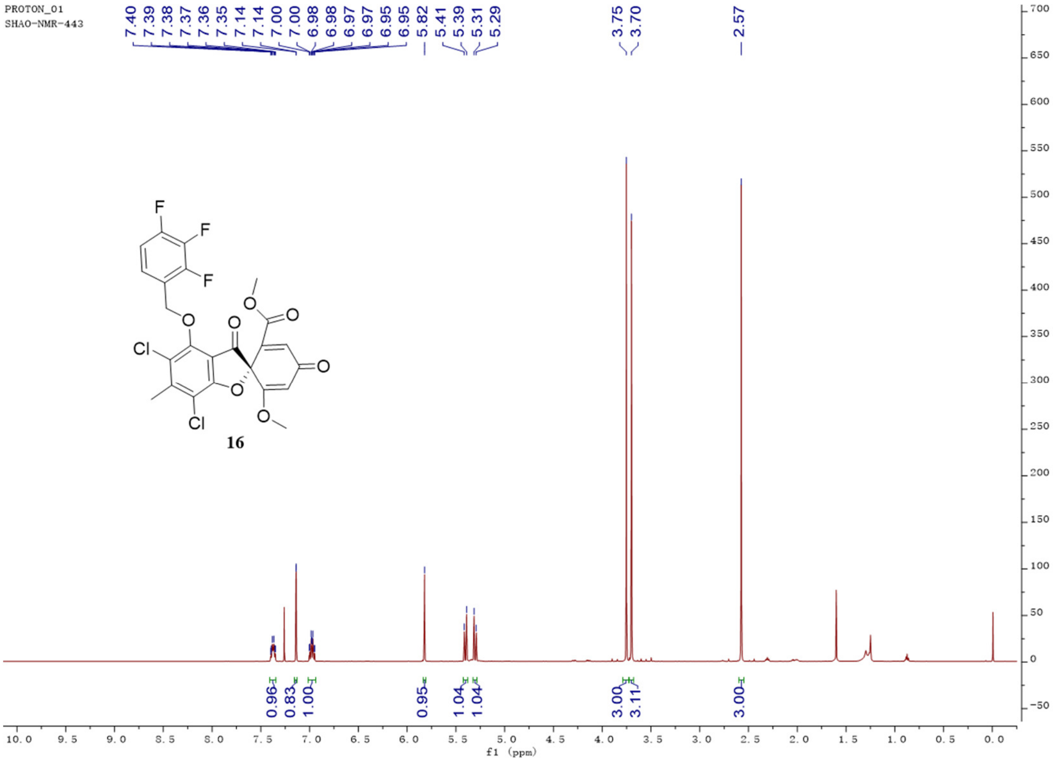

Figure S46. ^{1}H NMR (500 MHz, $CDCl_3$) spectrum of compound **16**

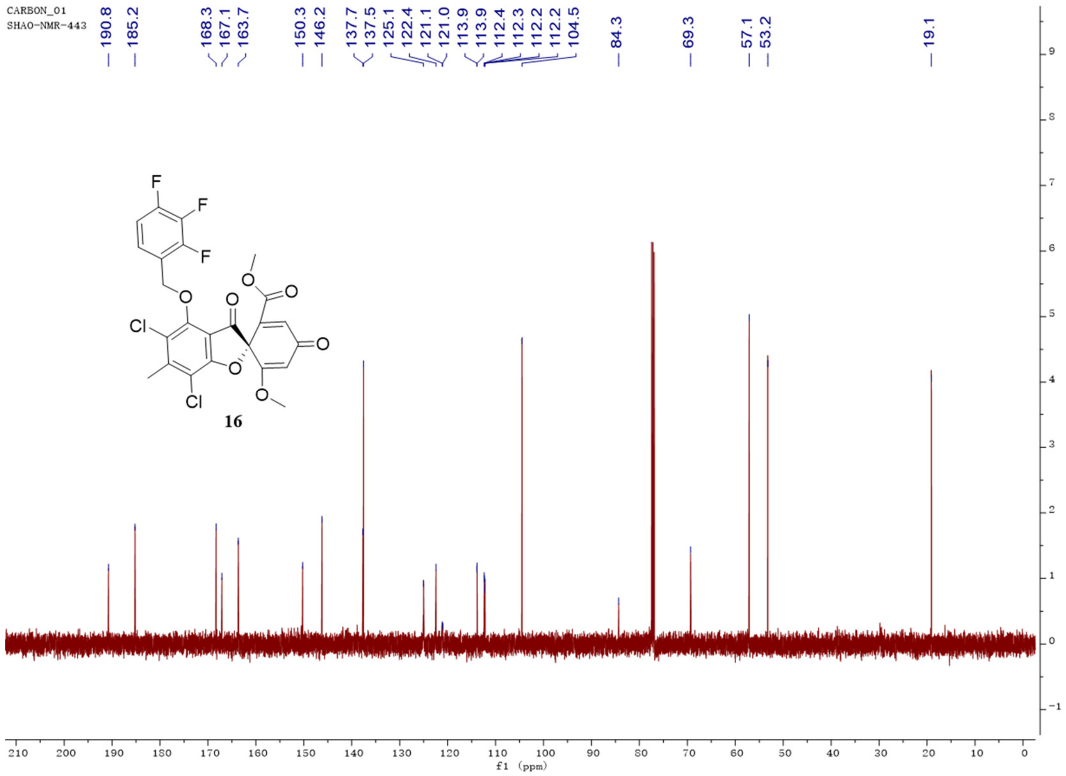

Figure S47. ^{13}C NMR (125 MHz, $CDCl_3$) spectrum of compound **16**

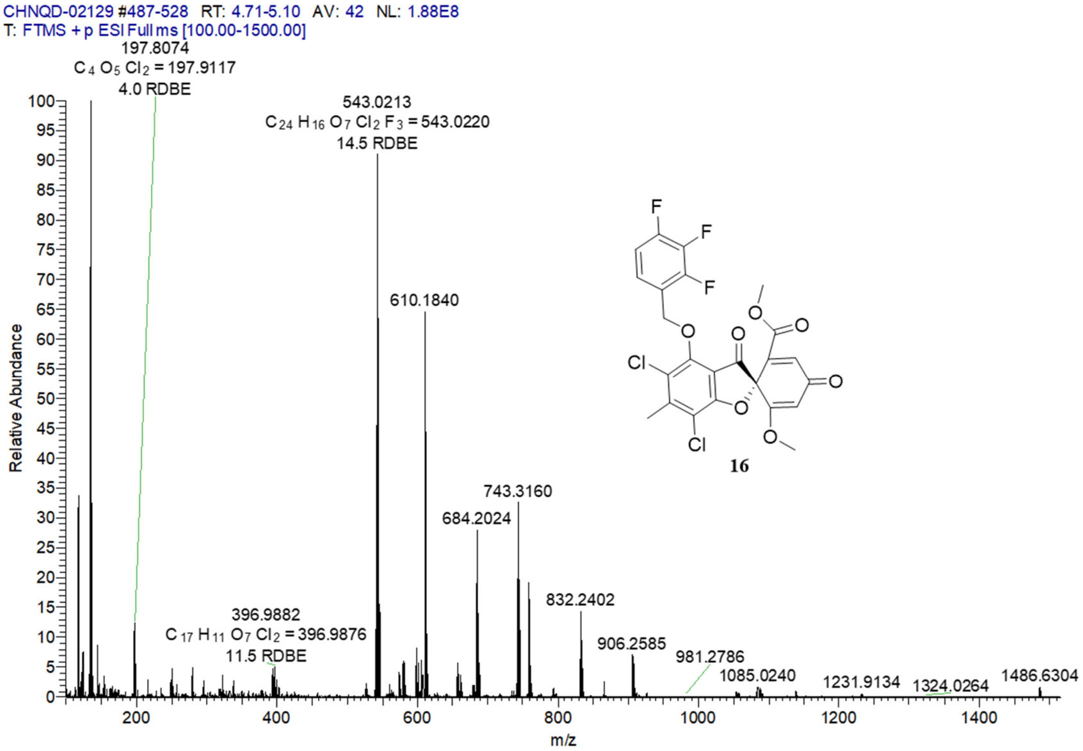

Figure S48. HRESIMS spectrum of compound **16**

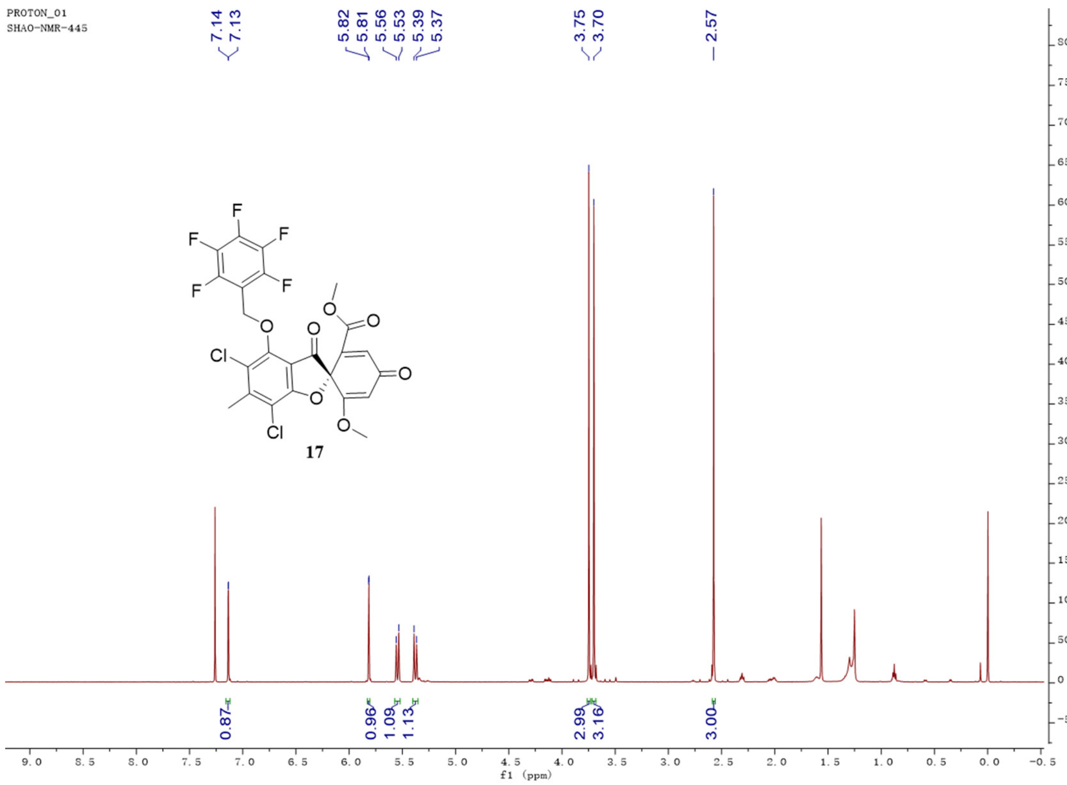

Figure S49. ^{1}H NMR (500 MHz, $CDCl_3$) spectrum of compound **17**

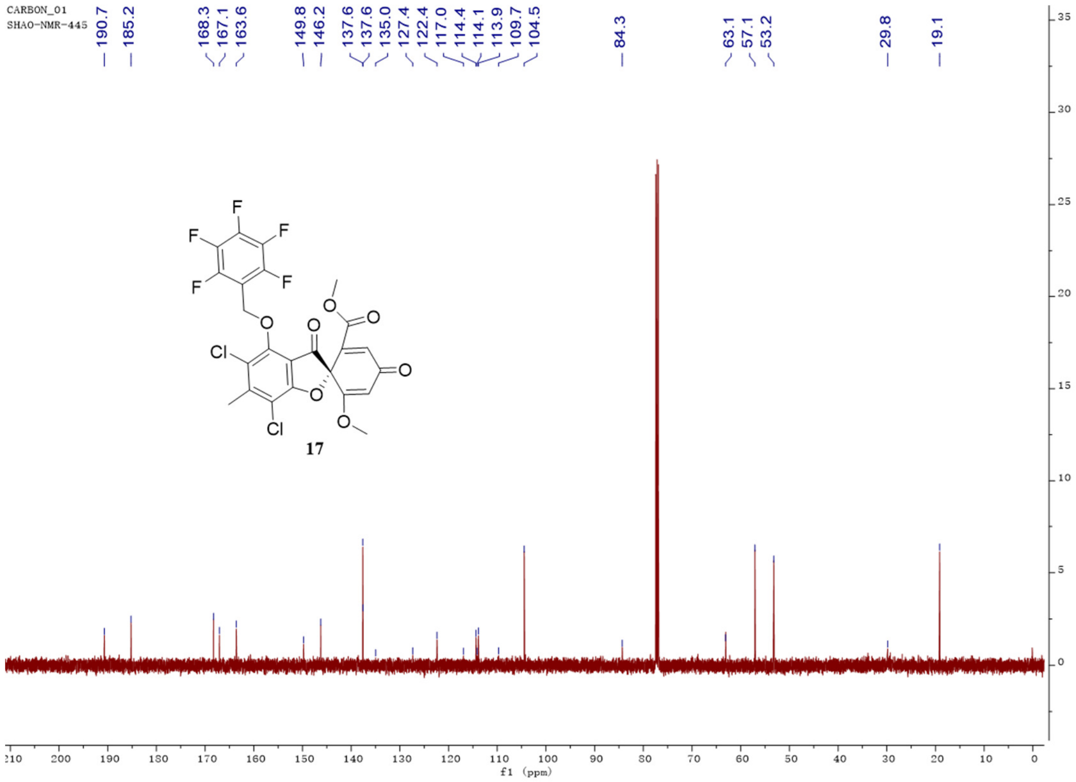

Figure S50. ^{13}C NMR (125 MHz, $CDCl_3$) spectrum of compound **17**

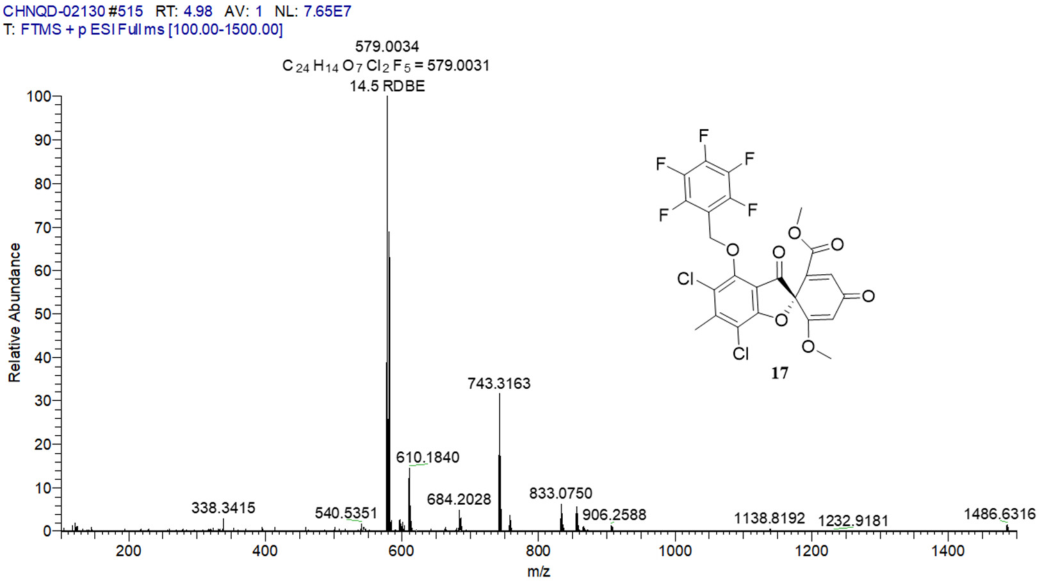

Figure S51. HRESIMS spectrum of compound **17**

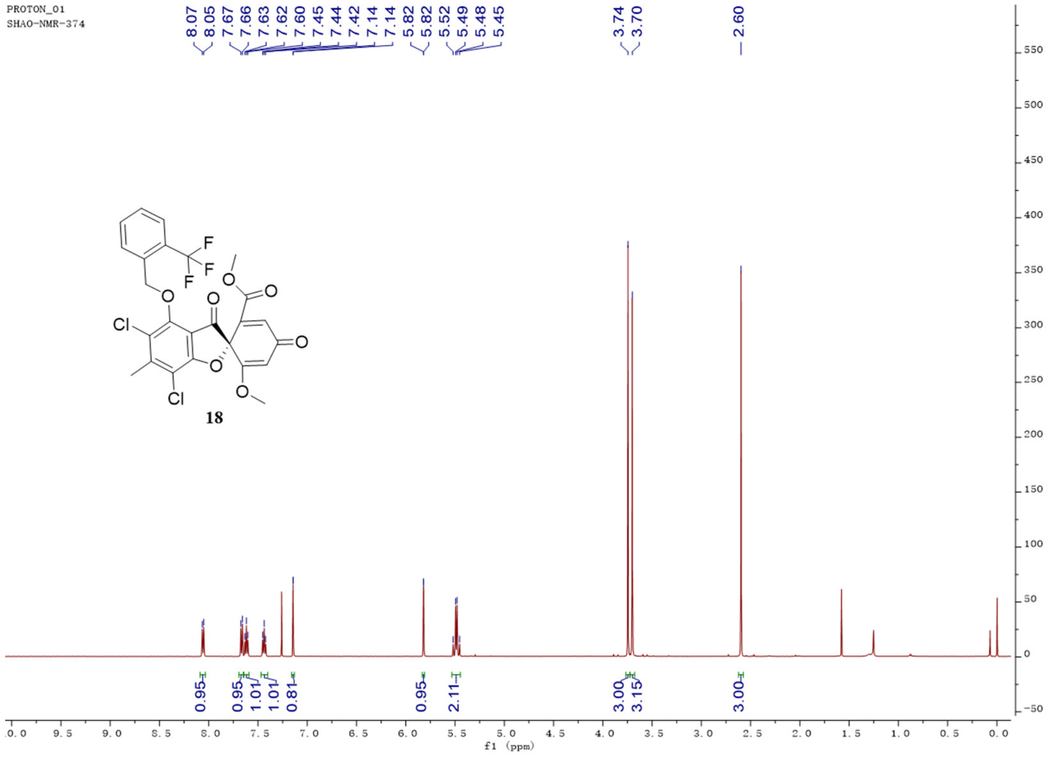

Figure S52. ^{1}H NMR (500 MHz, $CDCl_3$) spectrum of compound **18**

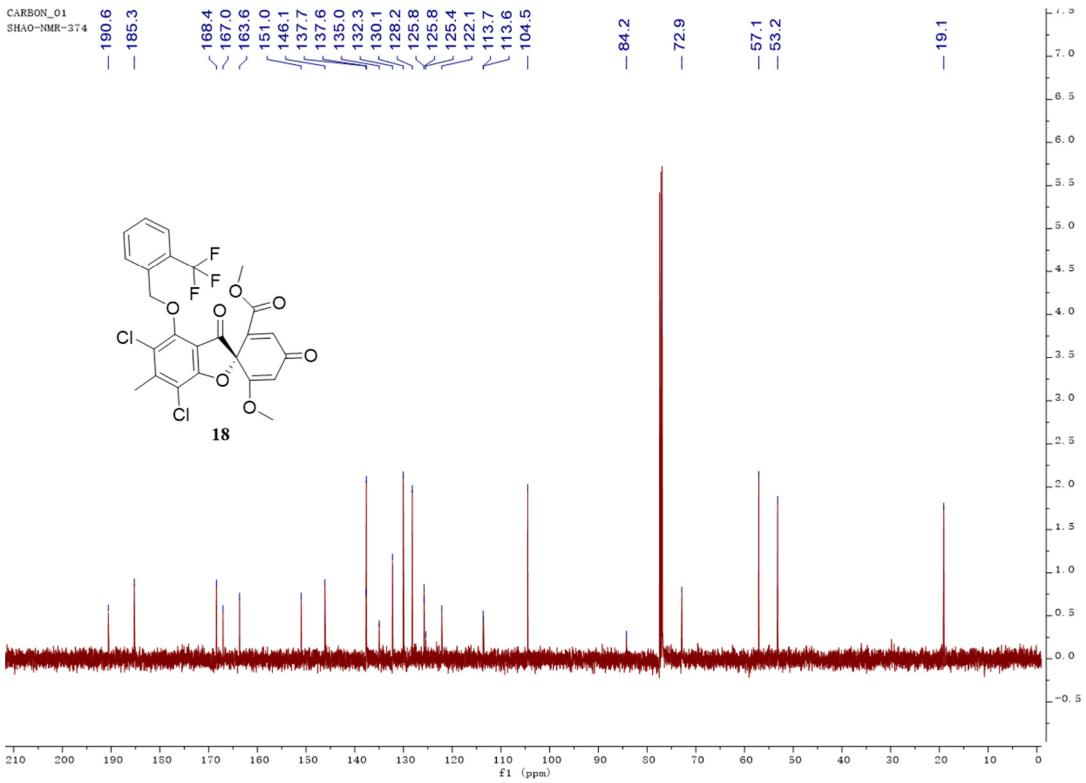

Figure S53. ^{13}C NMR (125 MHz, CDCl$_3$) spectrum of compound **18**

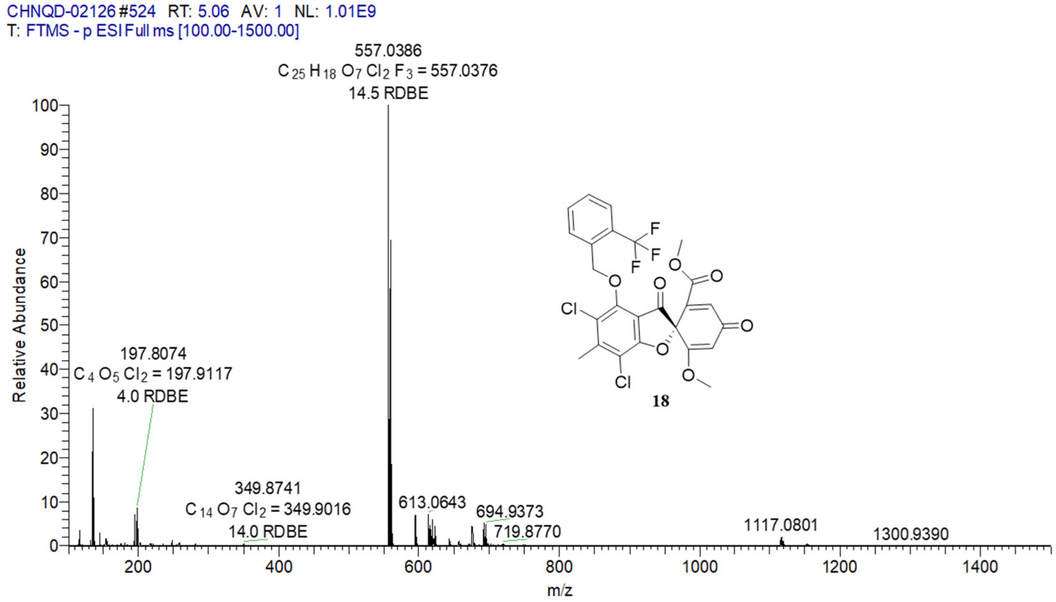

Figure S54. HRESIMS spectrum of compound **18**

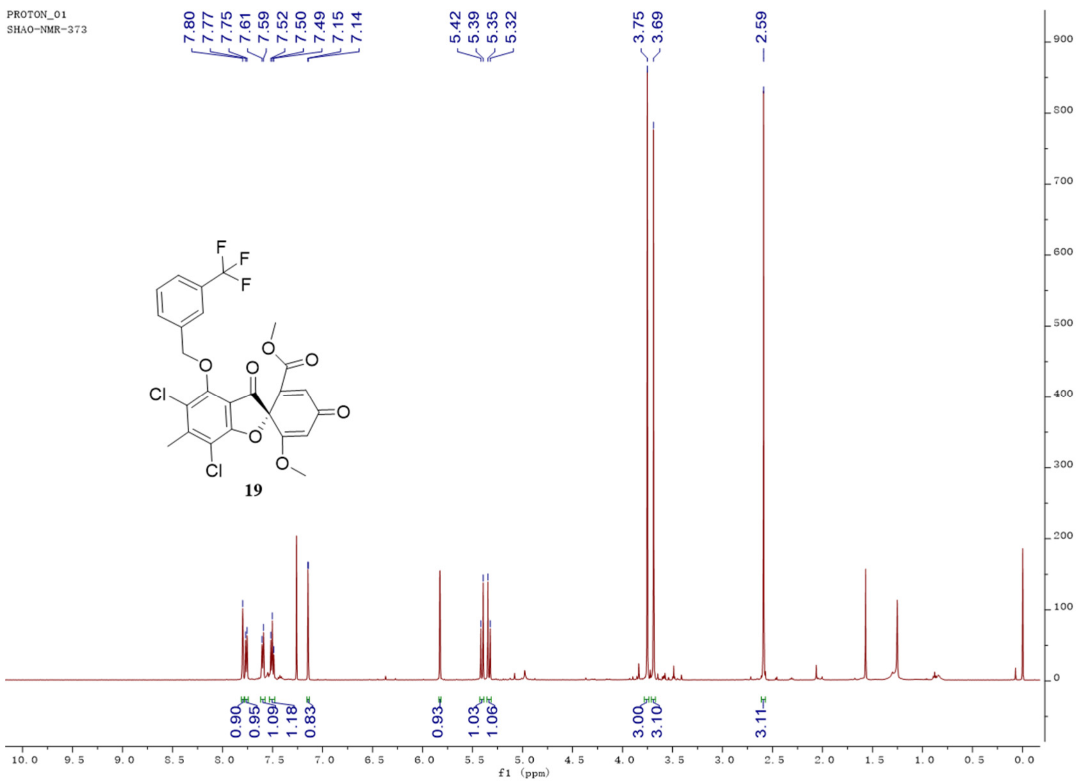

Figure S55. ^{1}H NMR (500 MHz, $CDCl_3$) spectrum of compound **19**

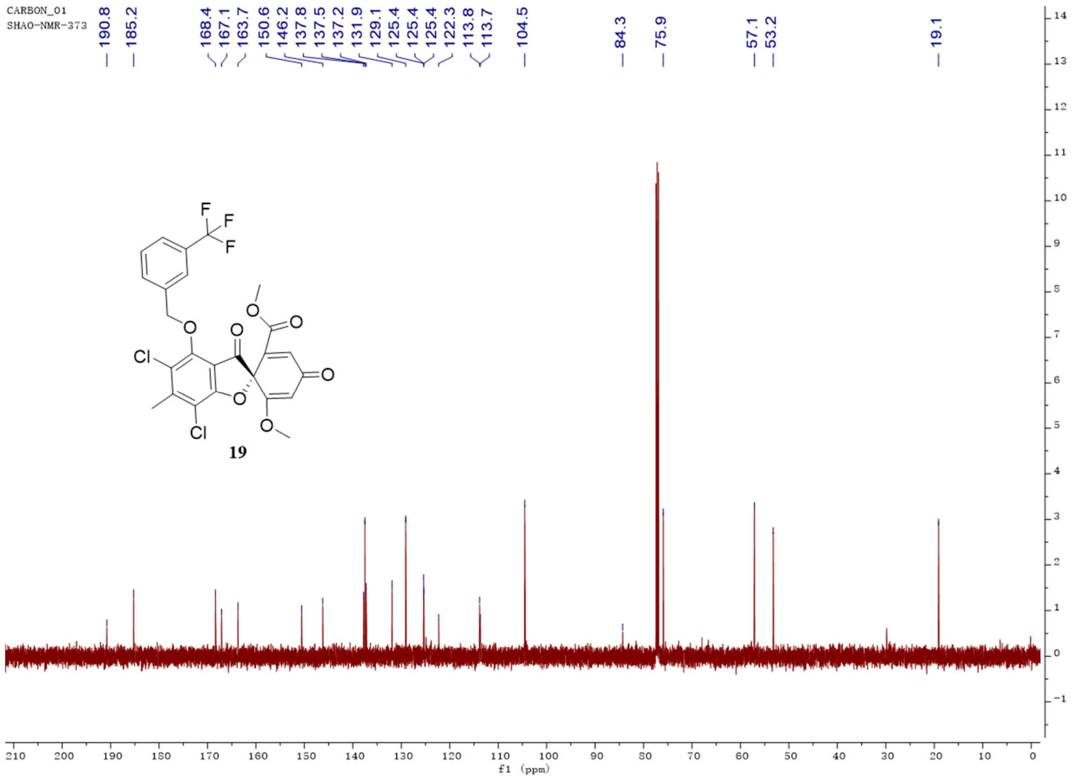

Figure S56. ^{13}C NMR (125 MHz, $CDCl_3$) spectrum of compound **19**

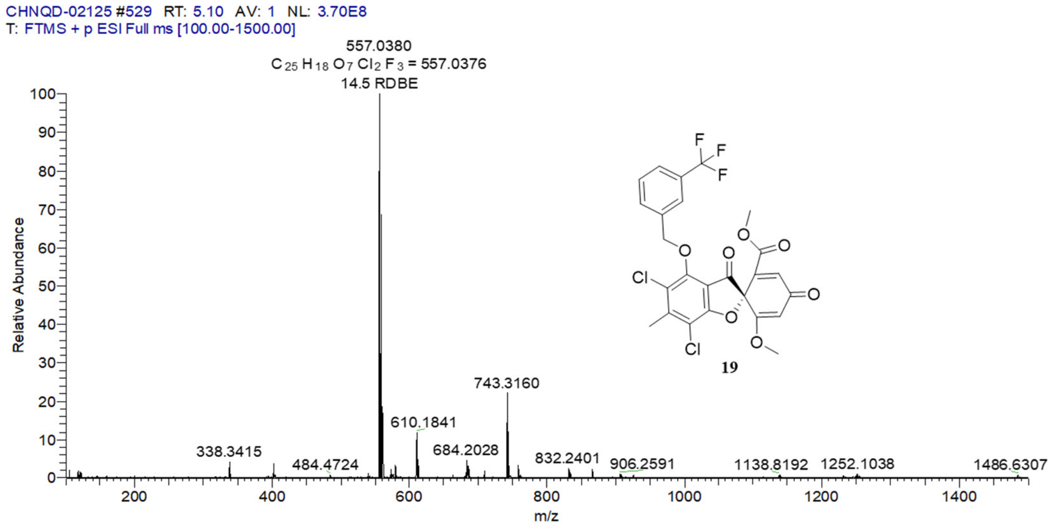

Figure S57. HRESIMS spectrum of compound **19**

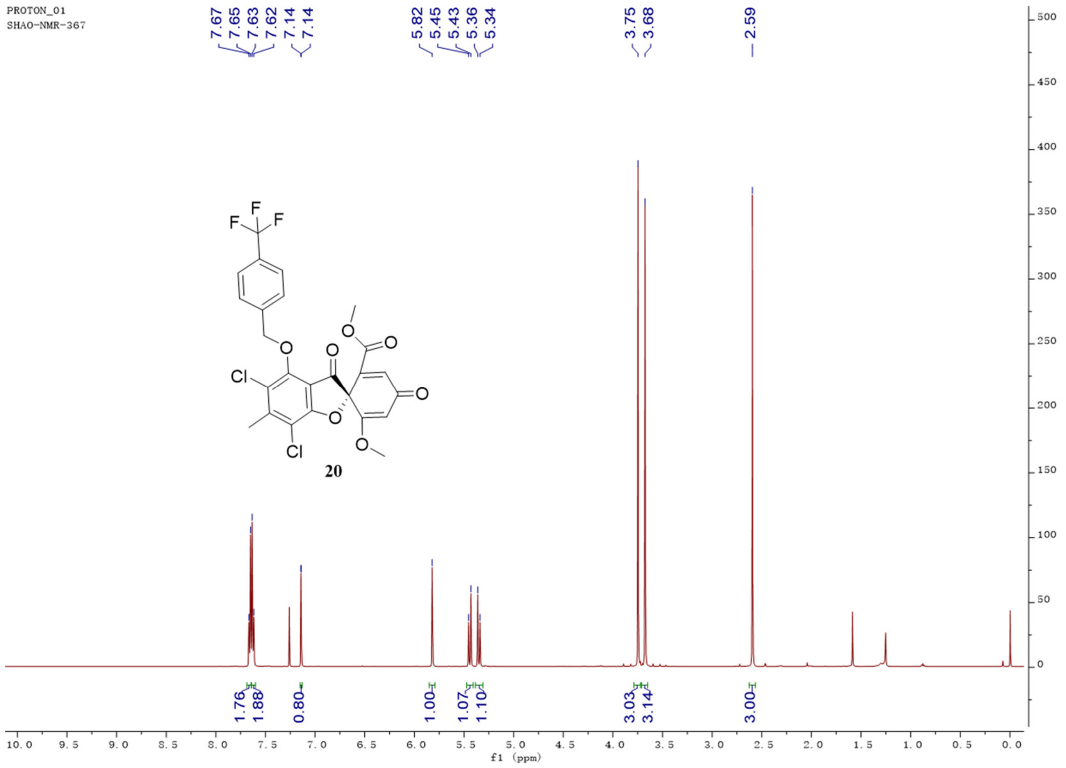

Figure S58. ¹H NMR (500 MHz, $CDCl_3$) spectrum of compound **20**

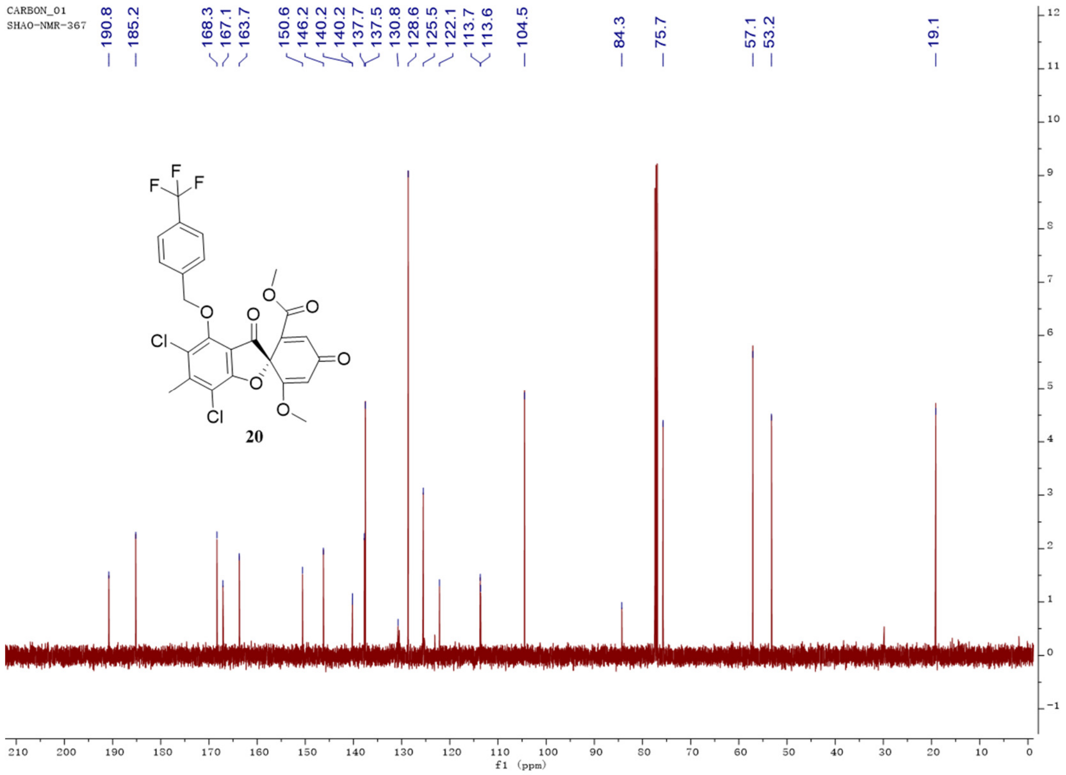

Figure S59. ^{13}C NMR (125 MHz, $CDCl_3$) spectrum of compound **20**

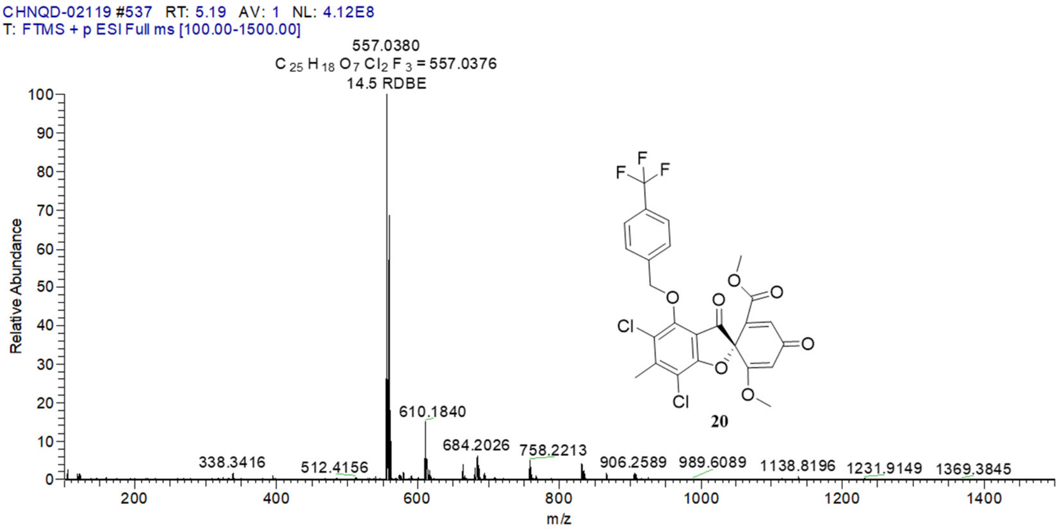

Figure S60. HRESIMS spectrum of compound **20**

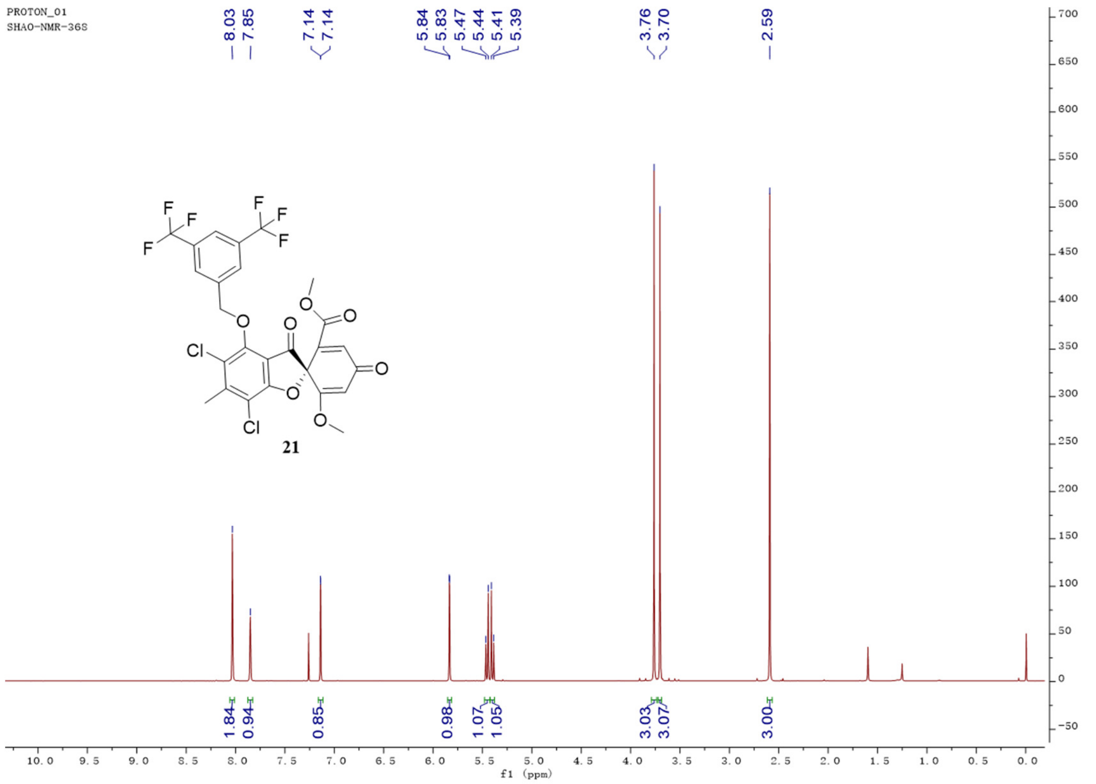

Figure S61. ^{1}H NMR (500 MHz, $CDCl_3$) spectrum of compound **21**

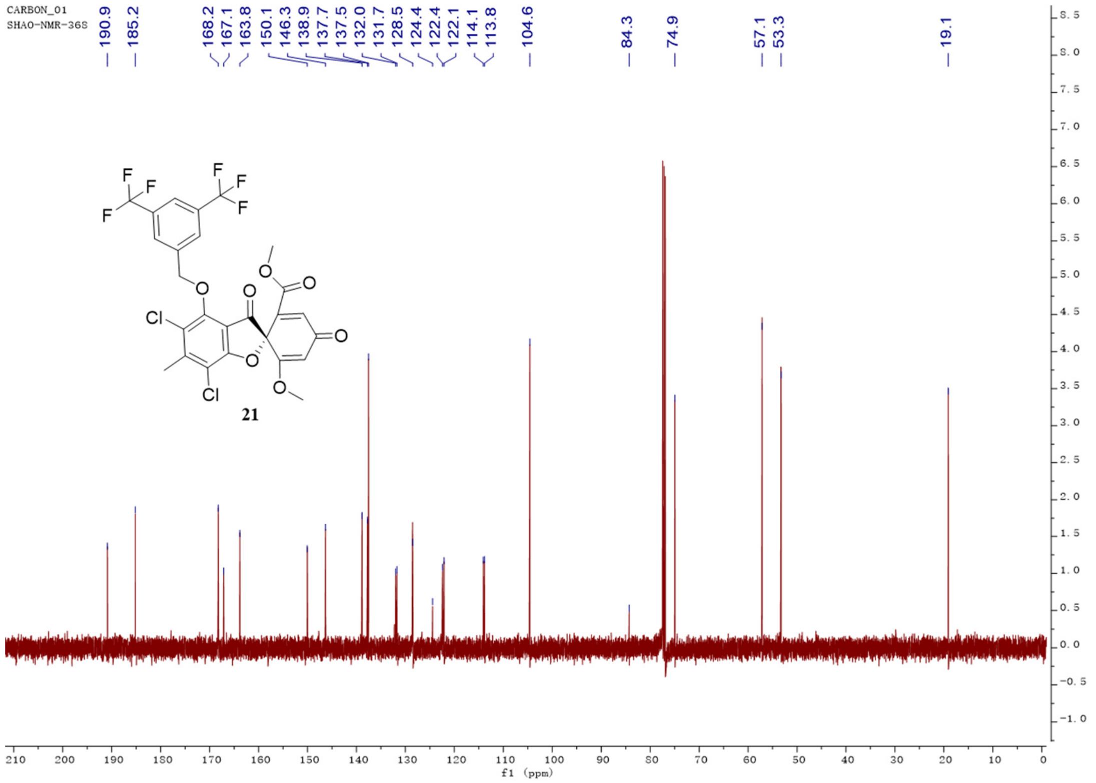

Figure S62. ^{13}C NMR (125 MHz, $CDCl_3$) spectrum of compound **21**

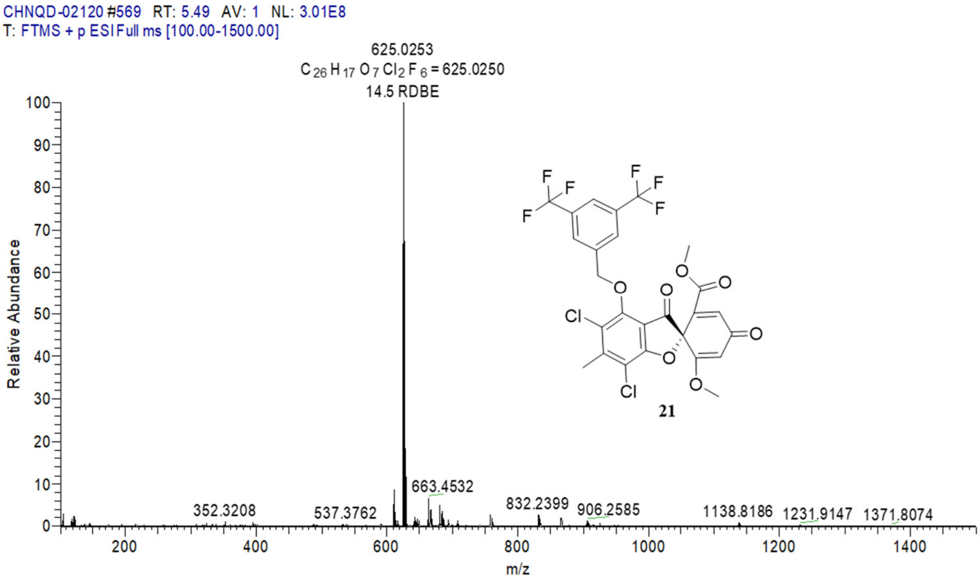

Figure S63. HRESIMS spectrum of compound **21**

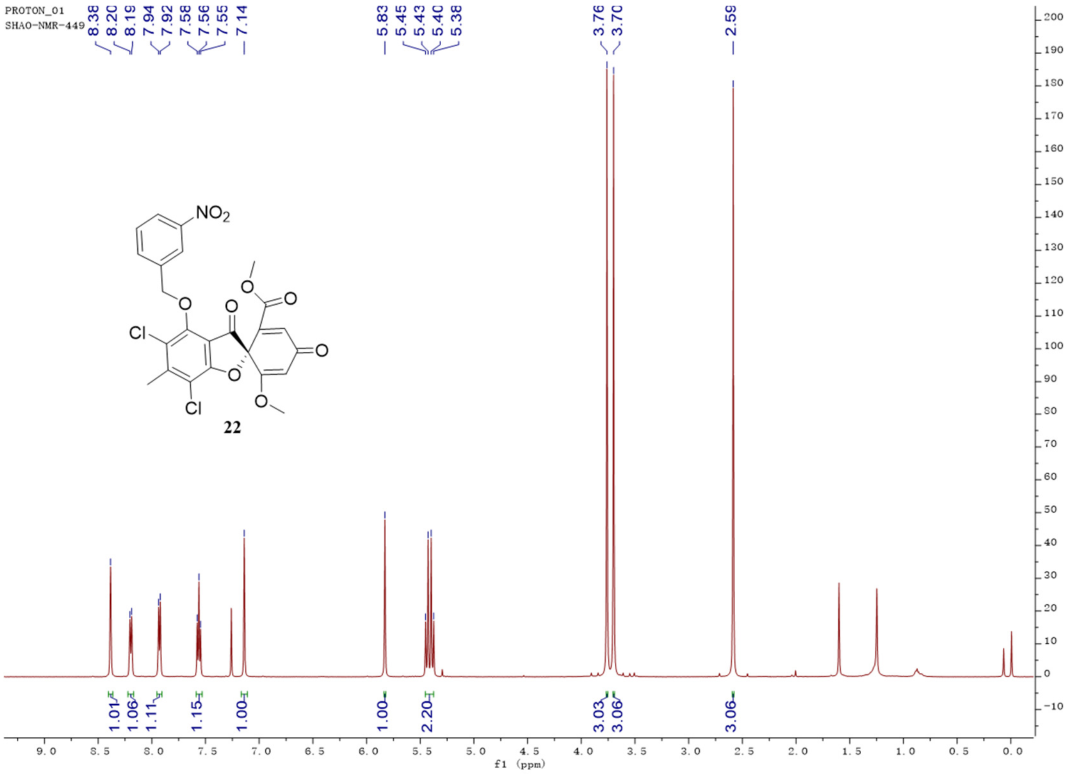

Figure S64. ^{1}H NMR (500 MHz, $CDCl_3$) spectrum of compound **22**

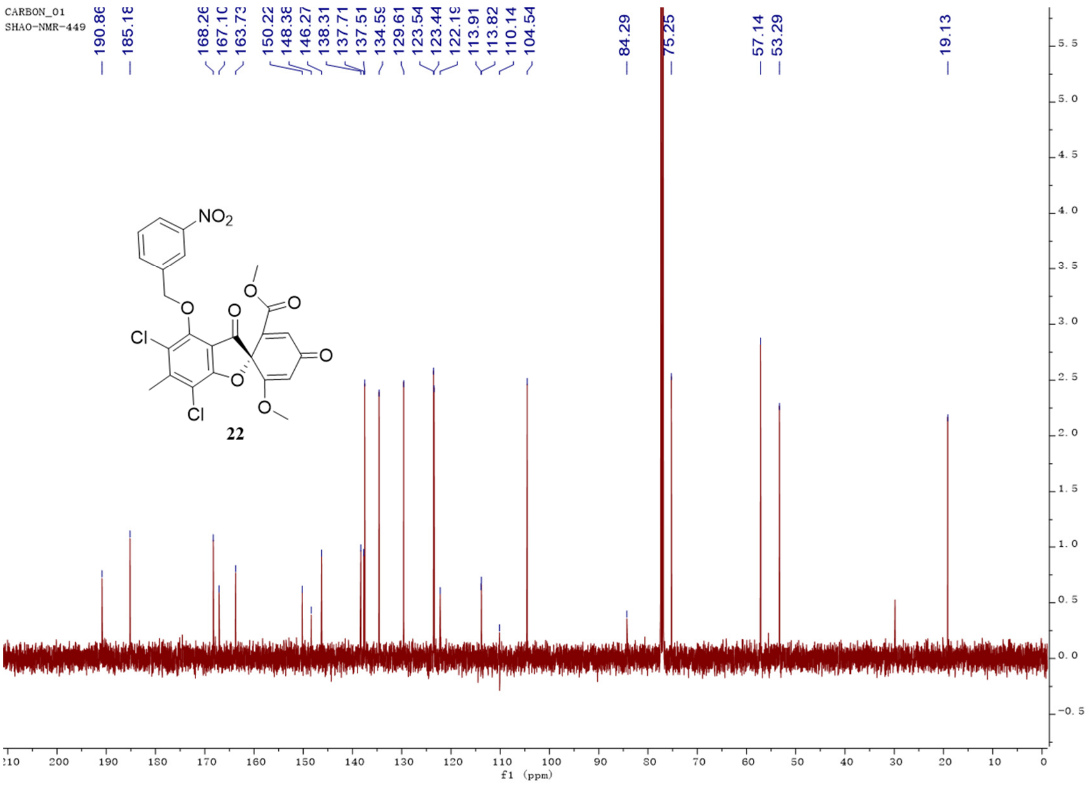

Figure S65. ^{13}C NMR (125 MHz, $CDCl_3$) spectrum of compound **22**

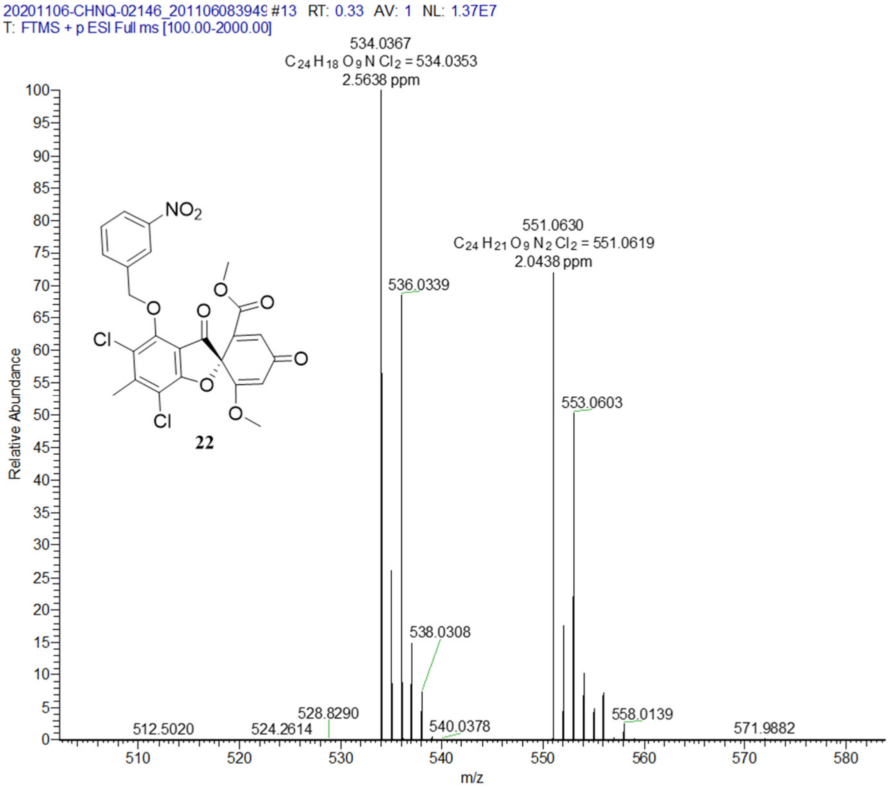

Figure S66. HRESIMS spectrum of compound **22**

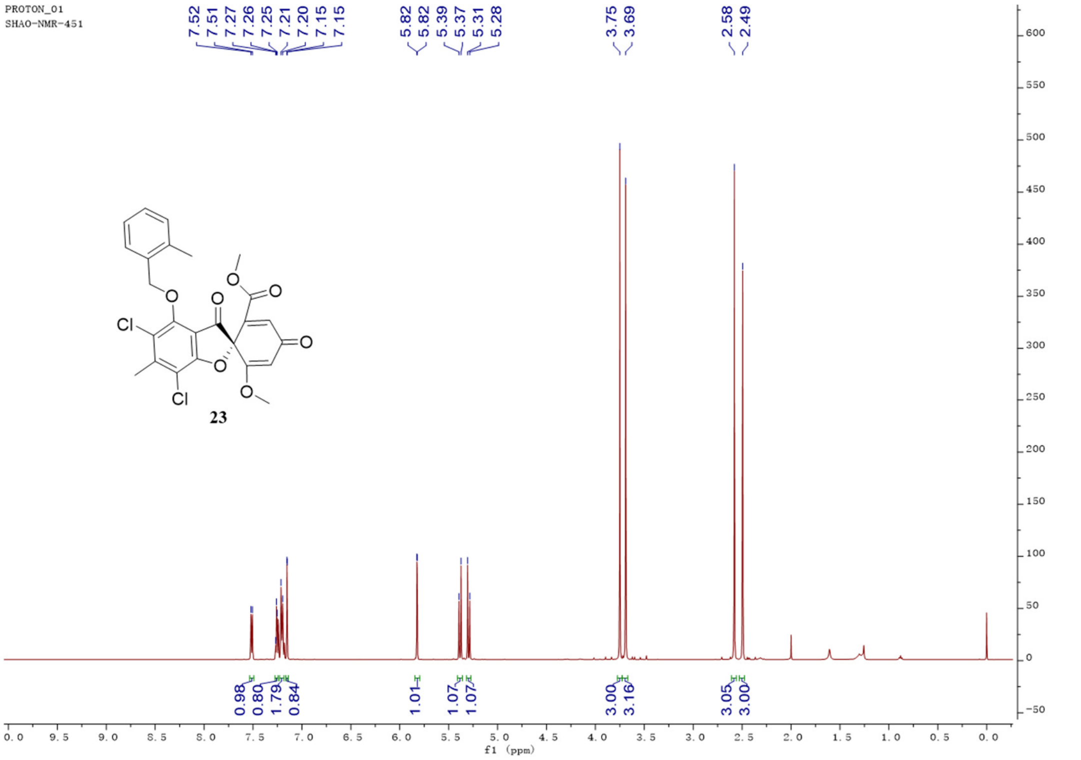

Figure S67. ^{1}H NMR (500 MHz, $CDCl_3$) spectrum of compound **23**

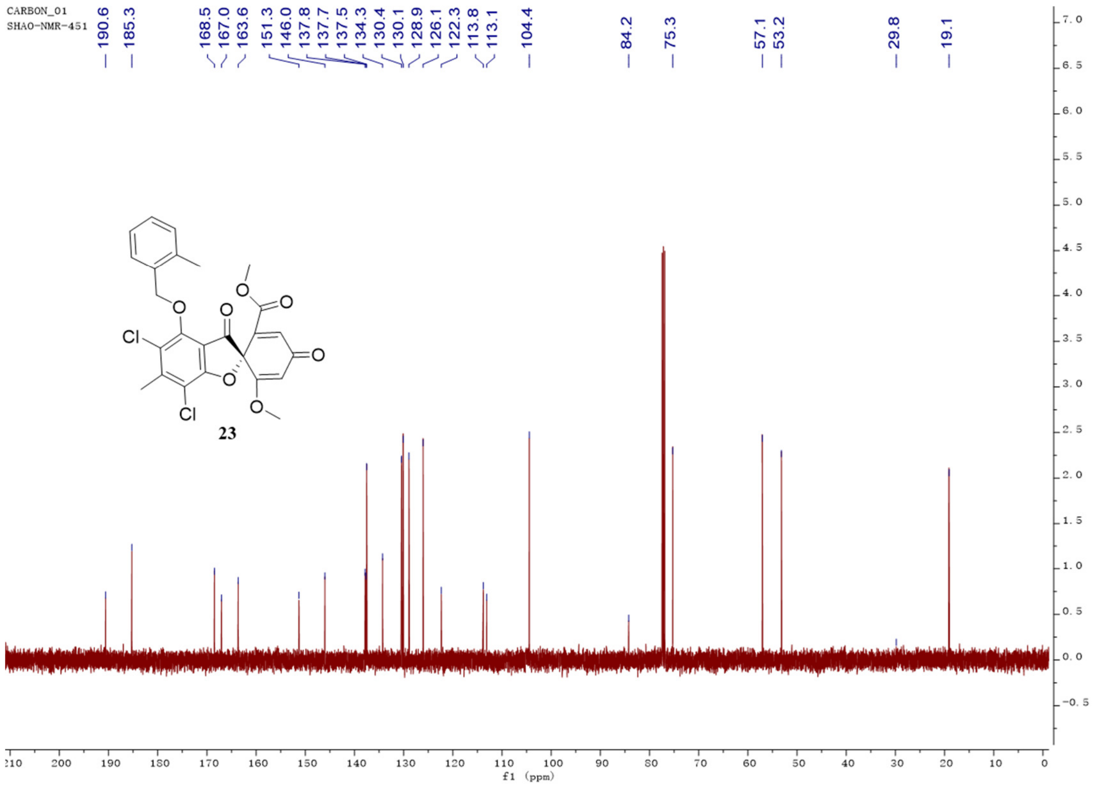

Figure S68. ^{13}C NMR (125 MHz, $CDCl_3$) spectrum of compound **23**

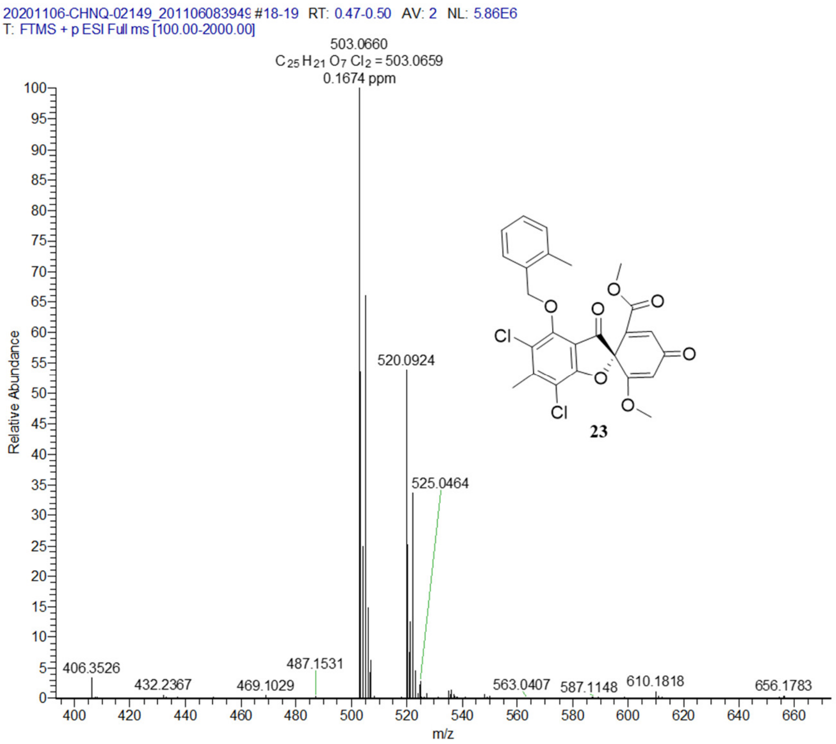

Figure S69. HRESIMS spectrum of compound **23**

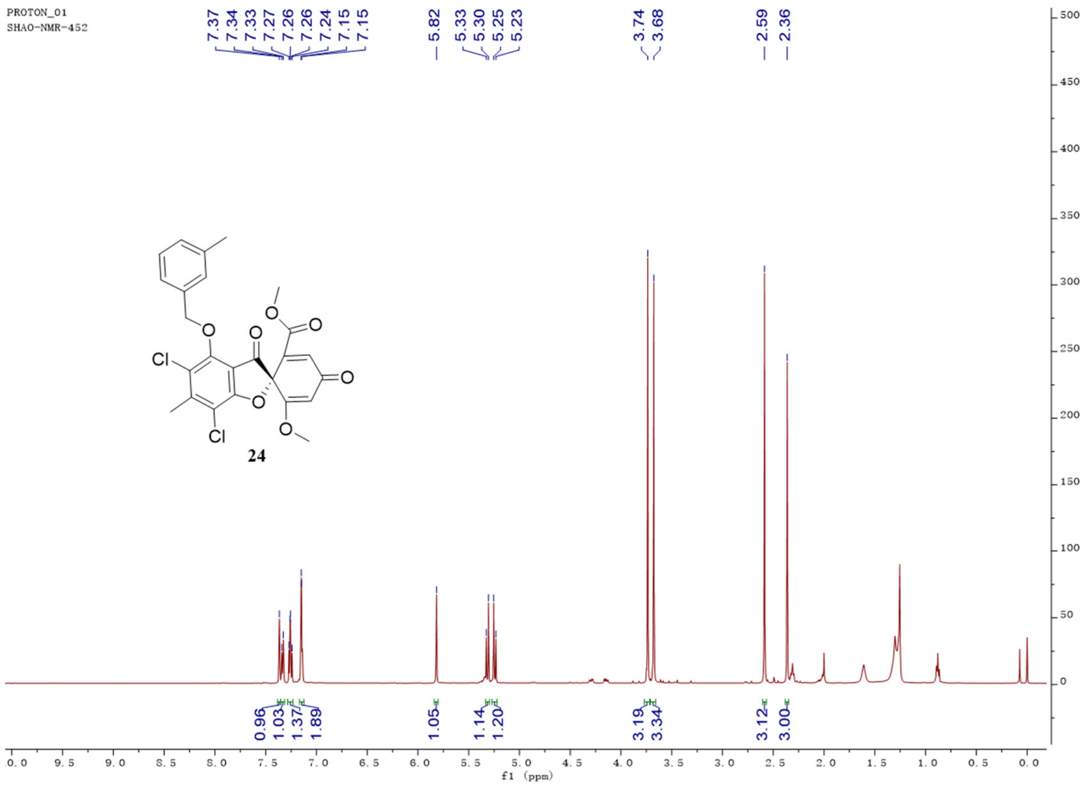

Figure S70. ^{1}H NMR (500 MHz, $CDCl_3$) spectrum of compound **24**

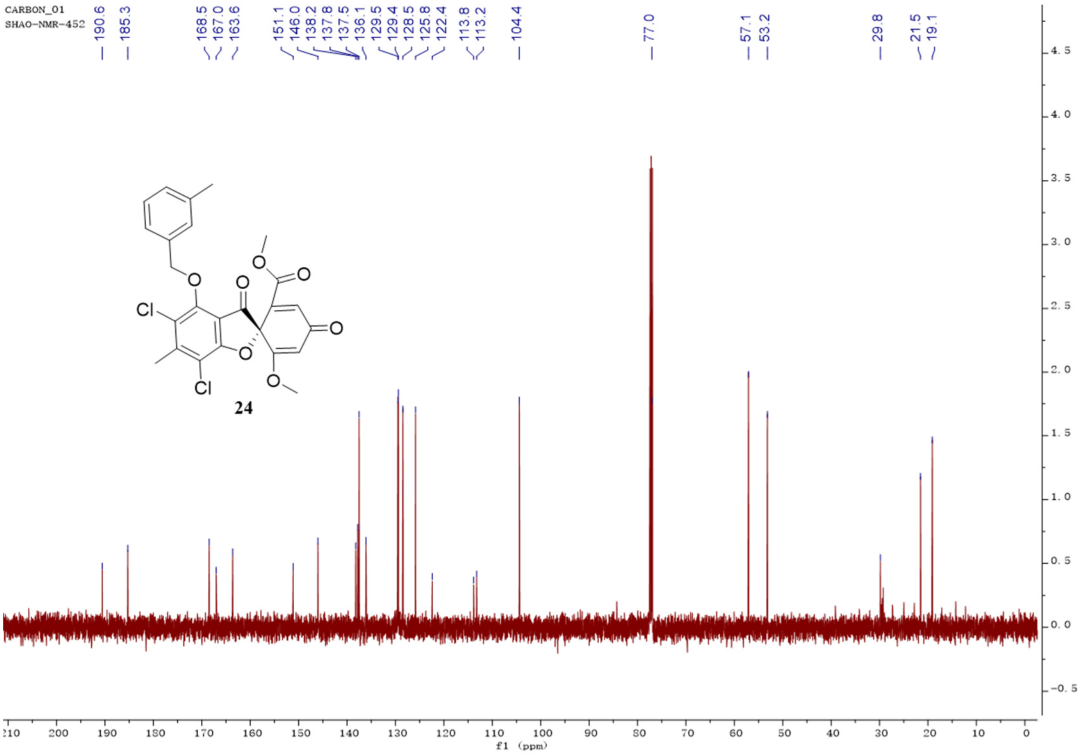

Figure S71. ^{13}C NMR (125 MHz, $CDCl_3$) spectrum of compound **24**

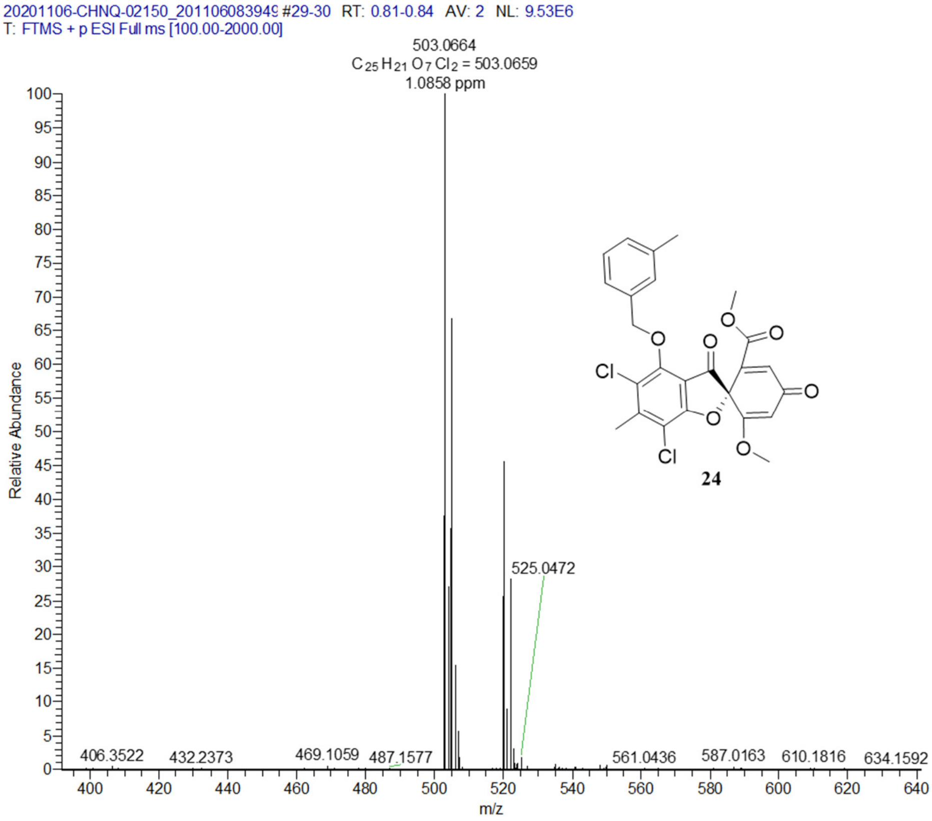

Figure S72. HRESIMS spectrum of compound **24**

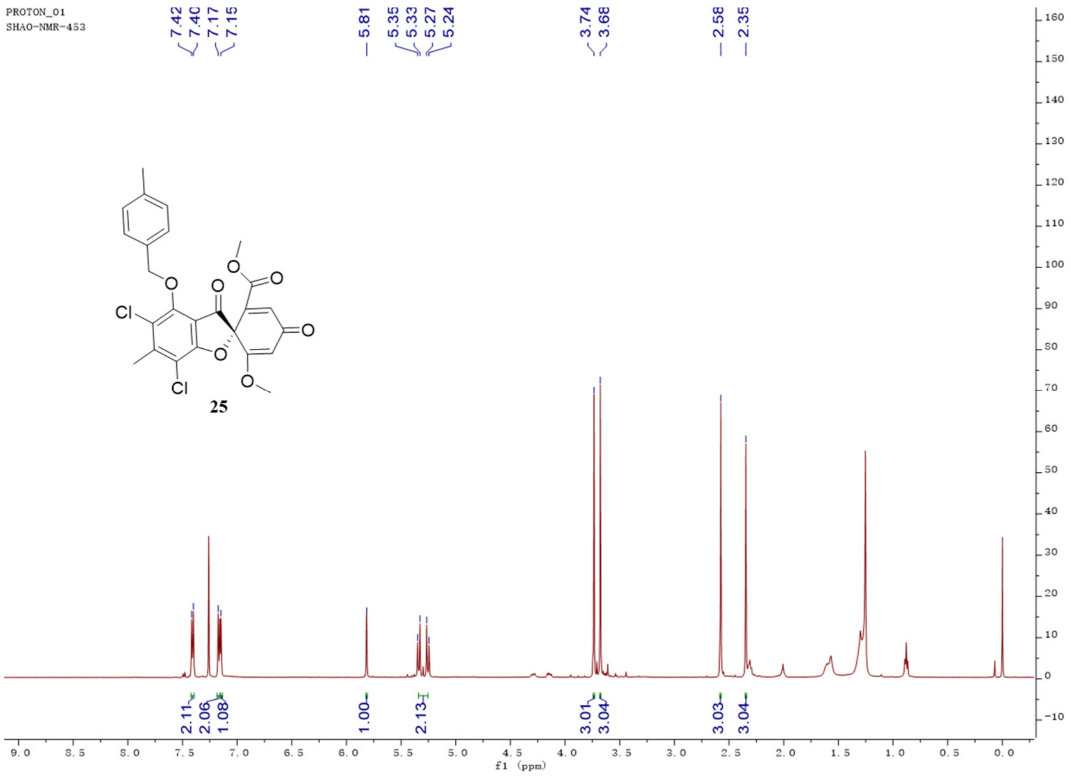

Figure S73. ^{1}H NMR (500 MHz, $CDCl_3$) spectrum of compound **25**

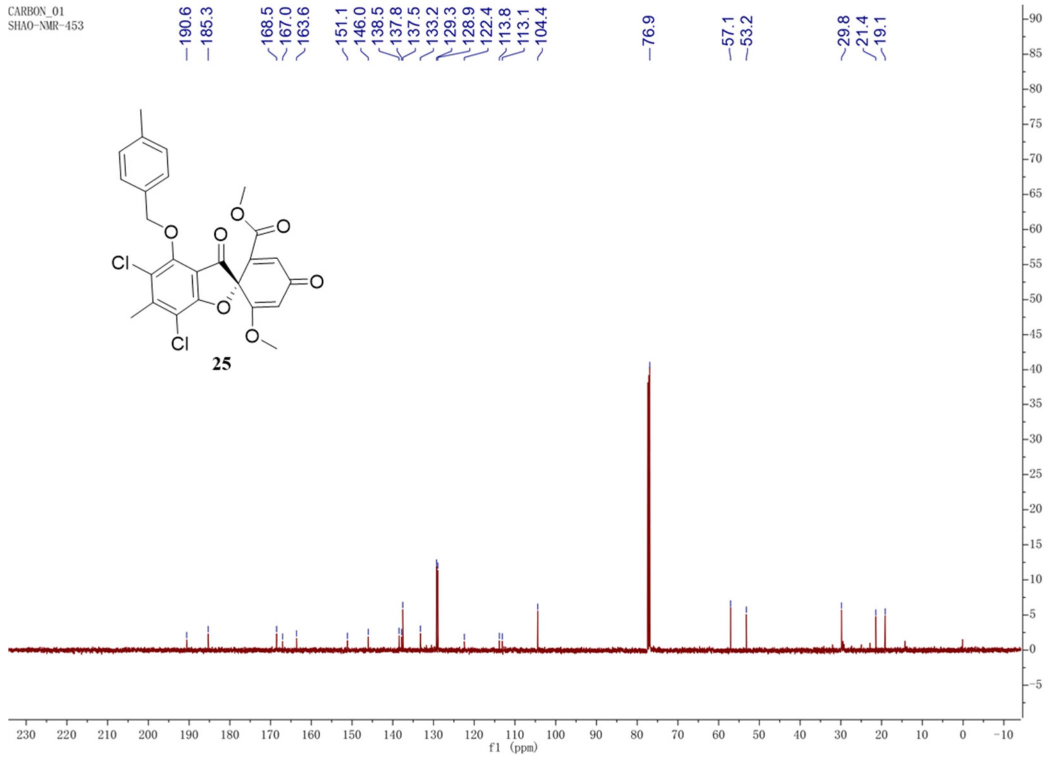

Figure S74. ^{13}C NMR (125 MHz, $CDCl_3$) spectrum of compound **25**

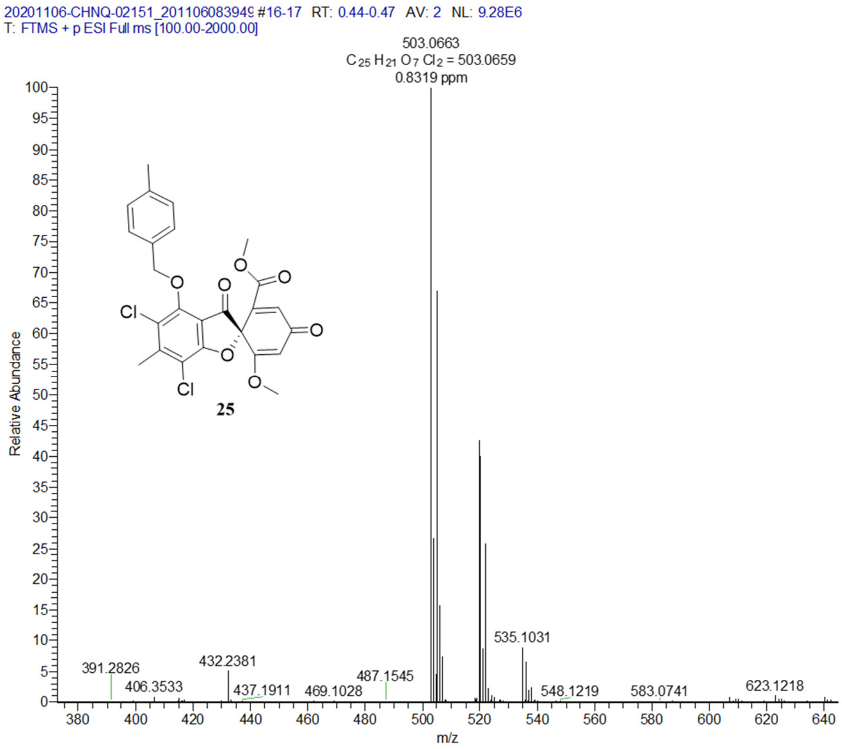

Figure S75. HRESIMS spectrum of compound **25**

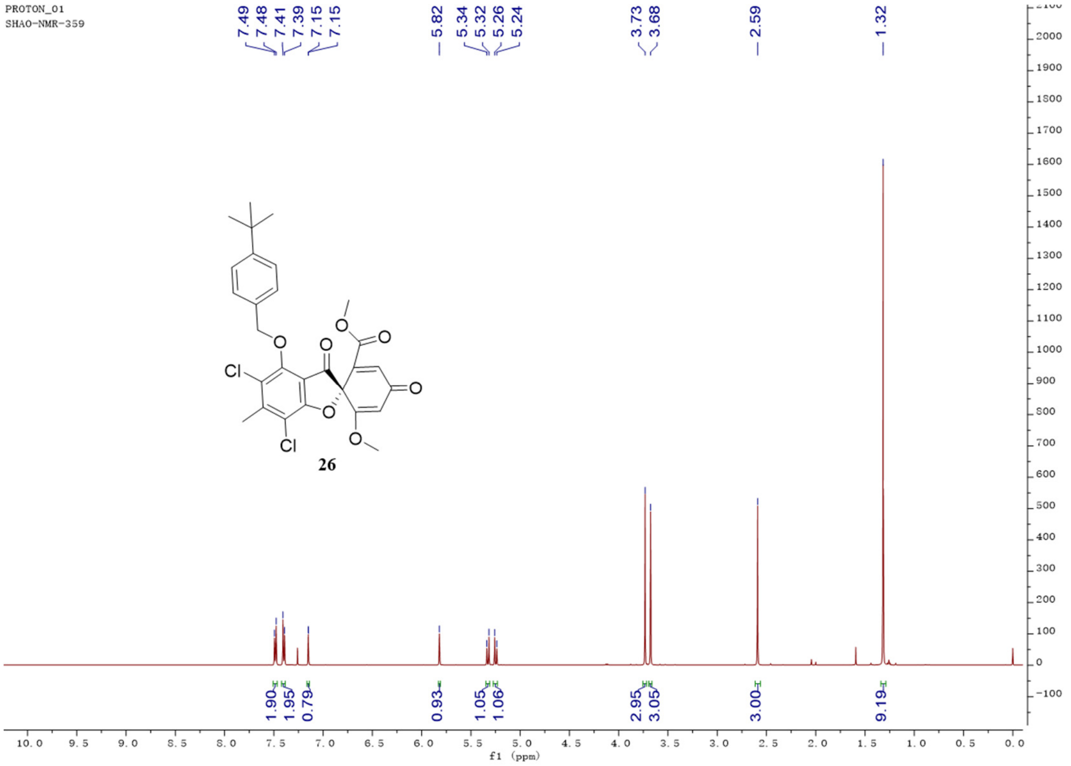

Figure S76. ^{1}H NMR (500 MHz, $CDCl_3$) spectrum of compound **26**

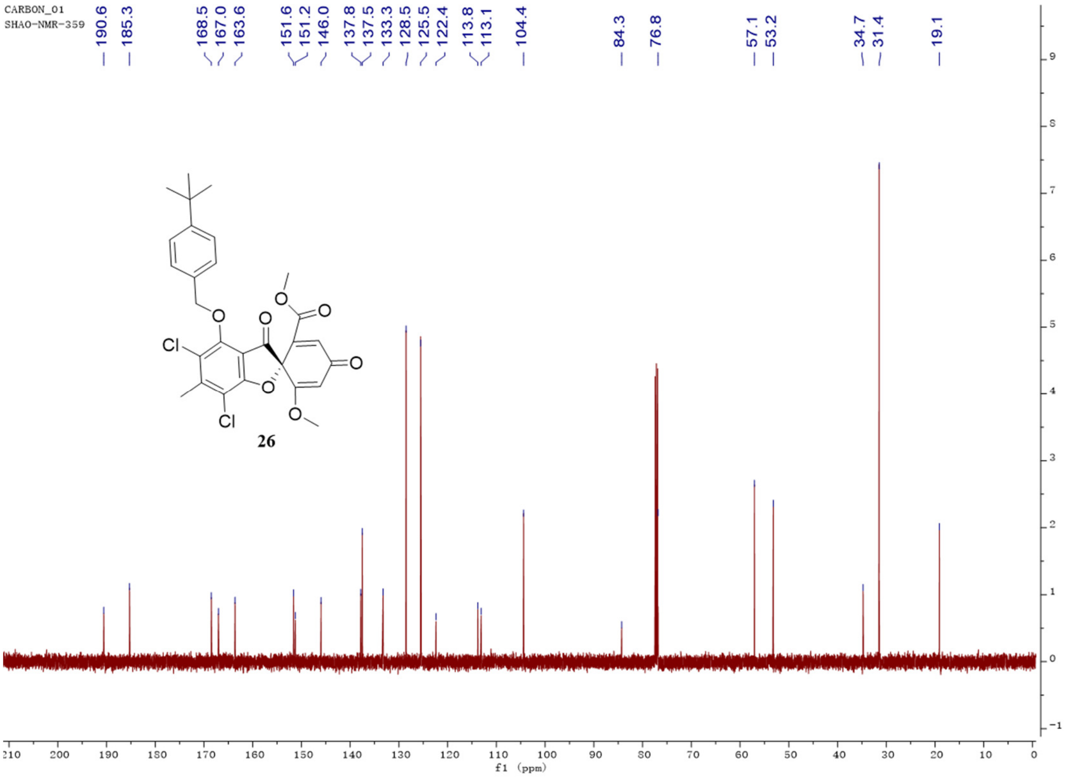

Figure S77. ^{13}C NMR (125 MHz, $CDCl_3$) spectrum of compound **26**

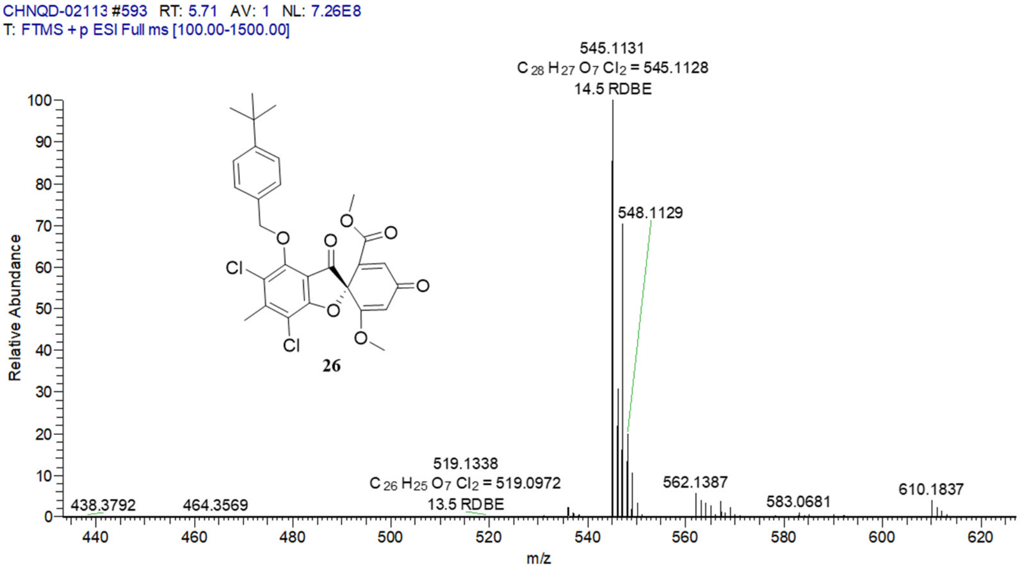

Figure S78. HRESIMS spectrum of compound **26**

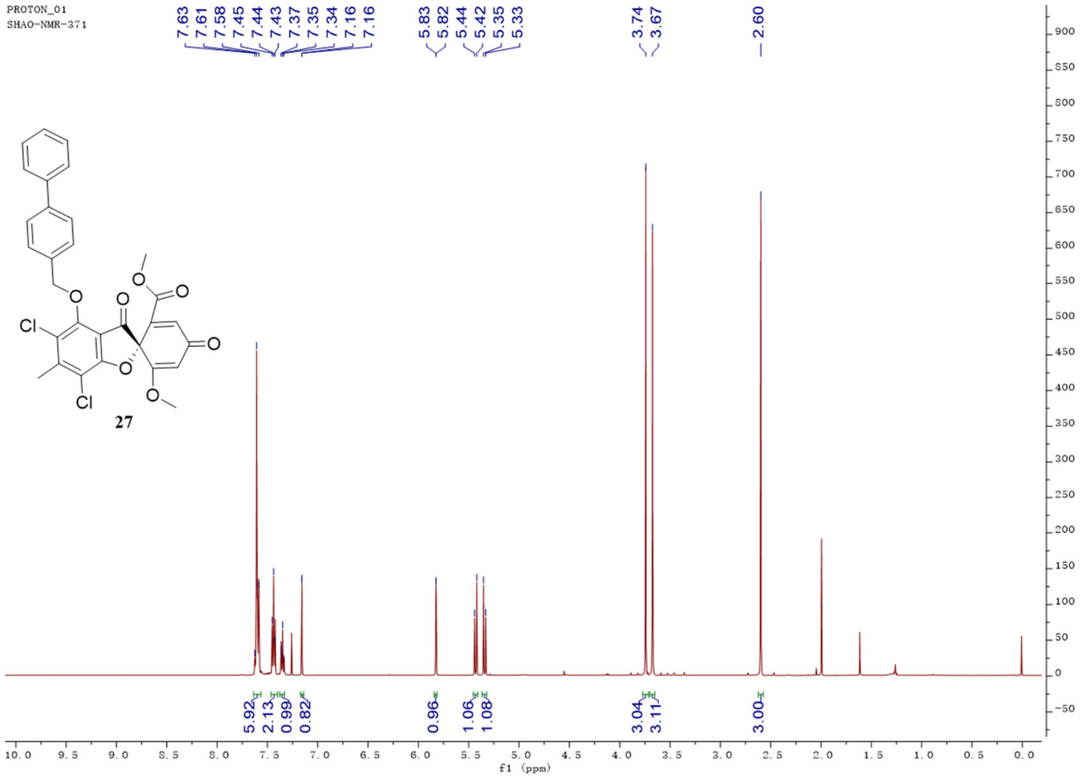

Figure S79. ^{1}H NMR (500 MHz, $CDCl_3$) spectrum of compound **27**

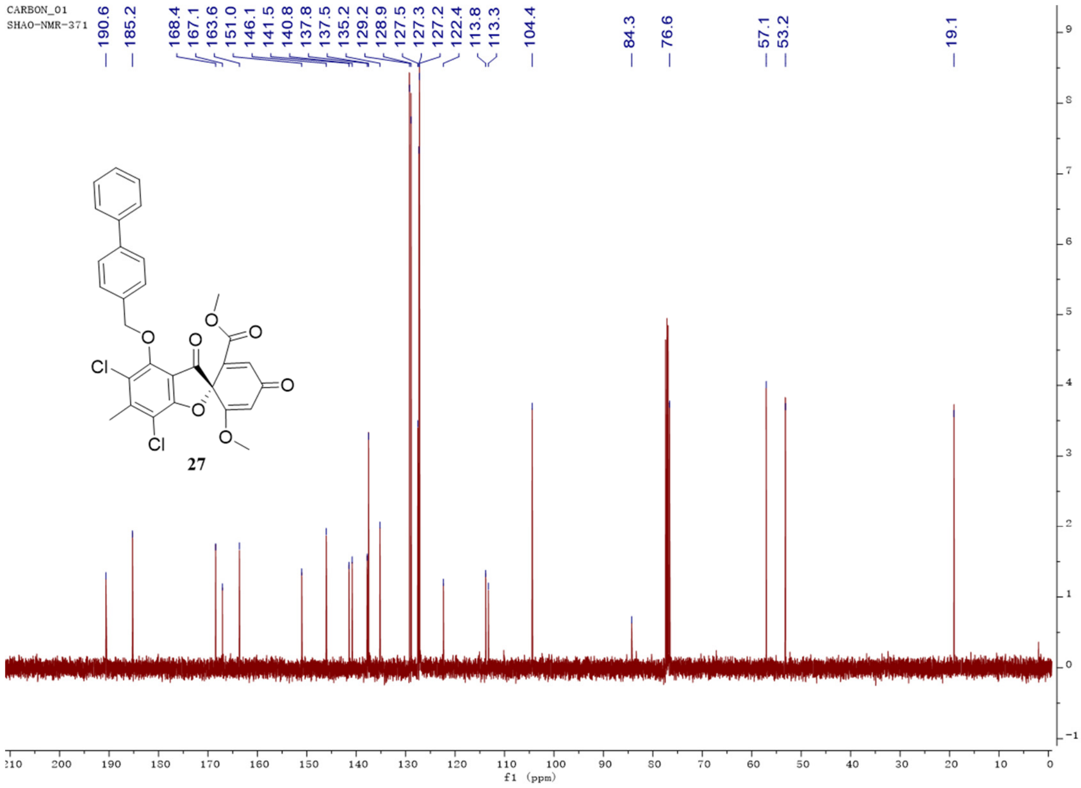

Figure S80. ^{13}C NMR (125 MHz, $CDCl_3$) spectrum of compound **27**

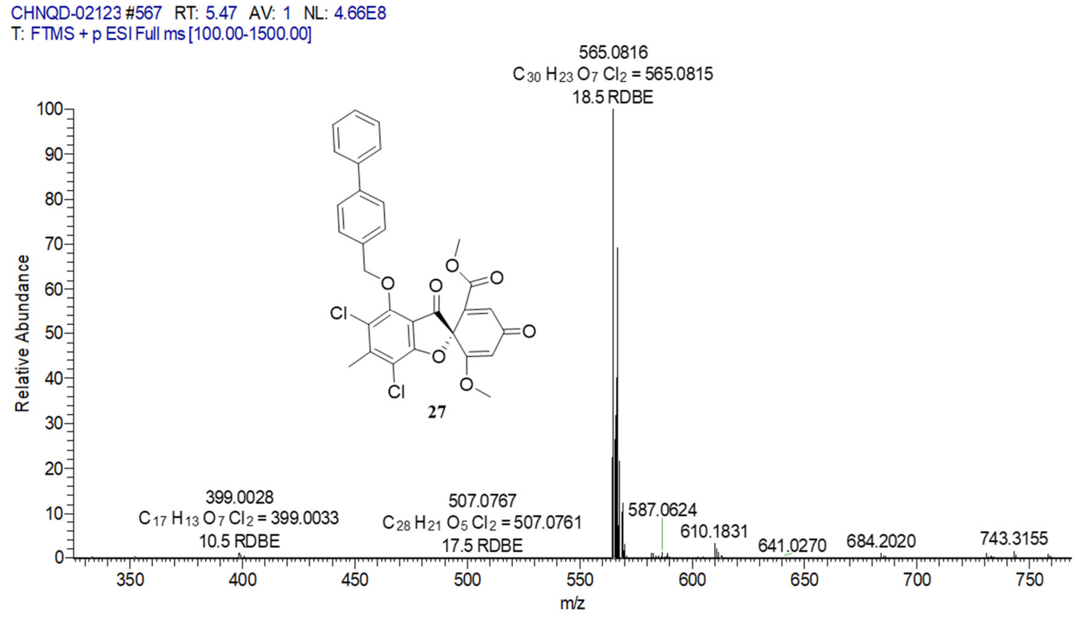

Figure S81. HRESIMS spectrum of compound **27**

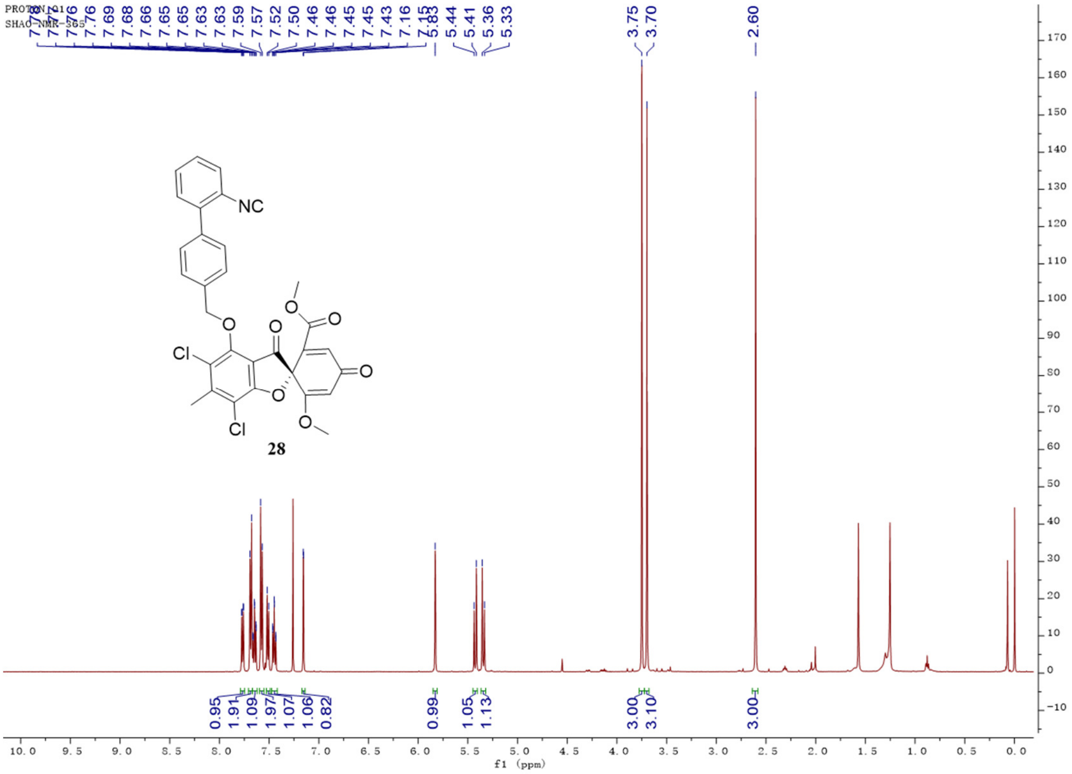

Figure S82. ^{1}H NMR (500 MHz, $CDCl_3$) spectrum of compound **28**

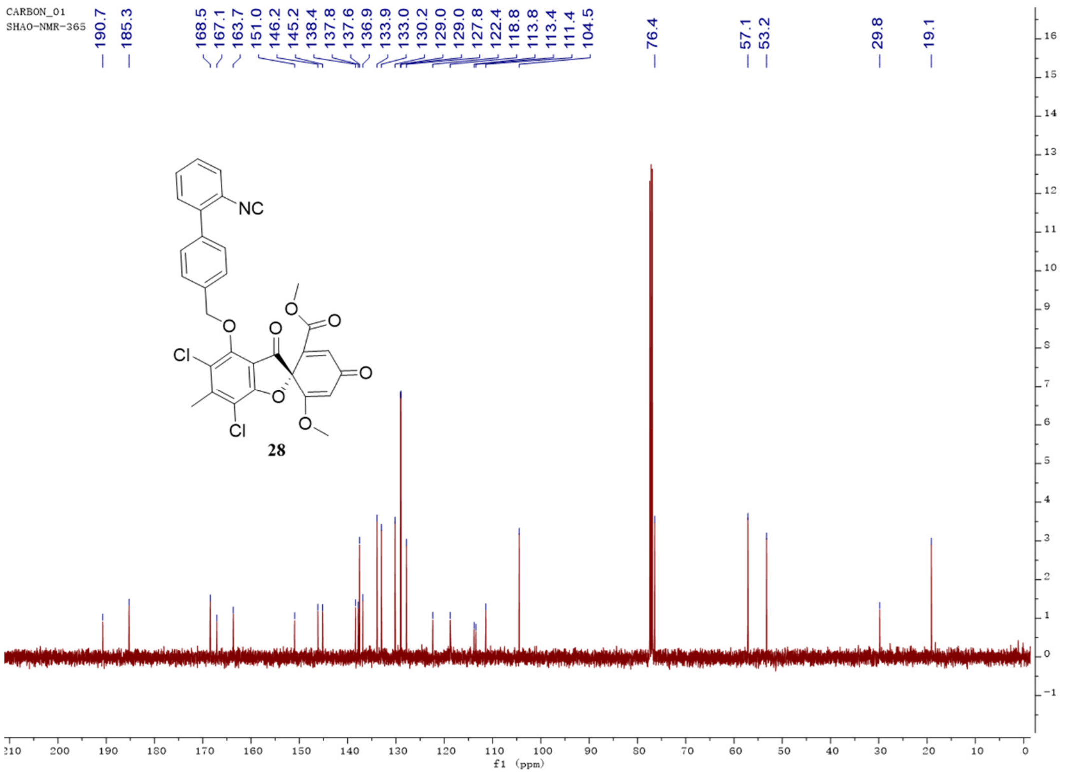

Figure S83. ^{13}C NMR (125 MHz, $CDCl_3$) spectrum of compound **28**

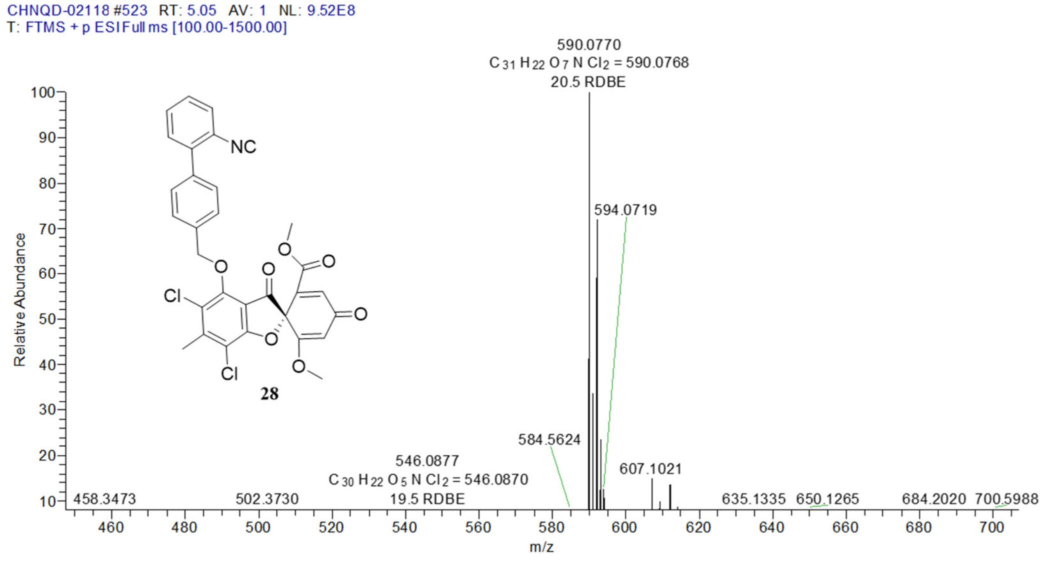

Figure S84. HRESIMS spectrum of compound **28**

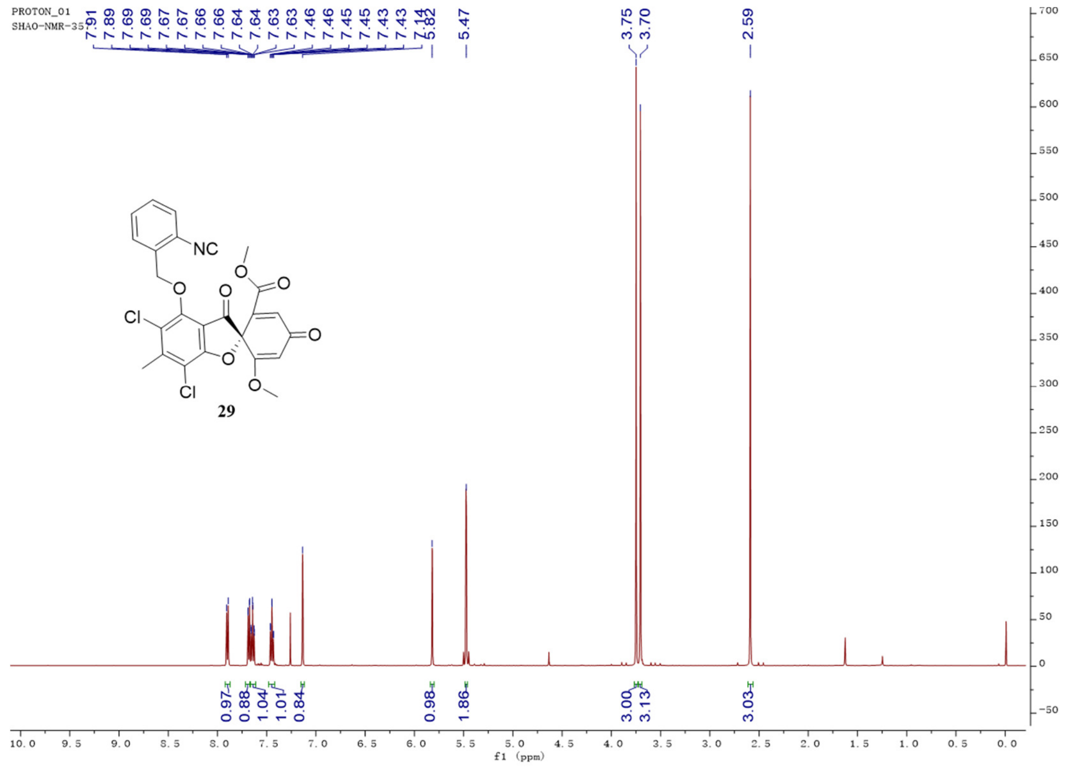

Figure S85. ^{1}H NMR (500 MHz, $CDCl_3$) spectrum of compound **29**

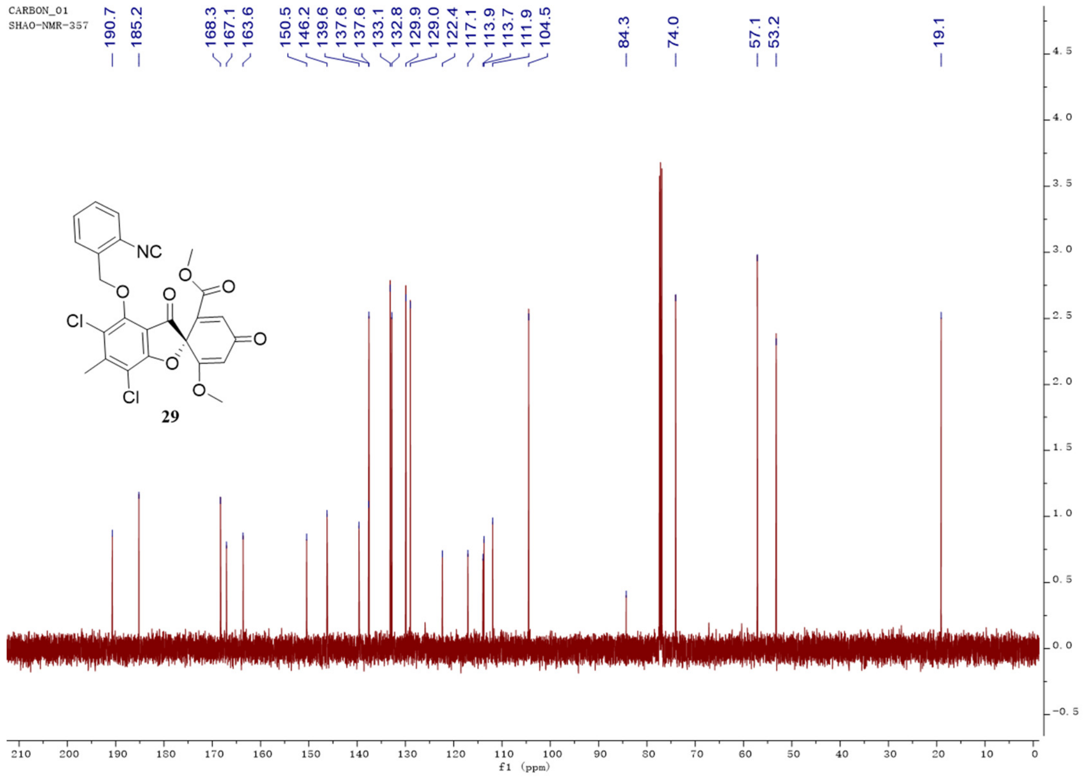

Figure S86. ^{13}C NMR (125 MHz, $CDCl_3$) spectrum of compound **29**

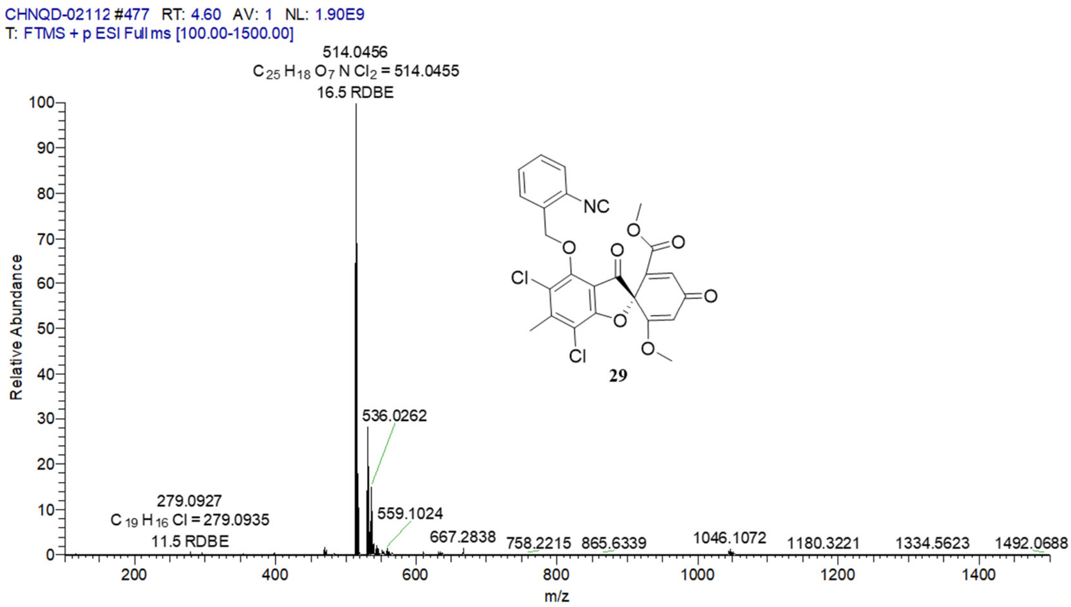

Figure S87. HRESIMS spectrum of compound **29**

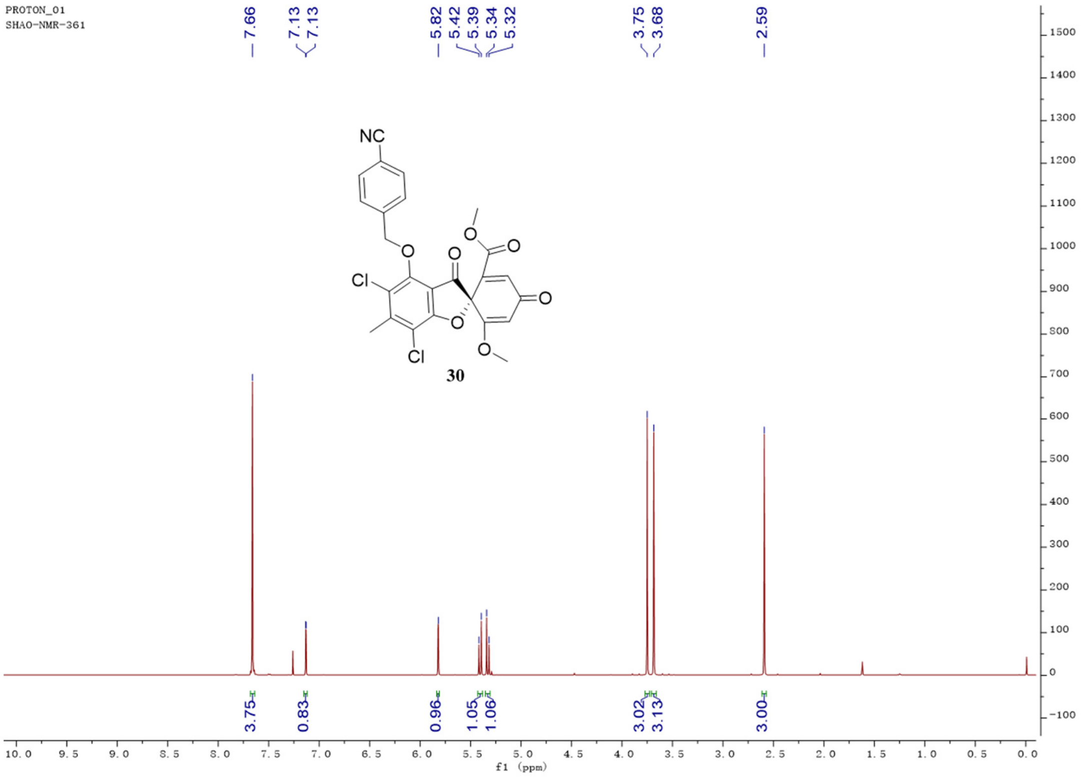

Figure S88. ¹H NMR (500 MHz, $CDCl_3$) spectrum of compound **30**

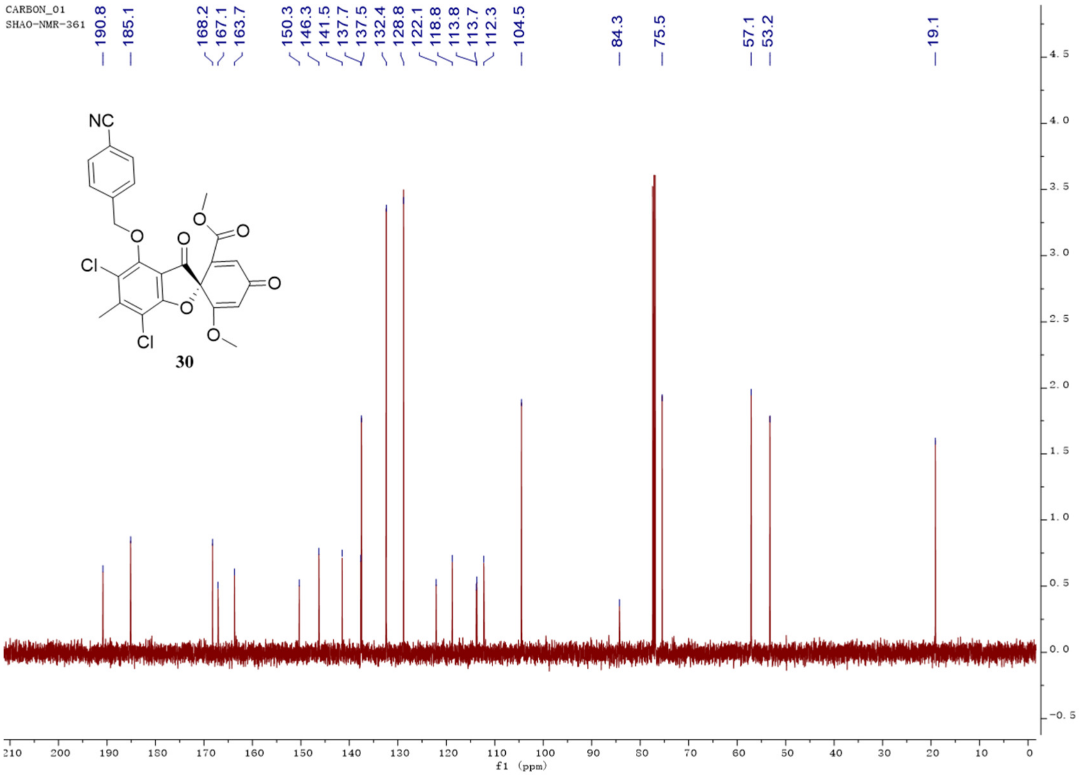

Figure S89. ^{13}C NMR (125 MHz, $CDCl_3$) spectrum of compound **30**

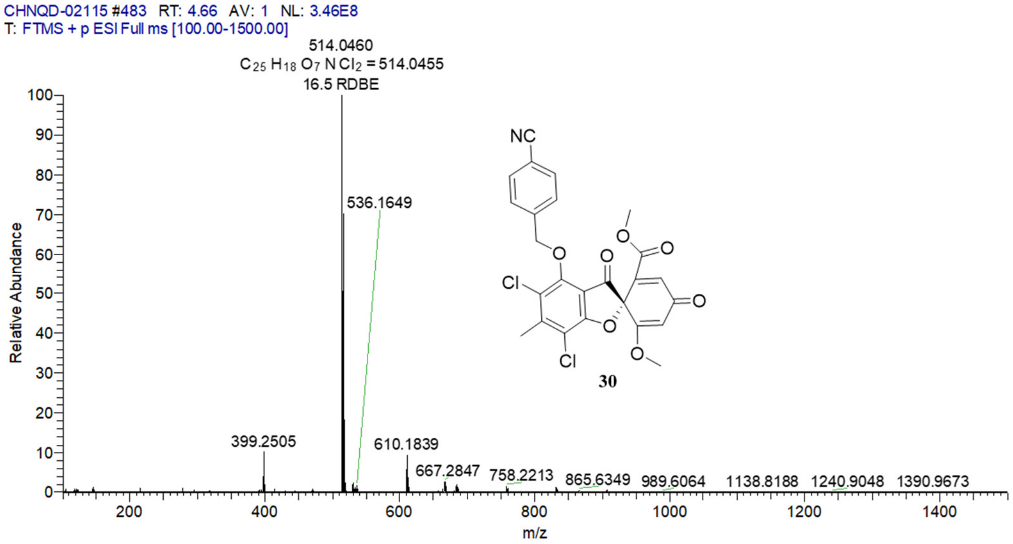

Figure S90. HRESIMS spectrum of compound **30**

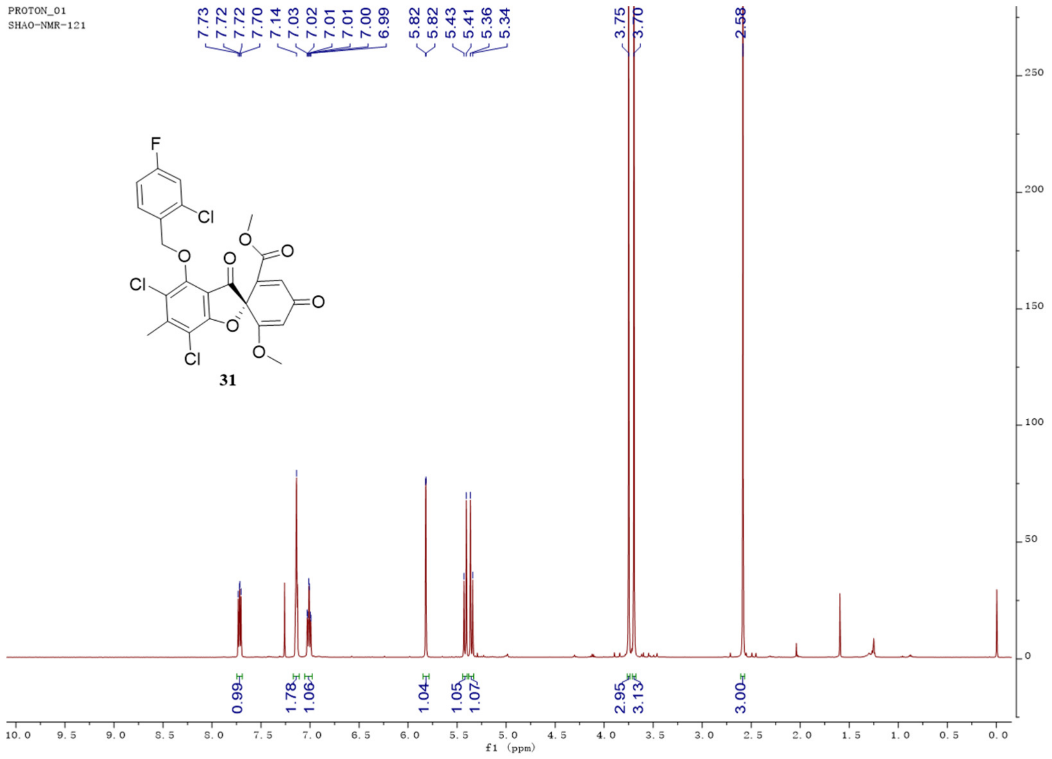

Figure S91. ^{1}H NMR (500 MHz, $CDCl_3$) spectrum of compound **31**

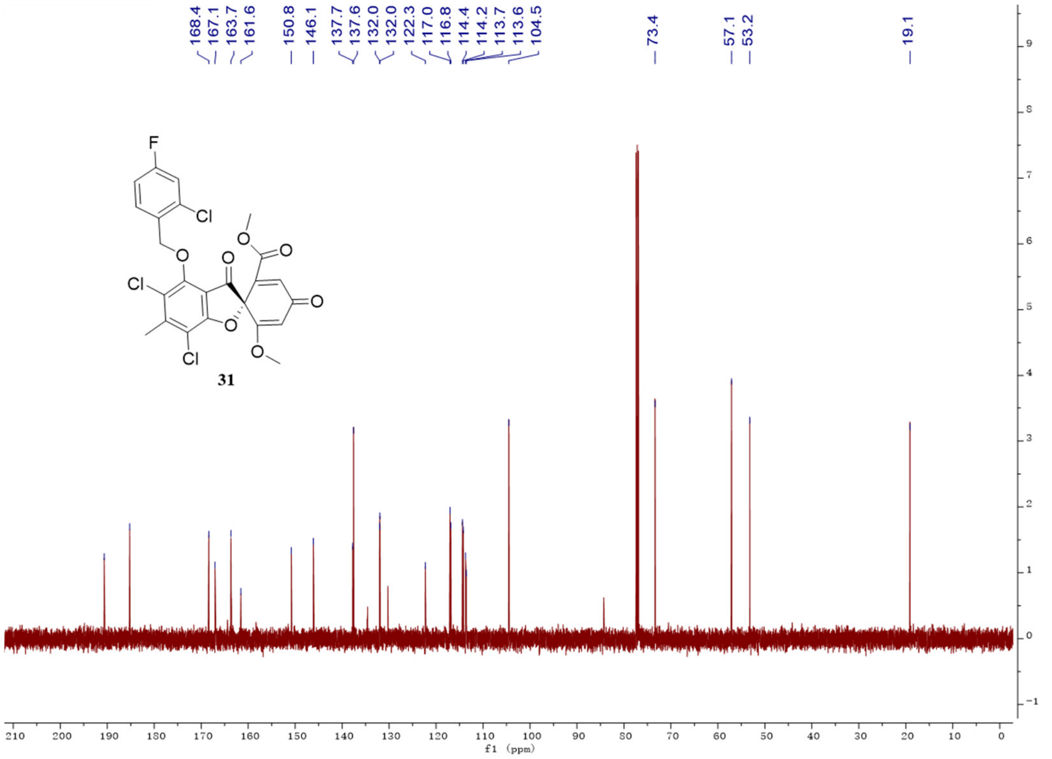

Figure S92. ^{13}C NMR (125 MHz, $CDCl_3$) spectrum of compound **31**

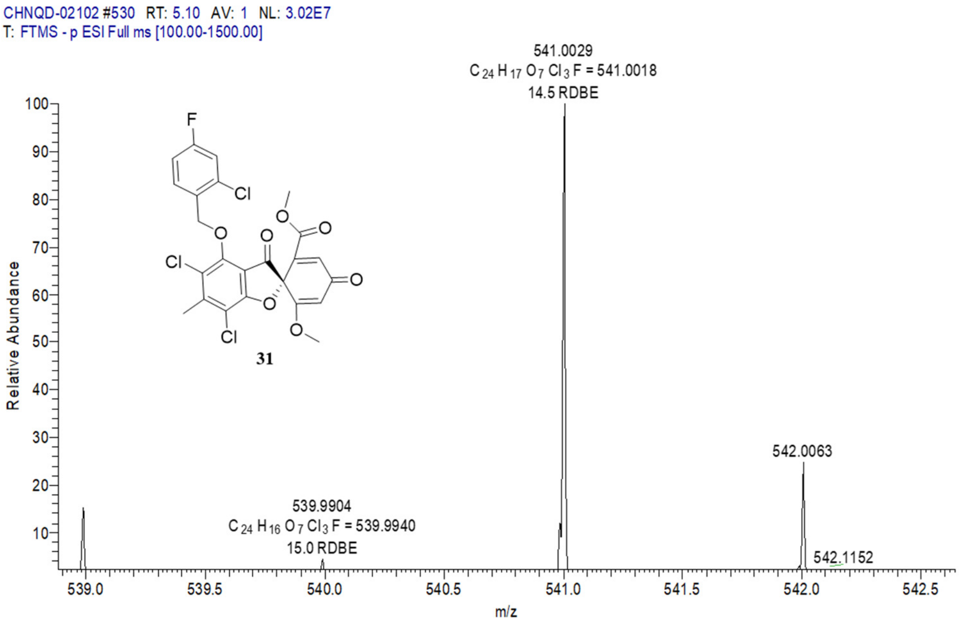

Figure S93. HRESIMS spectrum of compound **31**

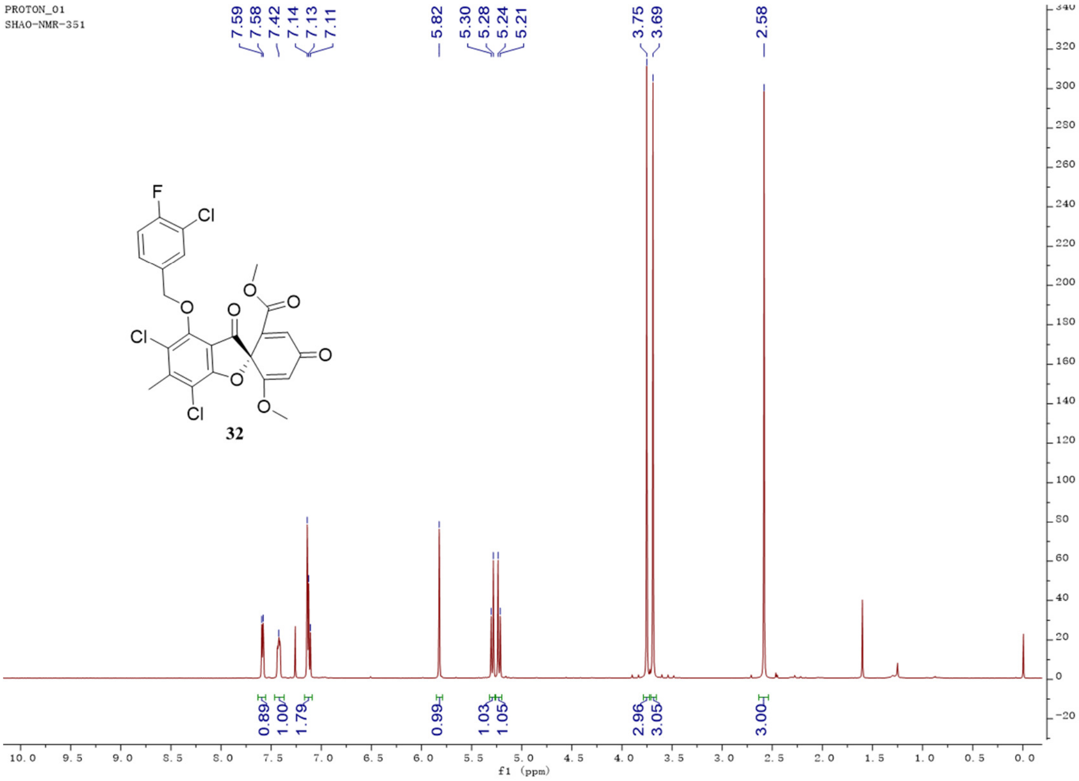

Figure S94. ^{1}H NMR (500 MHz, $CDCl_3$) spectrum of compound **32**

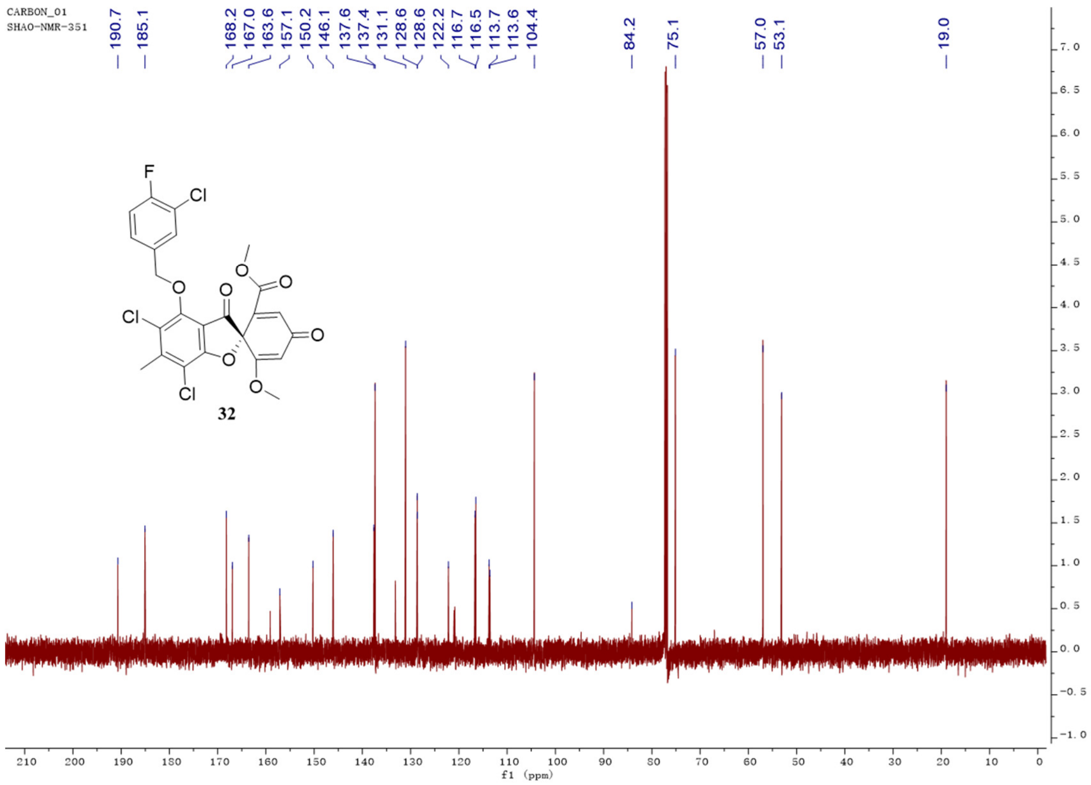

Figure S95. ^{13}C NMR (125 MHz, $CDCl_3$) spectrum of compound **32**

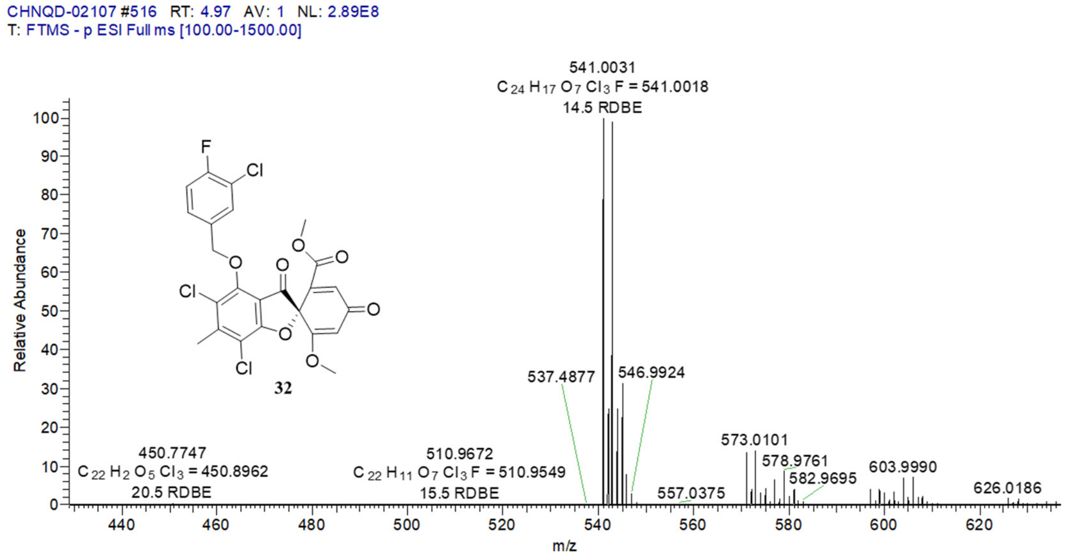

Figure S96. HRESIMS spectrum of compound **32**

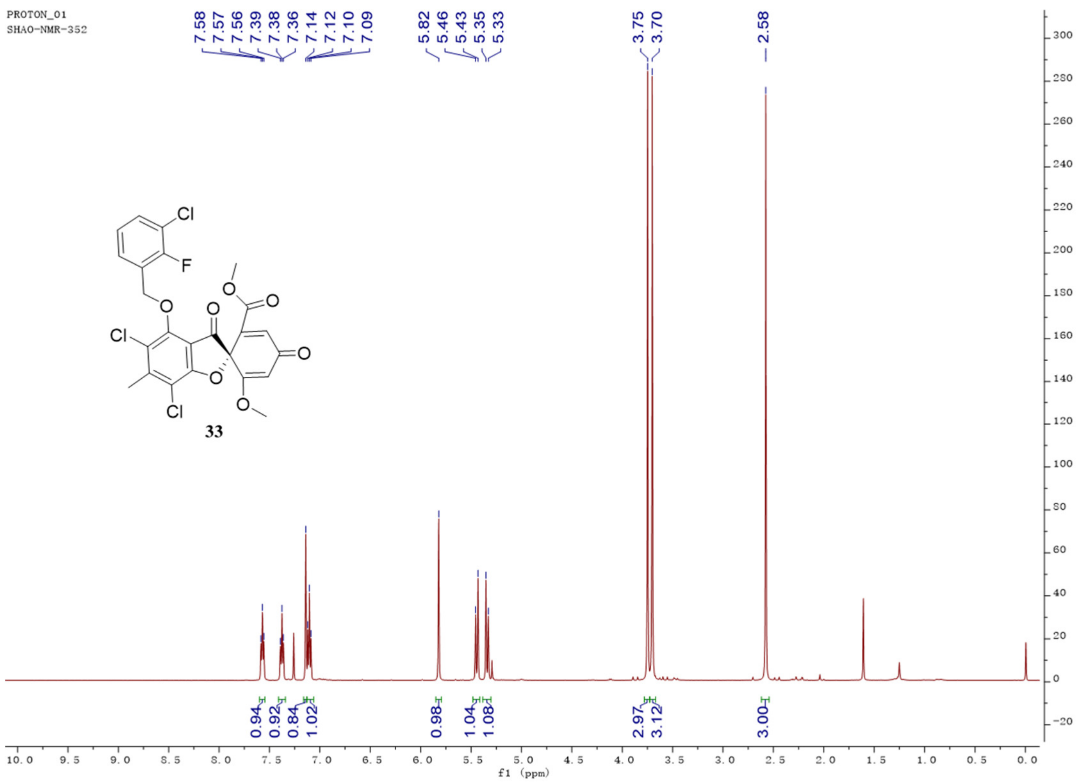

Figure S97. ^{1}H NMR (500 MHz, $CDCl_3$) spectrum of compound **33**

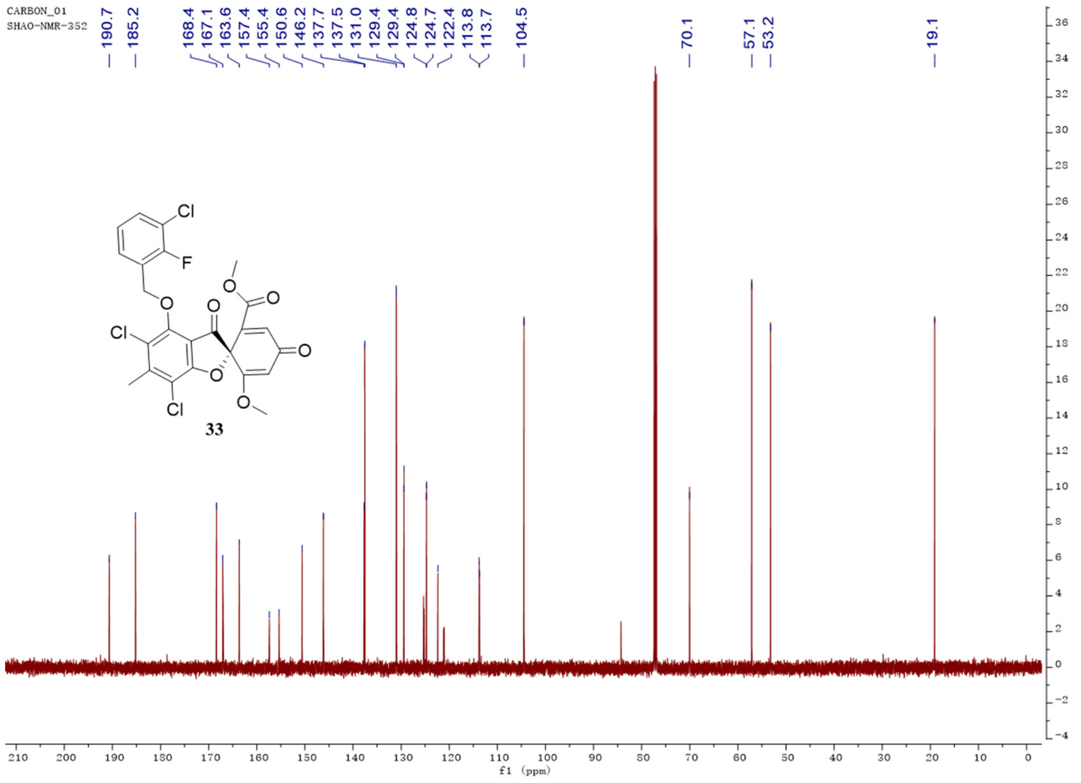

Figure S98. ^{13}C NMR (125 MHz, $CDCl_3$) spectrum of compound **33**

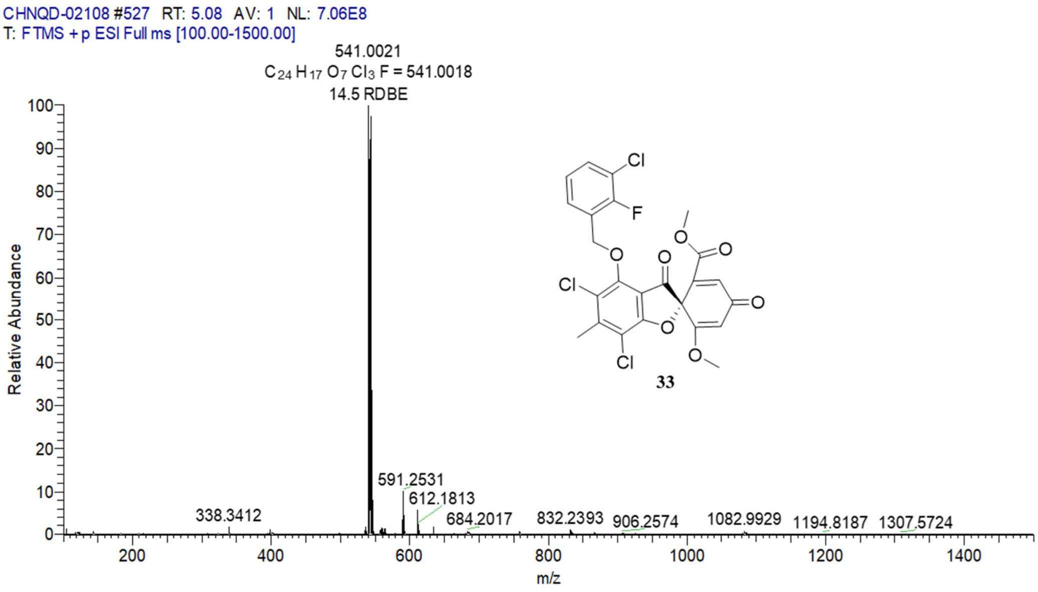

Figure S99. HRESIMS spectrum of compound **33**

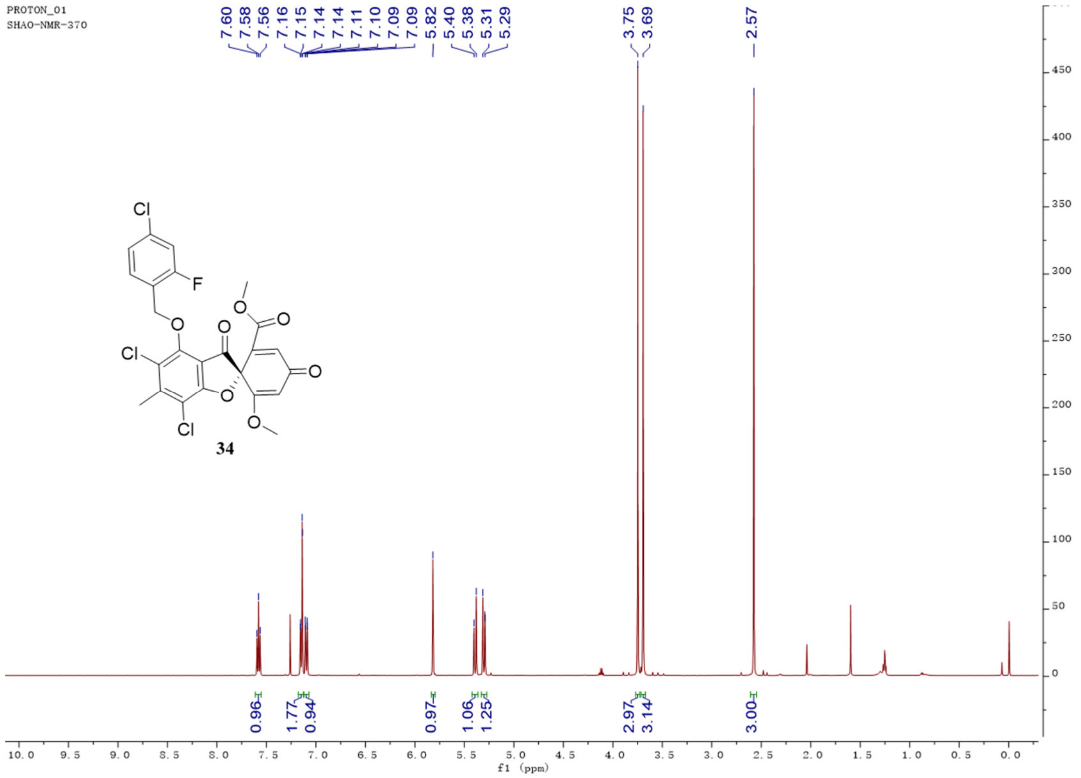

Figure S100. ^{1}H NMR (500 MHz, $CDCl_3$) spectrum of compound **34**

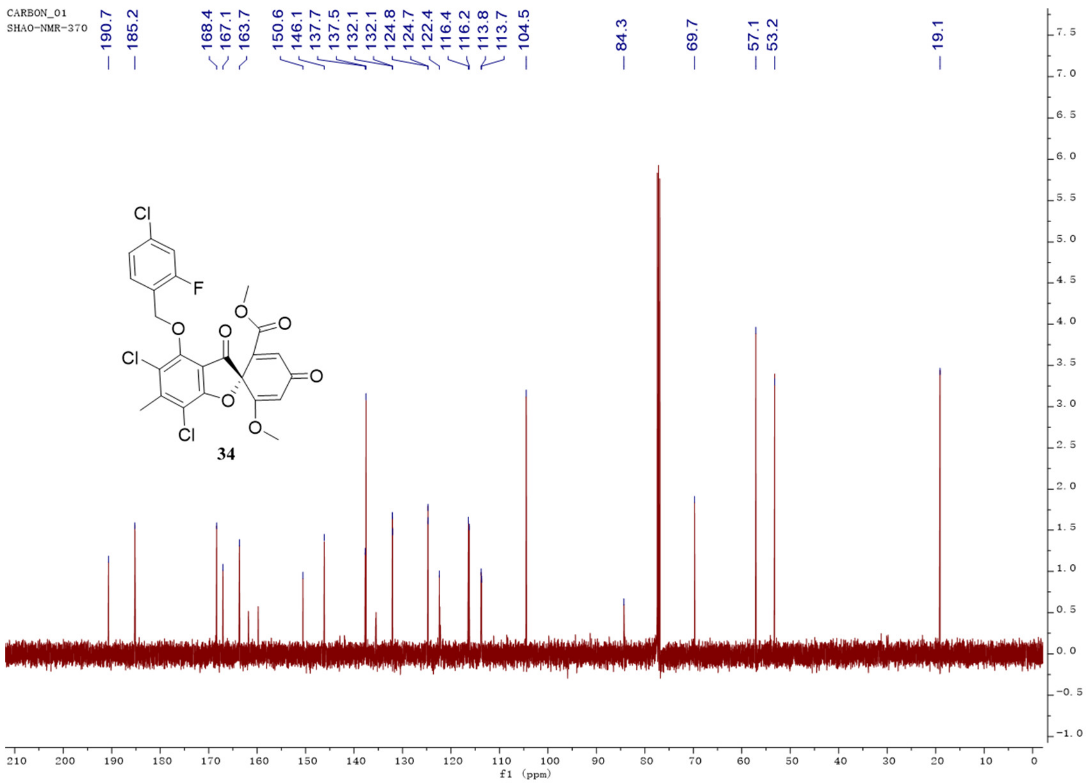

Figure S101. ^{13}C NMR (125 MHz, $CDCl_3$) spectrum of compound **34**

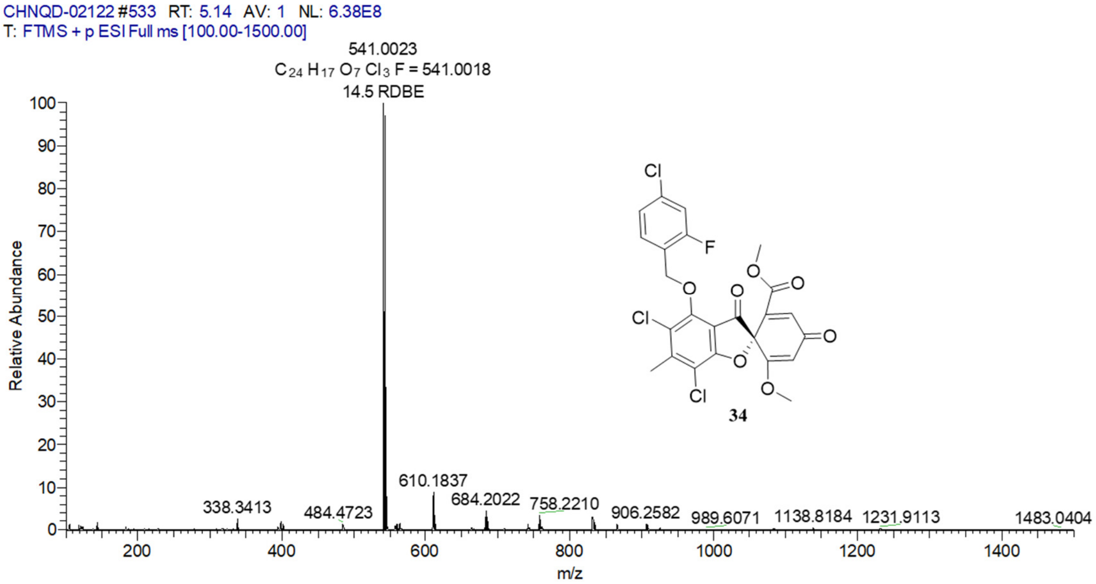

Figure S102. HRESIMS spectrum of compound **34**

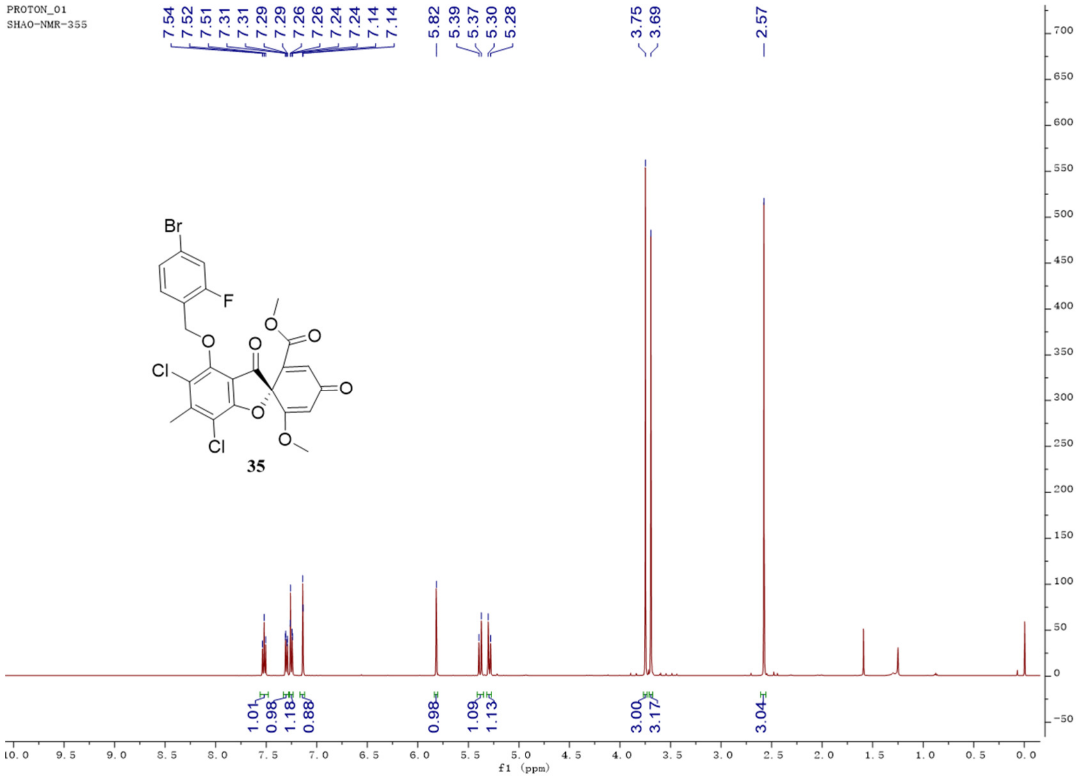

Figure S103. ^{1}H NMR (500 MHz, $CDCl_3$) spectrum of compound **35**

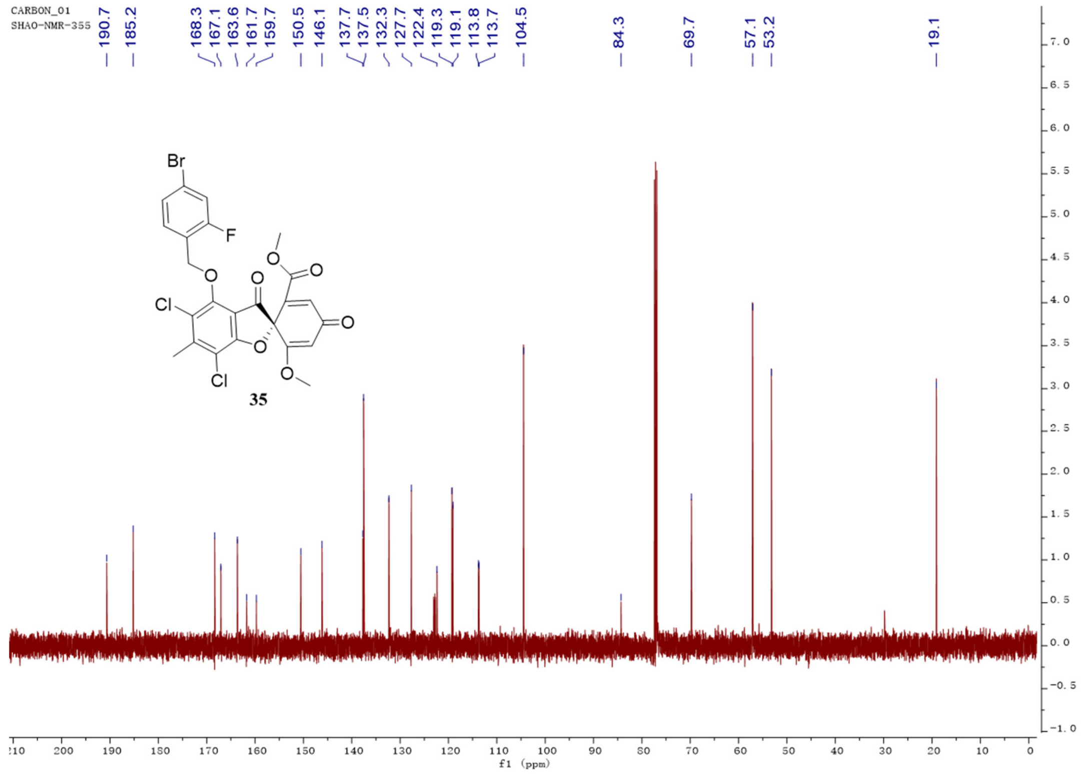

Figure S104. ^{13}C NMR (125 MHz, $CDCl_3$) spectrum of compound **35**

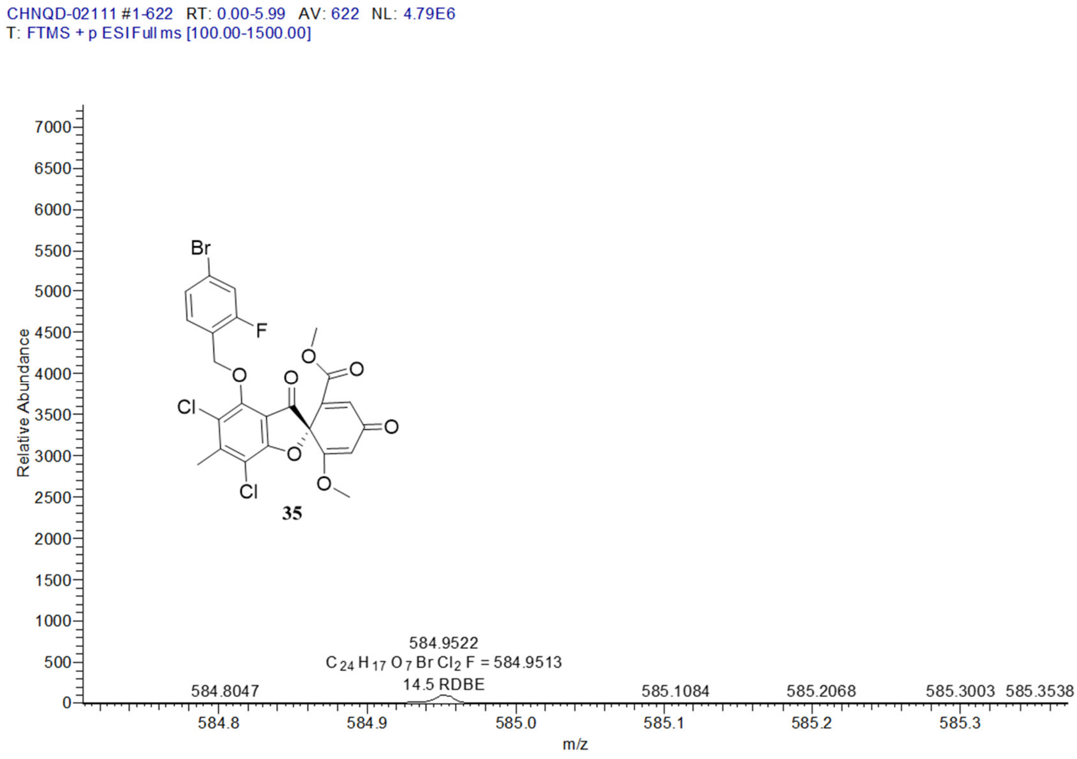

Figure S105. HRESIMS spectrum of compound **35**

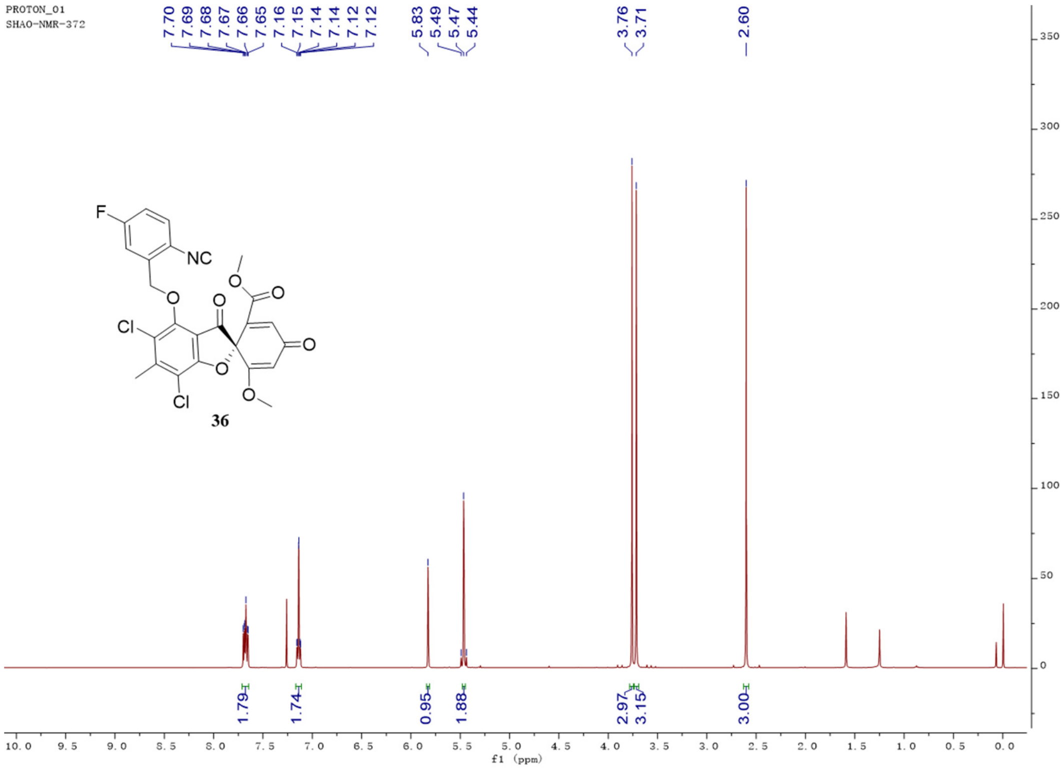

Figure S106. ^{1}H NMR (500 MHz, $CDCl_3$) spectrum of compound **36**

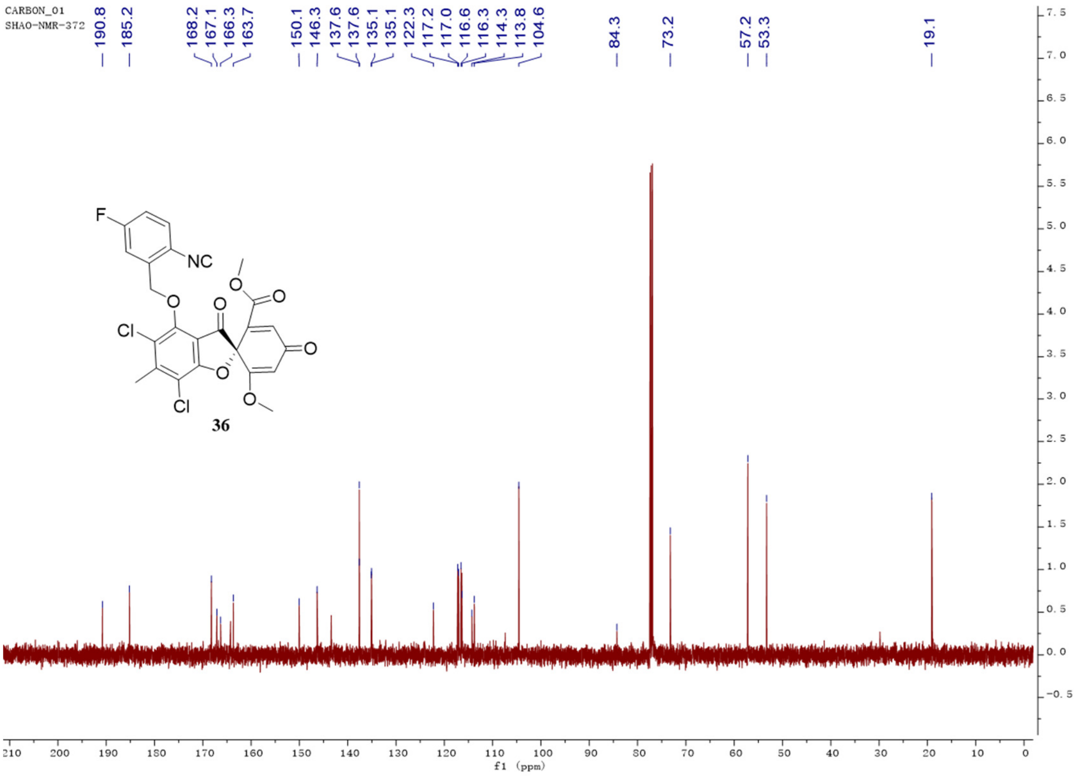

Figure S107. ^{13}C NMR (125 MHz, $CDCl_3$) spectrum of compound **36**

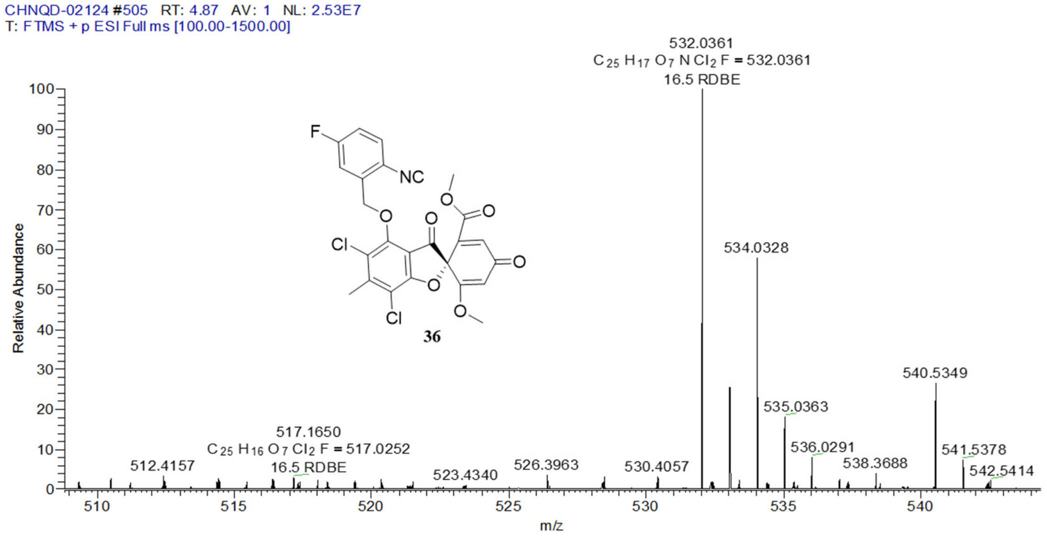

Figure S108. HRESIMS spectrum of compound **36**

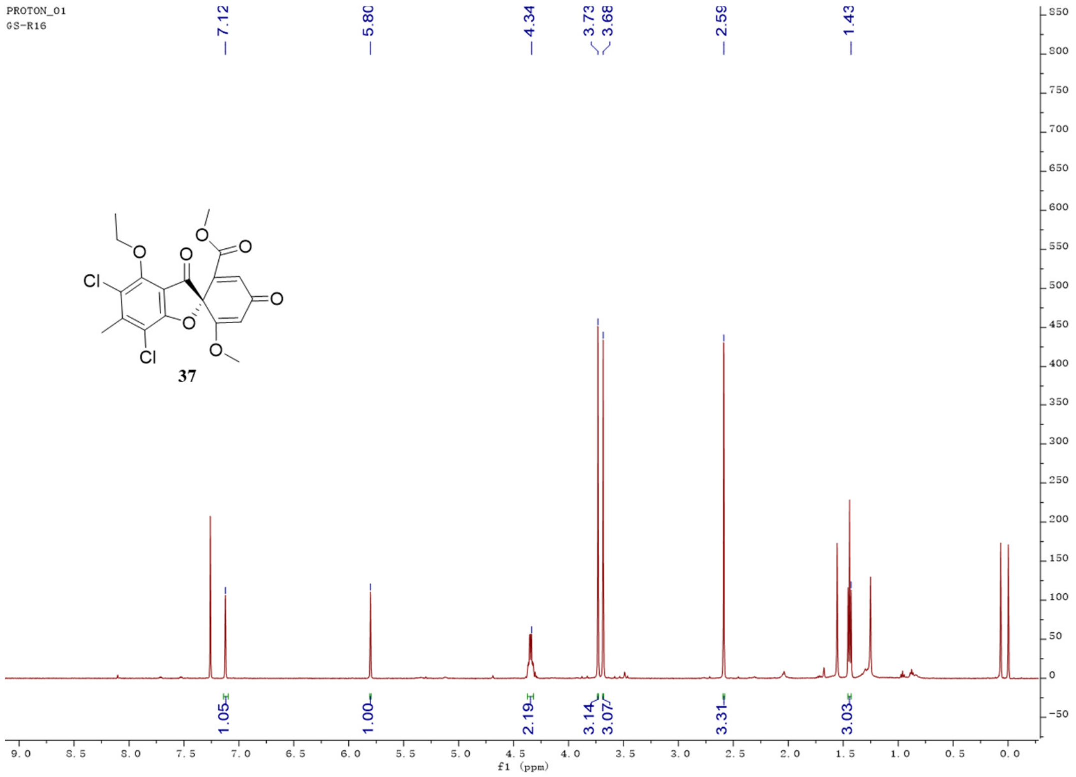

Figure S109. ^{1}H NMR (500 MHz, $CDCl_3$) spectrum of compound **37**

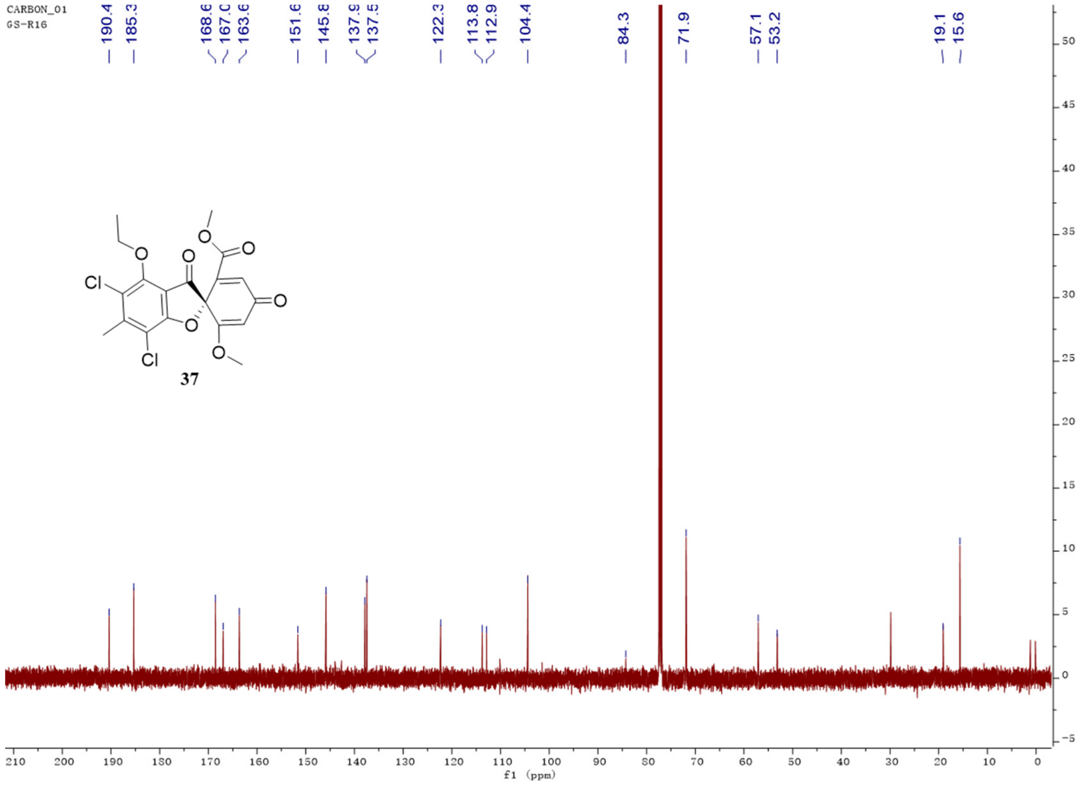

Figure S110. ^{13}C NMR (125 MHz, $CDCl_3$) spectrum of compound **37**

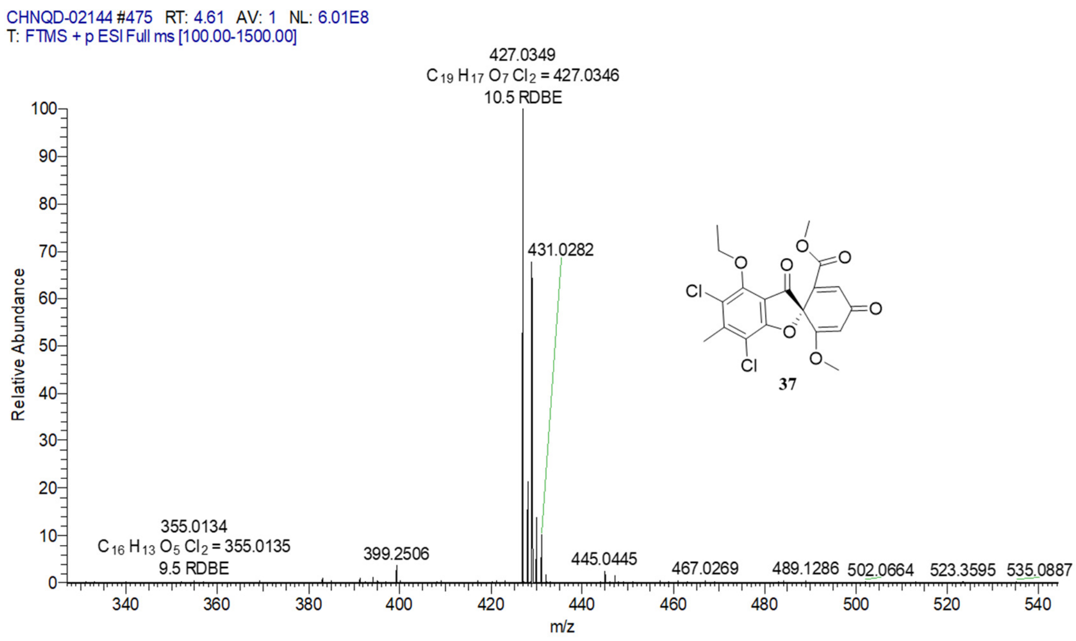

Figure S111. HRESIMS spectrum of compound **37**

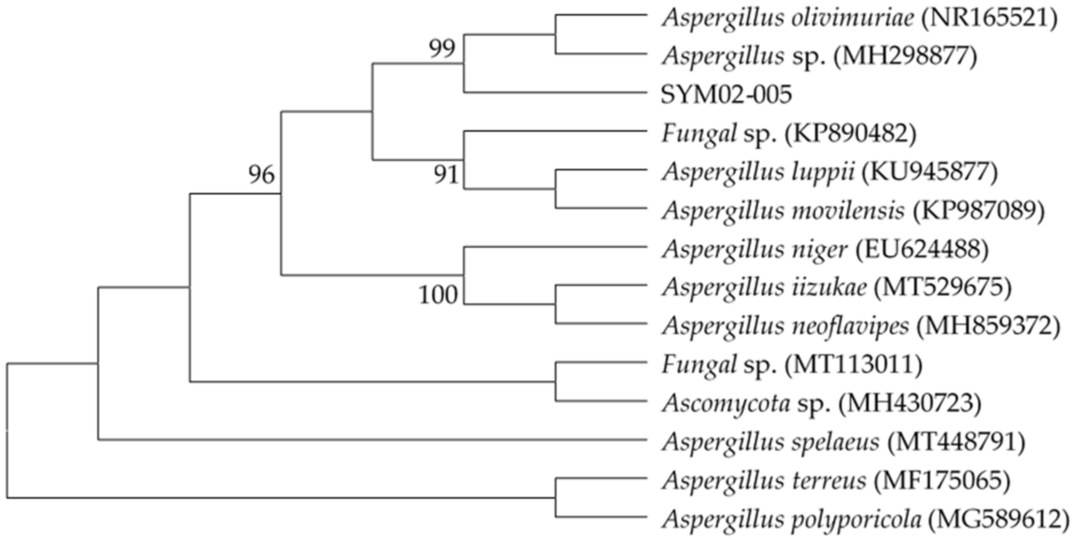

Figure S112. Neighbor-joining phylogenetic tree based on ITS sequences of cultured fungi associated with the fungus SYM02-005. The values at each node represent the bootstrap values from 1,000 replicates, and the scale bar=0.05 substitutions per nucleo.
